# Enantioselective isothiourea-catalysed α-fluorination of C1-ammonium enolates generated from arylesters

**DOI:** 10.1016/j.jfluchem.2025.110434

**Published:** 2025-04

**Authors:** Nicole Stanek, Lotte Stockhammer, Anja Moser, Mario Waser

**Affiliations:** Institute of Organic Chemistry, Johannes Kepler University Linz, Altenbergerstr. 69, 4040 Linz, Austria

**Keywords:** Organocatalysis, Lewis bases, Isothioureas, Fluorination

## Abstract

We herein report the chiral isothiourea-catalyzed α-fluorination of electron-deficient arylesters. This transformation proceeds via the in situ formation of chiral C1-ammonium enolates, which then undergo the α-fluorination with high levels of enantioselectivities, followed by addition of alcohols, such as MeOH, to give the final chiral α-F-esters.

## 1 Introduction

**Scheme 1:**
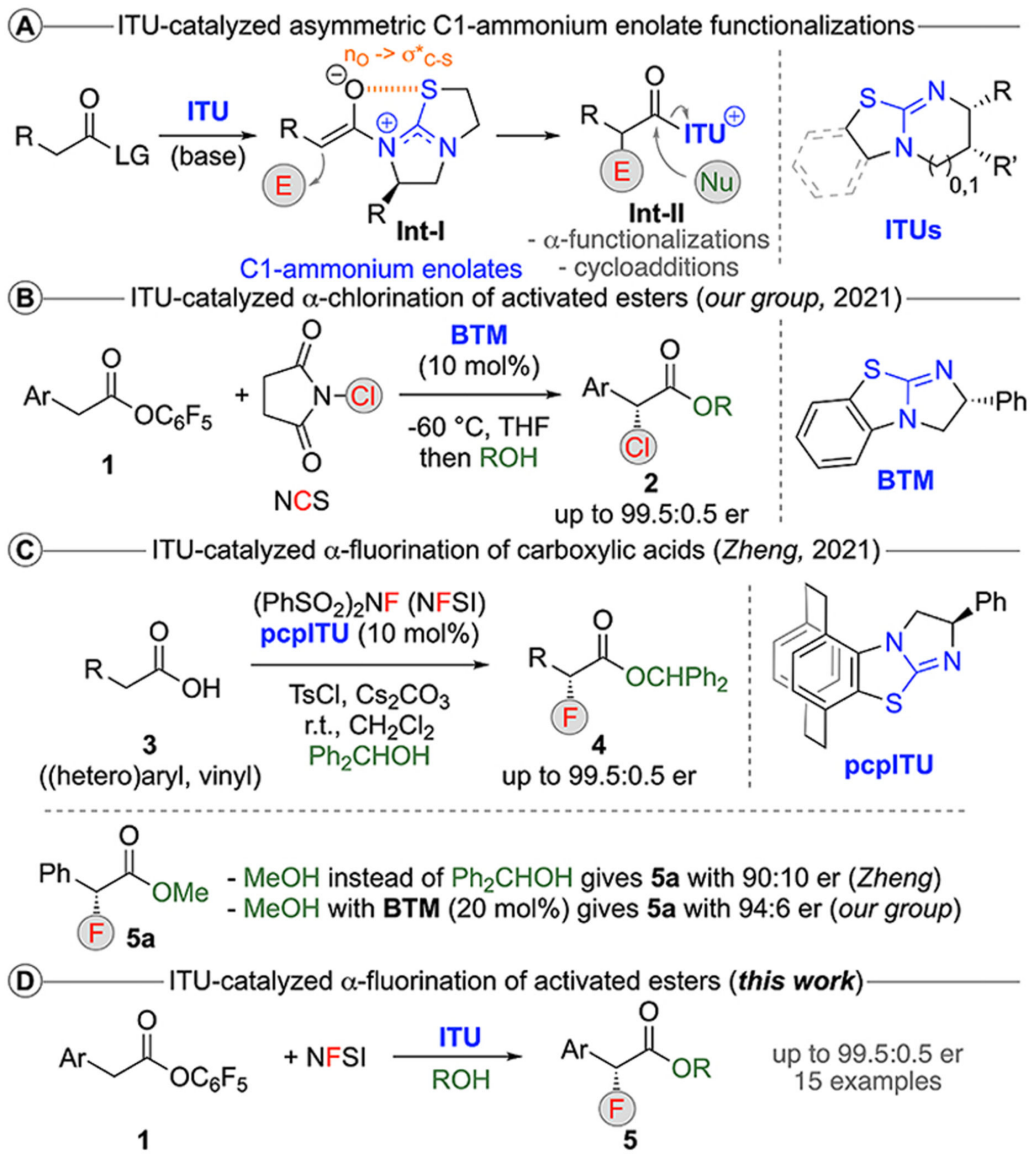
General reactivity of ITU-based C1-ammonium enolates (A), our recently developed α-chlorination (B) and Zheng’s α-fluorination (C) strategies, and the herein reported ITU-catalyzed α-fluorination of arylesters **3** (D).

The enantioselective synthesis of α-fluorinated carbonyl derivatives is a task of high value. As a consequence, numerous conceptually different strategies to introduce a fluorine atom in the α-position of a carbonyl compound have been reported.^1–3^ Over the years, a variety of different asymmetric catalysis principles that allow for the control of reactions between enolate-type species and electrophilic F-transfer reagents have been reported. Organocatalysis has contributed significantly to the advancement of the field, either relying on the use of non-covalent (e.g. chiral ion pairing) or covalent (i.e. chiral Lewis base) activation modes.^1–3^ In principle, the use of chiral Lewis base (LB) catalysts,^4^ which covalently bind to the starting material, is a very powerful strategy to achieve high levels of stereoinduction in asymmetric α-functionalization reactions of carboxylic acid derivatives.^5^ The use of chiral *tert*. amines and pyridine-derivatives as LB catalysts to access enantioenriched α-F-carboxylic acid derivatives was pioneered by the groups of Lectka and Fu.^6,7^ An alternative class of chiral organocatalysts that holds much promise for the activation and use of carboxylic acid-based starting materials are isothioureas (ITUs).^8^ This class of easily accessible bench stable Lewis bases has been well established over the last two decades. By covalently binding electrophilic starting materials highly activated chiral intermediates will be generated, which can then undergo a multitude of transformations.^8^ One of the most important applications of these catalysts is the activation and control of enolisable carboxylic acid derivatives.^9^ Upon adding to activated carboxylic acid derivatives, such as acylhalides, mixed anhydrides, or electron-deficient arylesters, and sub-sequent α-deprotonation chiral C1-ammonium enolates (**Int-I**) are generated (Scheme 1A, the terminology *C1-ammonium enolate* describes the fact that the ammonium species (the catalyst) is covalently attached to carbon one of the substrate, the carbonyl group).^9^ These species benefit from a very well-defined “locked” conformation (stabilized by a pronounced n_O_→σ*_C-S_ interaction between the enolate’s oxygen and the catalyst’s sulfur) shielding one face of the enolate.^10^ This allows for predictable selectivities in subsequent α-functionalizations which first give catalyst-bound α-functionalized species **Int-II**, followed by a final catalyst liberation by addition of a nucleophilic species. Our group has been interested in this activation mode for a while^11,12^ and recently we developed methods for the direct highly enantioselective α-chlorination of activated arylacetic esters **1** by using **BTM** as a chiral LB catalyst (Scheme 1B).^12^ Simultaneously, Zheng’s group introduced a very interesting strategy for the direct α-fluorination of free carboxylic acids **3** by using TsCl to generate an activated mixed anhydride in situ, which then reacts with the ITU to the reactive C1-ammonium enolate.^13^ Hereby, the newly designed **pcpITU** was found to be the catalyst of choice which, in combination with benzhydrol (Ph_2_CHOH) as a trapping agent, allows for high enantioselectivities in the syntheses of products **4** (Scheme 1C). Interestingly, when using MeOH instead, Zheng and co-workers observed a reduced enantioselectivity. We also attempted the synthesis of **5a** but with **BTM** instead of **pcpITU** and obtained the product in slightly higher selectivity as compared to the use of **pcpITU**, but lower than in the benzhydrol approach (Scheme 1C). Based on these observations, and our own experience using activated esters **1** as starting materials, we now wondered whether it is possible to carry out the enantioselective α-fluorination of esters **1** using simple ITU catalysts (Scheme 1D).

## 2 Results and discussion

**Scheme 2:**
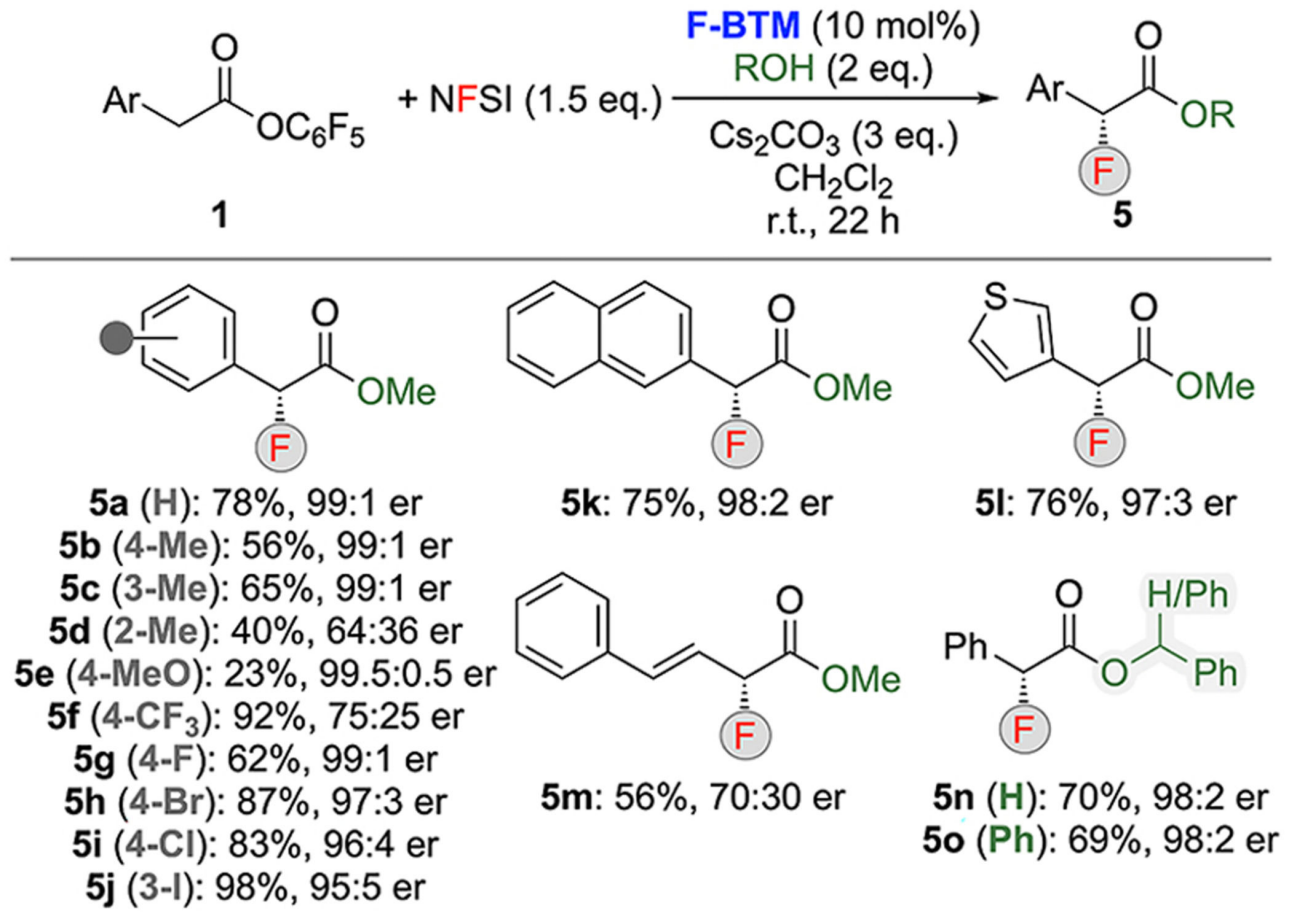
Asymmetric application scope.

We started our investigations by testing the ITU-catalyzed reaction of the parent ester **1a** and NFSI in the presence of MeOH as a trapping agent (see Table 1 for an overview of the most significant results obtained hereby). First experiments with 20 mol % **BTM** in different solvents (entries 1–3) turned out to be very promising, delivering the α-fluorinated phenylacetic ester **5a** with 98:2 er and in reasonable yield (76 % NMR yield) when carrying out the reaction in CH_2_Cl_2_ (entry 1). Lowering the catalyst loading to 10 mol % resulted in a slightly reduced selectivity and yield only (entry 4). Testing the 6-ring-fused **HyperBTM** and **HBTM** next (entries 5 and 6) showed that they perform slightly less selective as compared to **BTM** (a similar trend in catalytic activity was recently observed for our analogous α-chlorinations^12^). Noteworthy, when using **F-BTM**^14^ the selectivity could be improved further (entry 7). Furthermore, when employing a slightly larger excess of 2 eq. MeOH (entry 8) the yield could be increased to 78 % (NMR yield). At this point it has to emphasized that product **5a** (as well as most of the other products **5** shown in Scheme 2) has a strong tendency to co-evaporate with solvents like CH_2_Cl_2_, heptane, and EtOAc (to mention those that were primarily used herein only). Thus, quantitative isolation in pure and dry form after workup and chromatographic purification was found to be difficult and always accompanied with losses (use of a Vigreux column during solvent evaporation helps reducing these losses). For **5a** obtained with 70–80 % NMR yield isolated yields of around 40 % after silica gel column chromatography were usually obtained. This loss in yield is because of (co-)evaporation with the solvent on the rotavapor as well as during drying on the Schlenk line. On the other hand, we did not observe any degradation during silica gel chromatography and we also found **5a** (and other products **5**) being (configurationally) stable when stored in solution. Concerning the configurational stability of products **5** it should be emphasized that we also did not observe any epimerization over prolonged reaction times under the basic reaction conditions (a possible epimerization however may happen on **Int-II**, but the direct MeOH quench prevents this efficiently (vide infra)). Finally, alternative bases were tested too (entries 9 and 10) but without any further improvement as compared to Cs_2_CO_3_ (it should be stated that lower amounts of Cs_2_CO_3_lead to reduced conversion). The superior performance of Cs_2_CO_3_ may be attributed to its reasonably high basicity, allowing for the quantitative enolate formation, combined with its good solubility (as compared to e.g. K_2_CO_3_) in the reaction medium.

Having identified suited conditions for the asymmetric synthesis of **5a** (entry 8, Table 1), we next investigated the generality of this protocol by using different esters **1** as well as two other alcohols for the trapping (Scheme 2). The substitution pattern of the aryl-groups had a strong impact on enantioselectivities and reaction yields. For example, a Megroup in the para- and meta-position did not noteworthy affect the outcome (see products **5b, c**), while the presence of an ortho-Me group (**5d**) lead to a significantly reduced selectivity of 64:36 er and yield. This observation is unfortunately in accordance to previous observations,^12b^ suggesting that this ortho-substituent obviously interferes in the stereodefining α-functionalization step. Electron-donating (MeO-, **5e**) as well as strongly electron-withdrawing (CF_3_-, **5f**) substituents also showed a strong impact. While the MeO-group “only” led to significantly reduced yield without affecting the er, the presence of the CF_3_ group caused a lower selectivity of 75:25 er, which can be rationalized by a higher tendency to epimizeriation of **Int-II**, due to the increase acidity of the α-position because of this strongly electron-withdrawing group. In contrast, the presence of halogens did not influence the outcome too drastically (**5 g-j**), although enantioselectivities were slightly lower as compared to the parent compound **5a**. Naphthyl- and thienyl-based products **5k** and **5l** were also equally well obtained while the styryl-derivative **5m** performed less selective. Furthermore, also two alternative alcohols were tested, which both gave the products **5n** and **5o** in comparable yields and selectivities as MeOH (**5a**). These results indicate that the addition of the different alcohols to the intermediate α-fluorinated catalyst bound species **Int-II** (compare with Scheme 1A) is a fast process, which prevents epimerization of this intermediate (this was actually an issue in our previous α-chlorination^12a^). It should be stated that, due to the volatility of most of the products and for the sake of comparison of the reaction performance, NMR yields are given in Scheme 2. However, depending on the substitution pattern, the isolated yields for pure and clean products differ more or less compared to the NMR yields.^16^ While this deviation was found to be not too striking for “heavier” products such as **5h, 5k, 5n** or **5o** others, such as the F-substituted **5f** and **5 g** turned out to be even more volatile as compared to **5a** (again it should be stated that the compounds are stable in solution).^16^

**Scheme 3:**
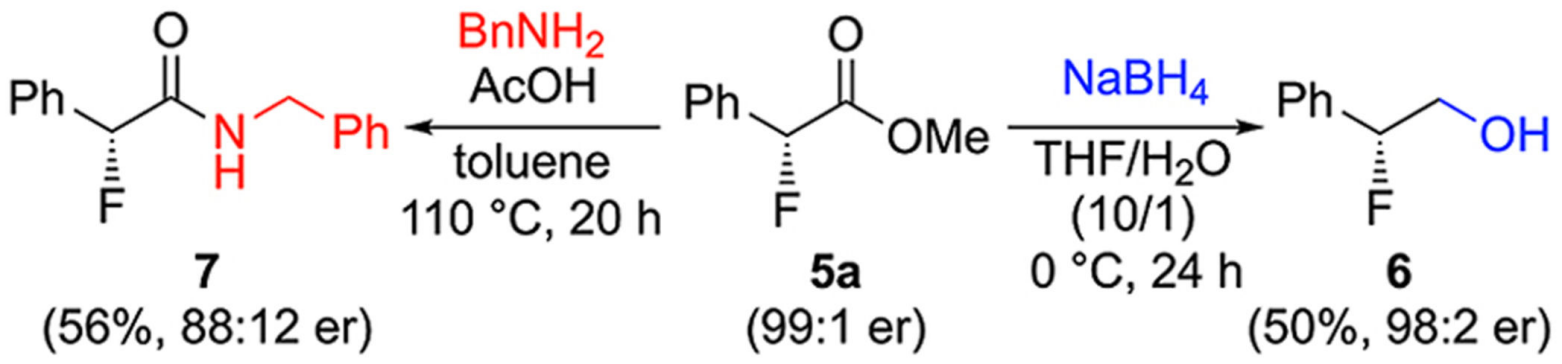
Further ester group manipulations.

Finally, we also tested the suitability of products **5** for ester group manipulations. As shown in Scheme 3, it is possible to carry out the direct reduction to alcohol **6** as well as the synthesis of amide **7** under unoptimized standard reduction/amidation conditions. Interestingly, in both cases some racemization was observed (more pronounced for product **7**), which can be rationalized by a pronounced acidity of the α-position. It should be emphasized that these transformations only serve as a first proof-of-concept, but no further optimization efforts to increase yield and suppress racemization were undertaken.

## 3 Conclusion

The direct isothiourea-catalyzed α-fluorination of electron-deficient esters **1** via in situ generated C1-ammonium enolates allows for the synthesis of α-F-esters **5** with good to excellent levels of enantiose-lectivities. **F-BTM** was found to be the catalyst of choice in combination with MeOH as the trapping reagent. Other alcohols were tolerated equally well but interestingly, the nature of the aryl-substituents has a strong impact on the enantioselectivity of this protocol. One considerable aspect is however the observed volatility of products **5**, which clearly affected the isolated yields hereby. This is a point which, in our opinion, has a more drastic effect on smaller lab scale experiments then compared to larger scale applications, where distillations can usually be controlled much more efficiently and where product losses will be smaller as compared to smaller scale experiments. Furthermore, strongly basic downstream manipulations may lead to partial epimerization of the products, thus requiring special care and optimization.

## Supplementary Material

Supplementary material associated with this article can be found, in the online version, at 10.1016/j.jfluchem.2025.110434.

SI

## Figures and Tables

**Table 1:** Optimization of the fluorination of ester 1a^a^.

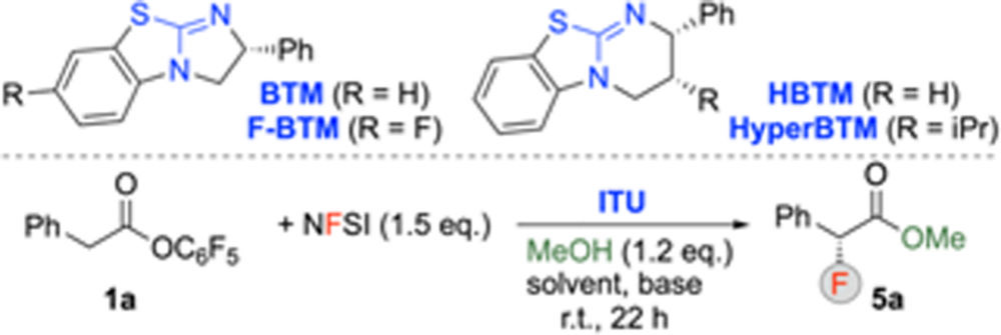
entry	ITU (mol %)	base	solv.	yield^b^ [%]	er^c^
1	BTM (20)	Cs_2_CO_3_	CH_2_Cl_2_	76	98:2
2	BTM (20)	Cs_2_CO_3_	THF	36	98:2
3	BTM (20)	Cs_2_CO_3_	toluene	59	89:11
4	BTM (10)	Cs_2_CO_3_	CH_2_Cl_2_	73	96:4
5	HBTM (10)	Cs_2_CO_3_	CH_2_Cl_2_	47	96:4
6	HyperBTM (10)	Cs_2_CO_3_	CH_2_Cl_2_	65	95:5
7	FBTM (10)	Cs_2_CO_3_	CH_2_Cl_2_	58	99:1
8^d^	FBTM (10)	Cs_2_CO_3_	CH_2_Cl_2_	78	99:1
9^d^	FBTM (10)	K_2_CO_3_	CH_2_Cl_2_	76	94:6
10^d^	FBTM (10)	Et_3_N	CH_2_Cl_2_	6	n.d.

a Unless otherwise stated all reactions were run for 22 h at room temperature by using 0.1 mmol **1a**, 0.15 mmol NFSI, 0.3 mmol base and 0.12 mmol MeOH in the presence of the indicated ITU catalyst.

b NMR yields determined by ^19^F-NMR using fluorobenzene as an internal standard.

c Determined by HPLC using a chiral stationary phase. The absolute configuration was assigned by comparison of the optical rotation with literature reported values.^6a,15^.

d Using 2 eq. MeOH.

## Data Availability

The authors are unable or have chosen not to specify which data has been used.

## References

[1] Arimitsu S, Cahard D, Paquin JF (2024). Science of Synthesis.

[2] Ma JA, Cahard D (2008). Chem Rev.

[3] France S, Weatherwax A, Lectka T (2005). Eur J Org Chem.

[4] Denmark SE, Beutner GL (2008). Angew Chem Int Ed.

[5] Gaunt MJ, Johansson CCC (2007). Chem Rev.

[6] Paull DH, Scerba MT, Alden-Danforth E, Widger LR, Lectka T (2008). J Am Chem Soc.

[7] Lee SY, Neufeind S, Fu GC (2014). J Am Chem Soc.

[8] Taylor JE, Bull SD, Williams JMJ (2012). Chem Soc Rev.

[9] McLaughlin C, Smith AD (2021). Chem Eur J.

[10] Nagao Y, Hirata T, Goto S, Sano S, Kakehi A, Iizuka K, Shiro M (1998). J Am Chem Soc.

[11] Stockhammer L, Craik R, Monkowius U, Cordes DB, Smith AD, Waser M (2023). ChemistryEurope.

[12] Stockhammer L, Weinzierl D, Waser M (2021). Org Lett.

[13] Yuan S, Liao C, Zheng WH (2021). Org Lett.

[14] Young CM, Stark DG, West TH, Taylor JE, Smith AD (2016). Angew Chem Int Ed.

[15] Ishihara K, Nishimura K, Yamakawa K (2020). Angew Chem Int Ed.

[R16] [16]Experimental details can be found in the online supporting information

