# Supplementary material for: Enantioselective isothiourea-catalysed α-fluorination of C1-ammonium enolates generated from arylesters
Source: J Fluor Chem. Author manuscript; Available in PMC 2025 Jul 5. (PMC7617865; doi:10.1016/j.jfluchem.2025.110434)
Supplement: SI [file EMS206751-supplement-SI.pdf]

Supplementary Information

Enantioselective Isothiourea-Catalysed α-Fluorination of C1-Ammonium Enolates Generated from Arylesters

Nicole Stanek, Lotte Stockhammer, Anja Moser, Mario Waser

Contents

1. General Information 2
2. Synthesis of FBTM 3
3. Synthesis of PFP Esters 4
4. General Procedure for the α-Fluorination Reactions 9
5. Follow-Up Transformations 15
6. References 17
7. Annex 1: NMR Spectra of Novel Compounds 18
8. Annex 2: HPLC Chromatograms 33

1. General Information

NMR spectra were recorded on a Bruker Avance III 300 MHz spectrometer with a broad band observe probe and a sample changer for 16 samples, a Bruker Avance DRX 500 MHz spectrometer or a Bruker Avance III 700 MHz spectrometer with an Ascend magnet and TCI cryoprobe, which are property to the Austro Czech NMR Research Center "RERI uasb". All NMR spectra were referenced on the solvent residual peak ($CDCl_3$: *δ* 7.26 ppm for ^1H NMR and *δ* 77.16 ppm for ^{13}C NMR. NMR data are reported as follows: chemical shift (δ ppm), multiplicity (s = singlet; d = doublet; t = triplet; q = quartet; m = multiplet; br = broad; app. = apparent), coupling constants (Hz) and integrals. High resolution mass spectra were obtained using an Agilent QTOF 6520 with ESI source. Optical rotations were measured on a Schmidt+Haensch Unipol L 100 polarimeter ($[\alpha]_D$ values are listed in deg·cm^3·g^{-1}·dm^{-1}; concentration c is given in g/100 mL). Preparative column chromatography was carried out using Davisil LC 60A 70– 200 MICRON silica gel. Thin layer chromatography was performed on Macherey-Nagel pre-coated TLC plates (silica gel, 60 F_{254}, 0.20 mm, ALUGRAM® Xtra SIL). TLC plates were visualized under 254 nm UV lamp. In case of the use of deactivated silica, this was prepared by preparing a slurry of 150 g silica in a low boiling solvent (DCM, Et_2O or petrol ether) and adding 5 mL Et_3N. This mixture was put on the rotary evaporator and the solvent was removed. The deactivated silica was further dried under vacuum. Enantiomeric ratios (*er*) were determined by HPLC analysis using a Dionex Summit HPLC system with a CHIRALCEL OJ-H, OD-H (4.6 × 250 mm, 5 µm) or CHIRAL ART Cellulose-SB (4.6 mm × 250 mm, 5 µm) chiral stationary phase. Semipreparative HPLC was carried out using a Thermo Scientific Dionex Ultimate 3000 system with variable wavelength detection and a Grace Alltima Silica 10µm 250x10 mm column. Dry solvents were taken from an mBRAUN SPS solvent purifier. Water refers to deionized water in any case. All chemicals were purchased from commercial suppliers and used without further purification unless otherwise stated.

Isothiourea catalysts BTM (CAS 885051-07-0), HBTM (CAS 1316861-19-4), and *Hyper*BTM (CAS 1203507-02-1) are commercially available and were used as obtained from the provider without further purification. Authentic racemic samples were obtained by using achiral ITU DHPB as catalyst, which was synthesized inhouse as described previously.[1]

As it was observed for our chlorination reactions,[5] ionization of fluorinated esters **5** proofed to be problematic using an ESI source. Hence, (low resolution) EI ionization was performed to confirm product formation of the methyl esters via mass spectrometry. Most fluorinated products are known compounds anyways, with the respective reference data given for the specific entries to the scope (*vide infra*).

2. Synthesis of FBTM

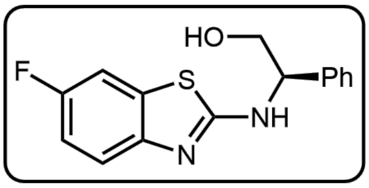

With slight adaptions to literature[1], 2-chloro-6-fluorobenzo[d]thiazole (1.0 eq) and (*R*)-phenyl glycinol (1.1 eq) were suspended in chlorobenzene (0.3 mol L^{-1}). DIPEA (4.0 eq) was added, and the mixture was heated to 140°C for 70 h. After cooling to r.t. the reaction was quenched with water and extracted with EtOAc three times. The combined organic layers were dried with Na_2SO_4, filtered and concentrated on the rotavapor. The crude products were purified by column chromatography on silica (heptanes/EtOAc 5/1 followed by 2/1 and pure EtOAc). On a 5 mmol scale, the product was obtained as a brownish solid in a yield of 43%. The compound is known to literature but has not been characterized before.[2]

^{1}H-NMR (300 MHz, $CDCl_3$, 298 K) δ / ppm = 7.43-7.31 (m, 7 H, Ar-**H**), 7.22 (dd, J_1 = 2.6 Hz, J_2 = 8.1 Hz, 1 H, Ar-**H**), 6.99 (td, J_1 = 2.6 Hz, J_2 = 9.0 Hz, 1 H, Ar-**H**), 4.85 (dd, J_1 = 4.1 Hz, J_2 = 6.4 Hz, 1 H, -C**H**), 4.02-3.91 (m, 2 H, C**H$_2$**); **^{13}C-NMR** (75 MHz, $CDCl_3$, 298 K) δ / ppm = 167.2 (1 C, -N=**C**), 158.5 (d, *J* = 240.8 Hz, 1 C, **C$_{Ar}$**-F), 148.1 (d, *J* = 1.9 Hz, 1 C, **C$_{Ar}$**), 138.5 (1 C, **C$_{Ar}$**), 131.4 (d, *J* = 10.8 Hz, 1 C, **C$_{Ar}$**), 129.1 (2 C, **C$_{Ar}$**), 128.5 (1 C, **C$_{Ar}$**), 127.0 (2 C, **C$_{Ar}$**), 119.5 (d, *J* = 8.8 Hz, 1 C, **C$_{Ar}$**), 113.9 (d, *J* = 23.9 Hz, 1 C, **C$_{Ar}$**), 107.7 (d, *J* = 27.2 Hz, 1 C, **C$_{Ar}$**), 67.9 (1 C, -**C**H), 62.0 (1 C, -**C**H_2); **^{19}F-NMR** (282 MHz, CDCl3, 298 K) δ / ppm = -120.7 (m, 1 F, Ar-**F**).

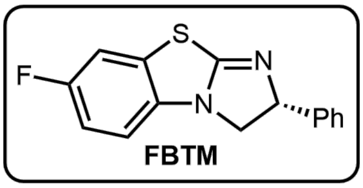

According to literature[1], the product from the previous step (1.0 eq) was dissolved in anhydrous DCM (0.1 mol L^{-1}) and cooled to 0 °C. Et_3N (3.0 eq) and MsCl (1.5 eq) were added dropwise simultaneously. After complete addition, the mixture was stirred another 90 min at r.t. After this, MeOH (1.5 eq) and another portion Et_3N (3.0 eq) were added, and the mixture was heated to reflux overnight. After cooling to r.t., water was added, and it was extracted with DCM three times. The combined organic layers were dried over Na_2SO_4, filtered and concentrated. Recrystallization from EtOAc gave **FBTM** in a yield of 64% as an off-white solid (2.5 mmol scale). The compound is known to literature but has not been characterized before.[2]

Melting point: 116-119 °C; **^{1}H-NMR** (300 MHz, $CDCl_3$, 298 K) δ / ppm = 7.38-7.27 (m, 5 H, Ar-**H**), 7.07 (dd, J_1 = 2.5 Hz, J_2 = 10.1 Hz, 1 H, Ar-**H**), 6.90 (td, J_1 = 2.5 Hz, J_2 = 8.8 Hz, 1 H, Ar-**H**), 6.58 (dd, J_1 = 4.3 Hz, J_2 = 8.6 Hz, 1 H, Ar-**H)**, 5.67 (dd, J_1 = 8.3 Hz, J_2 = 10.1 Hz, 1 H, -C**H**), 4.26 (dd, J_1 = 8.8 Hz, J_2 = 10.1 Hz, 1 H, -C**H$_2$**), 3.69 (t, *J* = 8.5 Hz, 1 H, -C**H$_2$**); **^{13}C-NMR** (75 MHz, $CDCl_3$, 298 K) δ / ppm = 166.8 (1 C, -N=**C**), 158.0 (d, *J* = 240.7 Hz, 1 C, **C$_{Ar}$**-F), 142.8 (1 C, **C$_{Ar}$**), 133.8 (d, *J* = 1.9 Hz, 1 C, **C$_{Ar}$**), 128.9 (2 C, **C$_{Ar}$**), 128.6 (d, *J* = 9.9 Hz, 1 C, **C$_{Ar}$**), 127.8 (1 C, **C$_{Ar}$**), 126.6 (2 C, **C$_{Ar}$**), 113.3 (d, *J* = 23.7 Hz, 1 C, **C$_{Ar}$**), 111.0 (d, *J* = 27.5 Hz, 1 C, **C$_{Ar}$**), 108.6 (d, *J* = 8.4 Hz, 1 C, **C$_{Ar}$**), 75.7 (1 C, -**C**H), 53.0 (1 C, -**C**H_2); **^{19}F-NMR** (282 MHz, $CDCl_3$, 298 K) δ / ppm = -121.1 (m, 1 F, Ar-**F**). **HRMS**: *m/z* Calcd for $C_{15}H_{14}FNO$ $[M+H]^+$ 271.0705; Found 271.0710.

3. Synthesis of PFP Esters

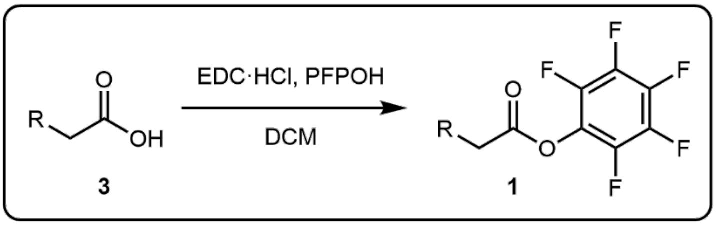

According to a known procedure[3], the acid derivative (1 eq) and EDC·HCl (1.3 eq) were dissolved in anhydrous DCM (0.5 mol L-1) at r.t. and stirred for 10 min. Then, pentafluoro phenol (1.5 eq) was added, and the mixture was stirred overnight at r.t. The reaction was quenched with water and the layers were separated. The aqueous phase was extracted with DCM trice. The combined organic phases were dried with Na_2SO_4, filtrated, and concentrated on the rotary evaporator to yield the crude aryl esters, with were purified by column chromatography using DCM as eluent and deactivated silica as stationary phase.

Perfluorophenyl 2-phenylacetate (1a)

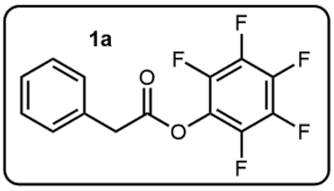

Following the general procedure, perfluorophenyl 2-phenylacetate (**1a**) was obtained as a white solid in a yield of 57% (10 mmol scale). Spectral data was in accordance with literature.[4]

^{1}**H-NMR** (300 MHz, $CDCl_3$, 298 K): δ / ppm = 7.39-7.36 (m, 5 H, Ar-**H**), 3.98 (s, 2 H, -C**H**$_2$)

Perfluorophenyl 2-(*p*-tolyl)acetate (1b)

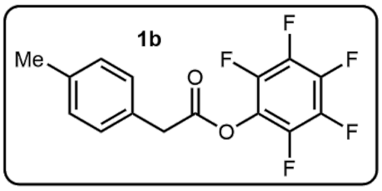

Following the general procedure, perfluorophenyl 2-(*p*-tolyl)acetate (**1b**) was obtained as a colourless liquid in a yield of 58% (10 mmol scale). Spectral data was in accordance with literature.[4]

^{1}**H-NMR** (300 MHz, $CDCl_3$, 298 K): δ / ppm = 7.25 (d, J = 7.9 Hz, 2 H, Ar-**H**), 7.19 (d, J = 7.9 Hz, 2 H, Ar-**H**), 3.93 (s, 2 H, -C**H**$_2$), 2.36 (s, 3 H, -C**H**$_3$).

Perfluorophenyl 2-(*m*-tolyl)acetate (1c)

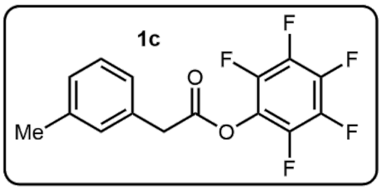

Following the general procedure, perfluorophenyl 2-(*m*-tolyl)acetate (**1c**) was obtained as a colourless liquid in a yield of 74% (10 mmol scale). Spectral data was in accordance with literature.[5]

^{1}H-NMR (300 MHz, $CDCl_3$, 298 K): δ / ppm = 7.30-7.25 (m, 1 H, Ar-**H**), 7.17-7.14 (m, 1 H, Ar-**H**), 3.94 (s, 2 H, -C**H$_2$**), 2.38 (s, 3 H, -C**H$_3$**).

Perfluorophenyl 2-(*o*-tolyl)acetate (1d)

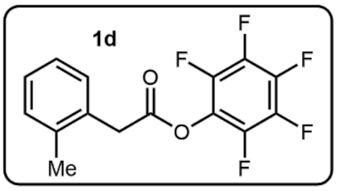

Following the general procedure, perfluorophenyl 2-(*o*-tolyl)acetate (**1d**) was obtained as a colourless liquid in a yield of 59%. Spectral data was in accordance with literature.[4]

^{1}H-NMR (300 MHz, $CDCl_3$, 298 K): δ / ppm = 7.29-7.21 (m, 4 H, Ar-**H**), 3.99 (s, 2 H, -C**H$_2$**), 2.38 (s, 3 H, -C**H$_3$**).

Perfluorophenyl 2-(4-methoxyphenyl)acetate (1e)

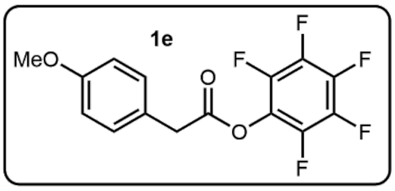

Following the general procedure, perfluorophenyl 2-(4-methoxyphenyl)acetate (**1e**) was obtained as a white solid in a yield of 62% (10 mmol scale). Spectral data was in accordance with literature.[4]

^{1}H-NMR (300 MHz, $CDCl_3$, 298 K): δ / ppm = 7.27 (d, J = 9.0 Hz, 2 H, Ar-**H**), 6.91 (d, J = 9.0 Hz, 2 H, Ar-**H**), 3.91 (s, 2 H, -C**H$_2$**), 3.82 (s, 3 H, -OC**H$_3$**).

Perfluorophenyl 2-(4-(trifluoromethyl)phenyl)acetate (1f)

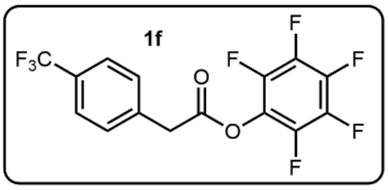

Following the general procedure, perfluorophenyl 2-(4-(trifluoromethyl)phenyl)acetate (**1f**) was obtained as a white solid in a yield of 62% (10 mmol scale). Spectral data was in accordance with literature.[6]

^{1}H-NMR (300 MHz, $CDCl_3$, 298 K): δ / ppm = 7.65 (d, J = 8.1Hz, 2 H, Ar-**H**), 7.49 (d, J = 8.1 Hz, 2 H, Ar-**H**), 4.04 (s, 2 H, -C**H$_2$**).

Perfluorophenyl 2-(4-fluorophenyl)acetate (1g)

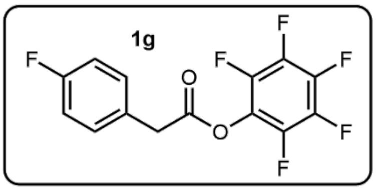

Following the general procedure, perfluorophenyl 2-(4-fluorophenyl)acetate (**1g**) was obtained as a colourless oil in a yield of 69% (10 mmol scale). Spectral data was in accordance with literature.[7]

^{1}H-NMR (300 MHz, $CDCl_3$, 298 K): δ / ppm = 7.33 (dd, J_1 = 5.3 Hz, J_2 = 8.7 Hz, 2 H, Ar-**H**), 7.07 (t, J = 8.7 Hz, 2 H, Ar-**H**), 3.95 (s, 2 H, -C**H$_2$**).

Perfluorophenyl 2-(4-chlorophenyl)acetate (1h)

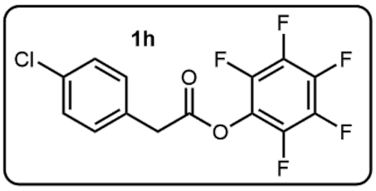

Following the general procedure, perfluorophenyl 2-(4-chlorophenyl)acetate (**1h**) was obtained as a colourless liquid in a yield of 57%. Spectral data was in accordance with literature.[4]

^{1}H-NMR (300 MHz, $CDCl_3$, 298 K): δ / ppm = 7.36 (d, J = 8.6 Hz, 2 H, Ar-**H**), 7.29 (d, J = 8.6 Hz, 2 H, Ar-**H**), 3.94 (s, 2 H, -C**H$_2$**).

Perfluorophenyl 2-(4-bromophenyl)acetate (1i)

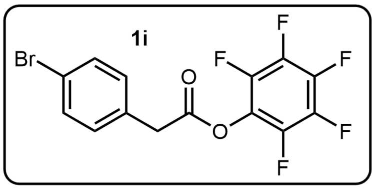

Following the general procedure, perfluorophenyl 2-(4-bromophenyl)acetate (**1i**) was obtained as a colourless liquid in a yield of 63% (10 mmol scale). Spectral data was in accordance with literature.[8]

^{1}H-NMR (300 MHz, $CDCl_3$, 298 K): δ / ppm = 7.51 (d, J = 8.0 Hz, 2 H, Ar-**H**), 7.24 (d, J = 8.0 Hz, 2 H, Ar-**H**), 3.93 (s, 2 H, -C**H$_2$**).

Perfluorophenyl 2-(3-iodophenyl)acetate (1j)

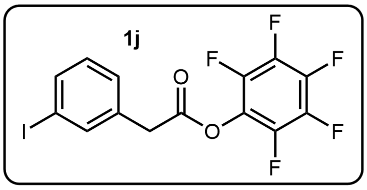

Following the general procedure, perfluorophenyl 2-(3-iodophenyl)acetate (**1j**) was obtained as a white solid in a yield of 62% (10 mmol scale).

^{1}H-NMR (300 MHz, $CDCl_3$, 298 K): δ / ppm = 7.72-7.66 (m, 2 H, Ar-**H**), 7.33 (d, J = 7.8 Hz, 1 H, Ar-**H**), 7.12 (t, J = 7.8 Hz, 1 H, Ar-**H**), 3.91 (s, 2 H, -C**H$_2$**). **^{13}C-NMR** (75 MHz, $CDCl_3$, 298 K) δ / ppm = 166.9 (s, 1 C, -**C**=O), 138.3 (s, 2 C, **C$_{Ar}$**), 137.0 (s, 3 C, **C$_{Ar}$**), 134.1 (s, 1 C, **C$_{Ar}$**), 130.6 (s, 3 C, **C$_{Ar}$**), 128.5 (s, 2 C, **C$_{Ar}$**), 94.6 (s, 1 C, -**C$_{Ar}$**), 39.5 (s, 1 C, -**C**H_2); **^{19}F-NMR** (282 MHz, $CDCl_3$, 298 K) δ / ppm = -152.4 – -152.6 (m, 2 **F**), -157.5 (t, J = 22.1 Hz, 1 **F**), -162.0 – -162.2 (m, 2 **F**).

Perfluorophenyl 2-(naphthalen-2-yl)acetate (1k)

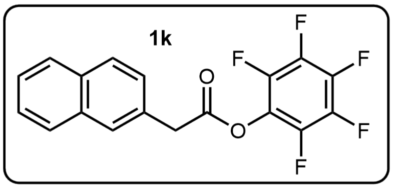

Following the general procedure, perfluorophenyl 2-(naphthalen-2-yl)acetate (**1k**) was obtained as a white solid in a yield of 63% (10 mmol scale). Spectral data was in accordance with literature.[6]

^{1}H-NMR (300 MHz, $CDCl_3$, 298 K): δ / ppm = 7.88-7.82 (m, 4 H, Ar-**H**), 7.52-7.45 (m, 3 H, Ar-**H**), 4.13 (s, 2 H, -C**H$_2$**).

Perfluorophenyl 2-(thiophen-3-yl)acetate (1l)

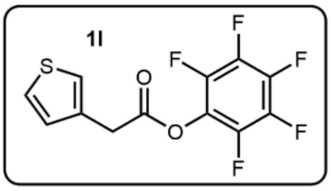

Following the general procedure, perfluorophenyl 2-(thiophen-3-yl)acetate (**1l**) was obtained as a colourless oil in a yield of 57% (10 mmol scale). Spectral data was in accordance with literature.[9]

^{1}H-NMR (300 MHz, $CDCl_3$, 298 K): δ / ppm = 7.37 (dd, J_1 = 3.0 Hz, J_2 = 4.9 Hz, 1 H, Ar-**H**), 7.30-7.27 (m, 1 H, Ar-**H**), 7.13 (dd, J_1 = 1.2 Hz, J_2 = 4.9 Hz, 1 H, Ar-**H**), 4.04 (s, 2 H, -C**H$_2$**).

Perfluorophenyl (*E*)-4-phenylbut-3-enoate (1m)

The compound was obtained in 2 steps from cinnamic alcohol via a carbon monoxide (generated *in situ* from acetic anhydride and formic acid) insertion followed by the standard conditions for PFP ester synthesis.

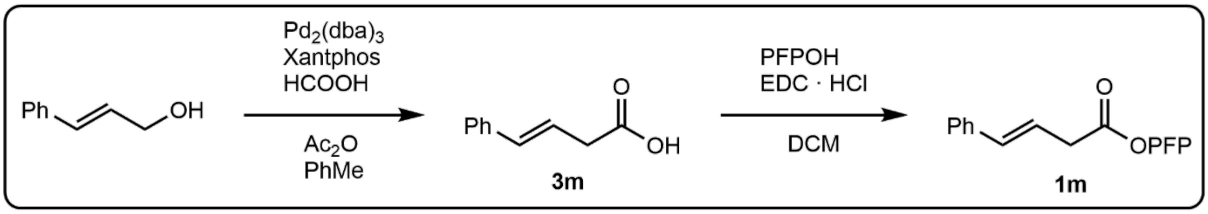

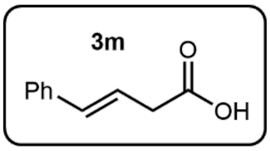

In accordance with literature[10] a pressure resistant Schlenk tube containing a stirrer bar was flame dried under vacuum. Then, $Pd_2(dba)_3$ (31 mg, 0.03 mmol) and Xantphos (80 mg, 0.13 mmol) were added into the flask which then was evacuated and backfilled with argon three times. Then, the catalyst and ligand were dissolved in 15 mL anhydrous toluene. Cinnamic alcohol (0.88 g, 6.6 mmol), acetic anhydride (2.0 mL, 20 mmol) and formic acid (0.75 mL, 20 mmol) were added. The flask was sealed and heated to 80 °C for 24 h. After cooling to r.t., the mixture was diluted with EtOAc and then concentrated on the rotary evaporator. The crude product was purified by column chromatography on silica (heptanes/EtOAc gradient form 10/1 to 1/1) to give acid **3m** as a slightly yellow solid in a yield of 50%. Spectral data was in accordance with literature.[10]

^{1}**H-NMR** (300 MHz, $CDCl_3$, 298 K): δ / ppm = 7.40-7.29 (m, 4 H, Ar-**H**), 7.26-7.24 (m, 1 H, Ar-**H**), 6.53 (app. d, J = 15.9 Hz, 1 H, -C**H**), 6.29 (dt, J_1 = 7.1 Hz, J_2 = 15.9 Hz, 1 H, -C**H**), 3.30 (dd, J_1 = 1.2 Hz, J_2 = 7.1 Hz, 2 H, -C**H₂**).

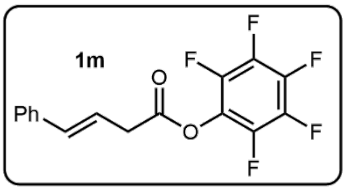

Following the general procedure, perfluorophenyl (E)-4-phenylbut-3-enoate (**1m**) was obtained as a yellow solid in a yield of 61% (3 mmol scale). Spectral data was in accordance with literature.[11]

^{1}**H-NMR** (300 MHz, $CDCl_3$, 298 K): δ / ppm = 7.42-7.29 (m, 5 H, Ar-**H**), 6.64 (app. d, J = 15.9 Hz, 1 H, -C**H**), 6.33 (dt, J_1 = 7.0 Hz, J_2 = 15.9 Hz, 1 H, -C**H**), 3.60 (dd, J_1 = 1.4 Hz, J_2 = 7.0Hz, 2 H, -C**H₂**).

4. General Procedure for the α-Fluorination Reactions

General procedure for the α-fluorination of aryl esters 1 followed by an immediate alcohol quench

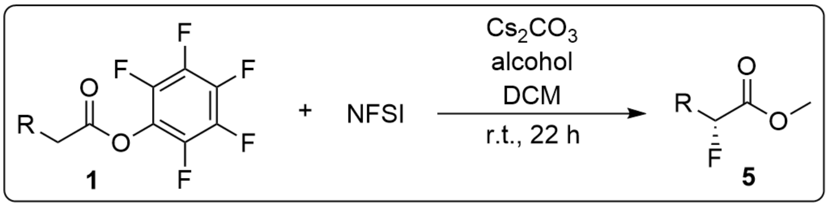

The respective *pfp*-ester **1** (0.1 mmol), Cs_2CO_3 (97.7 mg, 0.3 mmol), NFSI (47.3 mg, 0.15 mmol) and 8-fluoro benzotetramisole (**F-BTM**, 2.7 mg, 10mol%) were dissolved in 1 mL DCM in a Schlenk flask. The respective alcohol (MeOH, benzyl alcohol, diphenyl methanol, 0.2 mmol) was added and the mixture was stirred at r.t. for 20 h. The mixture was filtered over a silica plug, washed with DCM and carefully evaporated to dryness *in vacuo* using an ice-water bath. The crude products were purified by semi-preparative column chromatography (hexane/EtOAc).

NOTE: As stated in the main manuscript, products **5** were found to be rather volatile. Thus, isolated yields after column chromatography and standard solvent evaporation on a rotavapor were often considerably lower than the reaction yields determined by NMR, i.e. on these smaller reaction scales. In principle, a careful distillation procedure using a Vigreux column as well helps reducing these losses. However, in most cases we just carried out a "standard" isolation, to obtain material of sufficient purity, thus rationalizing the sometimes significant deviations between isolated and NMR yields.

Characterization data for α-fluorination methyl esters

Methyl 2-fluoro-2-phenylacetate 5a

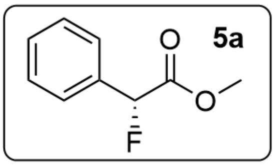

Fluorinated ester *(R)*-**5a** (*er* 99:1) was obtained as a colourless oil in a isolated yield of 57.3 mg (34%) via the standard procedure and with an initial NMR yield of 78% using 1.0 mmol starting material. Analytical data was in accordance with literature.[12] **TLC** (Toluene/EtOAc 20/1): R_f= 0.53 (UV), $[\alpha]_D^{20}$ = -59.2 (*c* 0.47, $CHCl_3$, 99:1 *e.r.*); **^{1}H-NMR** (300 MHz, $CDCl_3$, 298 K) δ / ppm = 7.47-7.40 (m, 5 H, Ar-**H**), 5.80 (d, J = 47.6 Hz, 1 H, -C**H**F), 3.79 (s, 3 H, -C**H$_3$**); **^{13}C-NMR** (75 MHz, $CDCl_3$, 298 K) δ / ppm = 169.7 (d, J = 27.7 Hz, 1 C, -**C**=O), 134.3 (d, J = 20.5 Hz, 1 C, **C$_{Ar}$**), 129.8 (d, J = 2.2 Hz, 1 C, **C$_{Ar}$**), 128.9 (s, 2 C, **C$_{Ar}$**), 126.8 (d, J = 6.1 Hz, 2 C, **C$_{Ar}$**), 89.5 (d, J = 185.5 Hz, 1 C, -**C**F), 52.8 (s, 1 C, -O**C**H$_3$); **^{19}F-NMR** (282 MHz, $CDCl_3$, 298 K) δ / ppm = -179.8 (d, J = 47.6 Hz, 1 F) ; **EI-MS**: *m/z* Calcd for $C_9H_9FO_2$ $[M]^{+\cdot}$ 168; Found 168, 109 ($[M-COOMe]^+$). **HPLC** (CHIRALCEL OJ-H, *n*-hexane/IPA = 7/3, flow rate = 1.0 mL min^{-1}, I = 220 nm): t_r = 10.2 min *(major)*, 12.3 min *(minor)*.

Methyl 2-fluoro-2-(*p*-tolyl)acetate 5b

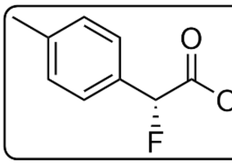
5b

Fluorinated ester *(R)*-**5b** (*er* 99:1) was obtained as a colourless oil in an isolated yield of 5.3 mg (27%) and an initial NMR yield of 56% via the standard procedure. Analytical data was in accordance with literature.[13] **TLC** (Toluene/EtOAc 20/1): R_f = 0.52 (UV), $[\alpha]_D^{20}$ = -145.8 (*c* 0.32, $CHCl_3$, 99:1 *e.r.*); **^{1}H-NMR** (300 MHz, $CDCl_3$, 298 K) δ / ppm = 7.36-7.33 (m, 2 H, Ar-**H**), 7.22 (d, *J* = 8.14 Hz, 2 H, Ar-**H**), 5.76 (d, *J* = 47.7 Hz, 1 H, -C**H**F), 3.78 (s, 3 H, -C**H$_3$**), 2.37 (d, *J* = 1.31 Hz, 3 H, Ar-C**H$_3$**); **^{13}C-NMR** (75 MHz, $CDCl_3$, 298 K) δ / ppm = 169.4 (d, *J* = 28.2 Hz, 1 C, -**C**=O), 139.9 (d, *J* = 2.5 Hz, 1 C, **C$_{Ar}$**), 131.3 (d, *J* = 20.7 Hz, 1 C, **C$_{Ar}$**), 129.6 (s, 2 C, **C$_{Ar}$**), 126.9 (d, *J* = 5.6 Hz, 2 C, **C$_{Ar}$**), 89.4 (d, *J* = 184.7 Hz, 1 C, -**C**F), 52.7 (s, 1 C, -O**C**H$_3$), 21.4 (s, 1 C, C$_{Ar}$-**C**H$_3$); **^{19}F-NMR** (282 MHz, $CDCl_3$, 298 K) δ / ppm = -177.9 (d, *J* = 47.7 Hz, 1 F) ; **EI-MS**: *m/z* Calcd for $C_{10}H_{11}FO_2$ $[M]^{+\cdot}$ 182; Found 182, 123 ($[M – COOMe]^+$). **HPLC** (CHIRALCEL OJ-H, *n*-hexane/IPA = 7/3, flow rate = 1.0 mL min^{-1}, *I* = 220 nm): t_r = 9.8 min *(major)*, 12.8 min *(minor).*

Methyl 2-fluoro-2-(*m*-tolyl)acetate 5c

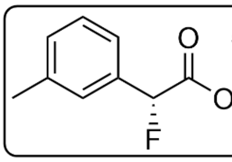
5c

Fluorinated ester *(R)*-**5c** (*er* 99:1) was obtained as a colourless oil in an isolated yield of 5.6 mg (30%) and an initial NMR yield of 65% via the standard procedure. Analytical data was in accordance with literature.[13] **TLC** (Toluene/EtOAc 20/1): R_f = 0.54 (UV), $[\alpha]_D^{20}$ = -91.8 (*c* 0.51, $CHCl_3$, 99:1 *e.r.*); **^{1}H-NMR** (300 MHz, $CDCl_3$, 298 K) δ / ppm = 7.37-7.20 (m, 4 H, Ar-**H**), 5.75 (d, *J* = 47.7 Hz, 1 H, -C**H**F), 3.78 (s, 3 H, -C**H$_3$**), 2.38 (s, 3 H, Ar-C**H$_3$**); **^{13}C-NMR** (75 MHz, $CDCl_3$, 298 K) δ / ppm = 169.3 (d, *J* = 27.8 Hz, 1 C, -**C**=O), 138.8 (s, 1 C, **C$_{Ar}$**), 134.2 (d, *J* = 20.2 Hz, 1 C, **C$_{Ar}$**), 130.6 (s, 1 C, **C$_{Ar}$**), 128.8 (s, 1 C, **C$_{Ar}$**), 127.4 (d, *J* = 5.7 Hz, 1 C, **C$_{Ar}$**), 124.0 (d, *J* = 6.0 Hz, 1 C, **C$_{Ar}$**), 89.7 (d, *J* = 185.3 Hz, 1 C, -**C**F), 52.8 (s, 1 C, -O**C**H$_3$), 21.5 (s, 1 C, C$_{Ar}$-**C**H$_3$); **^{19}F-NMR** (282 MHz, $CDCl_3$, 298 K) δ / ppm = -179.2 (d, *J* = 47.7 Hz, 1 F) ; **EI-MS**: *m/z* Calcd for $C_{10}H_{11}FO_2$ $[M]^{+\cdot}$ 182; Found 182, 123 ($[M – COOMe]^+$). **HPLC** (CHIRALCEL OJ-H, *n*-hexane/IPA = 7/3, flow rate = 1.0 mL min^{-1}, *I* = 220 nm): t_r = 8.2 min *(major)*, 10.6 min *(minor).*

Methyl 2-fluoro-2-(*o*-tolyl)acetate 5d

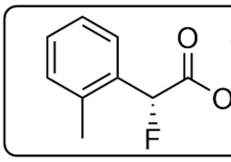
5d

Fluorinated ester *(R)*-**5d** (*er* 64:36) was obtained as a colourless oil in an isolated yield of 5.7 mg (30%) and an initial NMR yield of 40% via the standard procedure. Analytical data was in accordance with literature.[13] **TLC** (Toluene/EtOAc 20/1): R_f = 0.50 (UV), $[\alpha]_D^{20}$ = -118.5 (*c* 0.37, $CHCl_3$, 99:1 *e.r.*); **^{1}H-NMR** (300 MHz, $CDCl_3$, 298 K) δ / ppm = 7.42-7.25 (m, 4 H, Ar-**H**), 6.01 (d, *J* = 47.2 Hz, 1 H, -C**H**F), 3.81 (s, 3 H, -C**H$_3$**), 2.46 (s, 3 H, Ar-C**H$_3$**); **^{19}F-NMR** (282 MHz, $CDCl_3$, 298 K) δ / ppm = -179.4 (d, *J* = 47.0 Hz, 1 F) ; **EI-MS**: *m/z* Calcd for

$C_{10}H_{11}FO_2$ $[M]^{+\cdot}$ 182; Found 182, 123 ($[M – COOMe]^+$). **HPLC** (CHIRAL ART Cellulose-SB, *n*-hexane/IPA = 100/1, flow rate = 0.5 mL min^{-1}, *I* = 220 nm): t_r = 20.6 min *(minor)*, 22.1 min *(major)*.

Methyl 2-fluoro-2-(4-methoxyphenyl)acetate 5e

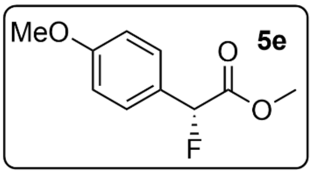

Fluorinated ester *(R)*-**5e** (*er* 99.5:0.5) was obtained as a colourless oil in an isolated yield of 2.3 mg (12%) and an initial NMR yield of 23% via the standard procedure. Analytical data was in accordance with literature.[14] **TLC** (Toluene/EtOAc 20/1): R_f = 0.45 (UV), $[\alpha]_D^{20}$ = -32.9 (*c* 0.22, CH_2Cl_2, 99.5:0.5 *e.r.*); **^{1}H-NMR** (300 MHz, $CDCl_3$, 298 K) δ / ppm = 7.40-7.37 (m, 2 H, Ar-**H**), 6.93 (d, *J* = 8.2 Hz, 2 H, Ar-**H**), 5.73 (d, *J* = 47.7 Hz, 1 H, -C**H**F), 3.82 (s, 3 H, -OC**H$_3$**), 3.78 (s, 3 H, -C**H$_3$**); **^{19}F-NMR** (282 MHz, $CDCl_3$, 298 K) δ / ppm = -174.8 (d, *J* = 47.8 Hz, 1 F) ; **EI-MS**: *m/z* Calcd for $C_{10}H_{11}FO_3$ $[M]^{+\cdot}$ 198; Found 198, 139 ($[M – COOMe]^+$). **HPLC** (CHIRALCEL OJ-H, *n*-hexane/IPA = 7/3, flow rate = 1.0 mL min^{-1}, *I* = 220 nm): t_r = 19.4 min *(major)*, 22.4 min *(minor)*.

Methyl 2-fluoro-2-(4-(trifluoromethyl)phenyl)acetate 5f

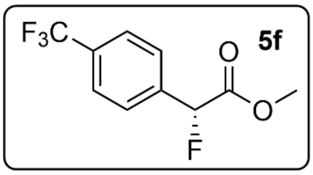

Fluorinated ester *(R)*-**5f** (*er* 75:25) was obtained as a colourless oil in an isolated yield of 7.3 mg (29%) and an initial NMR yield of 92% via the standard procedure. Analytical data was in accordance with literature.[14] **TLC** (Toluene/EtOAc 20/1): R_f = 0.49 (UV), $[\alpha]_D^{20}$ = -71.5 (*c* 0.31, $CHCl_3$, 75:25 *e.r.*); **^{1}H-NMR** (700 MHz, $CDCl_3$, 298 K) δ / ppm = 7.64 (dd, J_1 = 53.6 Hz, J_2 = 7.9 Hz, 4 H, Ar-**H**), 5.87 (d, *J* = 47.3 Hz, 1 H, -C**H**F), 3.80 (s, 3 H, -C**H$_3$**); **^{13}C-NMR** (176 MHz, $CDCl_3$, 298 K) δ / ppm = 168.3 (d, *J* = 27.0 Hz, 1 C, -**C**=O), 138.0 (d, *J* = 20.9 Hz, 1 C, $\mathbf{C_{Ar}}$), 131.9 (d, *J* = 31.3 Hz, 1 C, $\mathbf{C_{Ar}}$), 126.8 (d, *J* = 6.7 Hz, 2 C, $\mathbf{C_{Ar}}$), 125.9 (q, *J* = 3.7 Hz, 2 C, $\mathbf{C_{Ar}}$), 123.9 (d, *J* = 272.4 Hz, 1 C, **C**F_3), 88.7 (d, *J* = 187.7 Hz, 1 C, -**C**F), 53.0 (s, 1 C, -O**C**H_3); **^{19}F-NMR** (282 MHz, $CDCl_3$, 298 K) δ / ppm = -62.9 (s, 3 F), -184.3 (d, *J* = 47.3 Hz, 1 F); **EI-MS**: *m/z* Calcd for $C_{10}H_8F_4O_2$ $[M]^{+\cdot}$ 236; Found 236, 177 ($[M – COOMe]^+$). **HPLC** (CHIRALCEL OJ-H, *n*-hexane/IPA = 7/3, flow rate = 1.0 mL min^{-1}, *I* = 220 nm): t_r = 6.2 min *(major)*, 7.2 min *(minor)*.

Methyl 2-fluoro-2-(4-fluorophenyl)acetate 5g

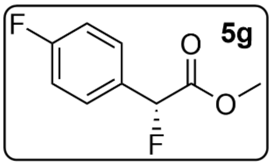

Fluorinated ester *(R)*-**5g** (*er* 99:1) was obtained as a colourless oil in an isolated yield of 6.8 mg (27%) and an initial NMR yield of 62% via the standard procedure. Analytical data was in accordance with literature.[14] **TLC** (Toluene/EtOAc 20/1): R_f = 0.48 (UV), $[\alpha]_D^{20}$ = -132.6 (*c* 0.46, $CHCl_3$, 99:1 *e.r.*); **^{1}H-NMR** (300 MHz, $CDCl_3$, 298 K) δ / ppm = 7.48-7.43 (m, 2 H, Ar-**H**), 7.10 (t, *J* = 5.8 Hz, 2 H, Ar-**H**), 5.77 (d, *J* = 47.4 Hz, 1 H, -C**H**F), 3.79 (s, 3 H, -C**H$_3$**); **^{13}C-NMR** (75 MHz, $CDCl_3$, 298 K) δ / ppm = 169.0 (d, *J* = 28.1 Hz, 1 C, -**C**=O), 163.6 (dd, J_1 =

248.7 Hz, J_2 = 2.6 Hz, 1 C, **C**$_{\mathbf{Ar}}$-F), 130.2 (dd, J_1 = 20.5 Hz, J_2 = 3.4 Hz, 1 C, **C**$_{\mathbf{Ar}}$), 128.8 (dd, J_1 = 9.3 Hz, J_2 = 5.9 Hz, 2 C, **C**$_{\mathbf{Ar}}$), 116.2 (d, J = 22.0 Hz, 2 C, **C**$_{\mathbf{Ar}}$), 88.7 (d, J = 186.0 Hz, 1 C, -**C**F), 52.8 (s, 1 C, -O**C**H$_3$); **^{19}F-NMR** (282 MHz, CDCl$_3$, 298 K) δ / ppm = -111.1 – -111-3 (m, 1 F), -178.5 (d, J = 48.1 Hz, 1 F); **EI-MS**: *m/z* Calcd for $C_9H_8F_2O_2$ $[M]^{+\cdot}$ 186; Found 186, 127 ($[M – COOMe]^+$). **HPLC** (CHIRALCEL OJ-H, *n*-hexane/IPA = 7/3, flow rate = 1.0 mL min^{-1}, *I* = 220 nm): t_r = 8.5 min *(major)*, 10.9 min *(minor).*

Methyl 2-fluoro-2-(4-bromophenyl)acetate 5h

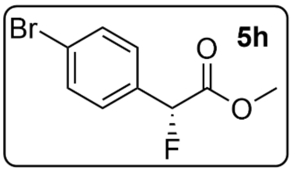

Fluorinated ester *(R)*-**5h** (*er* 97:3) was obtained as a colourless oil in a yield of 17.8 mg (66%) and an initial NMR yield of 87% via the standard procedure. Analytical data was in accordance with literature.[13] **TLC** (Toluene/EtOAc 20/1): R_f= 0.67 (UV), $[\alpha]_D^{20}$ = -74.1 (*c* 1.30, CHCl$_3$, 97:3 *e.r.*); **^{1}H-NMR** (300 MHz, CDCl$_3$, 298 K) δ / ppm = 7.56-7.33 (m, 4 H, Ar-**H**), 5.75 (d, J = 47.3 Hz, 1 H, -C**H**F), 3.78 (s, 3 H, -C**H**$_3$); **^{13}C-NMR** (75 MHz, CDCl$_3$, 298 K) δ / ppm = 168.5 (d, J = 27.5 Hz, 1 C, -**C**=O), 133.1 (d, J = 20.9 Hz, 1 C, **C**$_{\mathbf{Ar}}$-F), 132.0 (s, 2 C, **C**$_{\mathbf{Ar}}$), 128.2 (d, J = 6.2 Hz, 2 C, **C**$_{\mathbf{Ar}}$), 123.9 (d, J = 2.7 Hz, 1 C, **C**$_{\mathbf{Ar}}$), 88.7 (d, J = 186.8 Hz, 1 C, -**C**F), 52.8 (s, 1 C, -O**C**H$_3$); **^{19}F-NMR** (282 MHz, CDCl$_3$, 298 K) δ / ppm = -181.4 (d, J = 47.5 Hz, 1 F); **EI-MS**: *m/z* Calcd for $C_9H_8BrFO_2$ $[M]^{+\cdot}$ 245; Found 245, 187 ($[M – COOMe]^+$). **HPLC** (CHIRALCEL OJ-H, *n*-hexane/IPA = 7/3, flow rate = 1.0 mL min^{-1}, *I* = 220 nm): t_r = 9.3 min *(major)*, 10.9 min *(minor).*

Methyl 2-fluoro-2-(4-chlorophenyl)acetate 5i

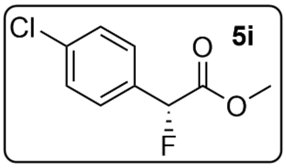

Fluorinated ester *(R)*-**5i** (*er* 96:4) was obtained as a colourless oil in an isolated yield of 10.5 mg (52%) and an initial NMR yield of 83% via the standard procedure. Analytical data was in accordance with literature.[14] **TLC** (Toluene/EtOAc 20/1): R_f= 0.63 (UV), $[\alpha]_D^{20}$ = -93.3 (*c* 0.7, CHCl$_3$, 96:4 *e.r.*); **^{1}H-NMR** (500 MHz, CDCl$_3$, 298 K) δ / ppm = 7.42-7.7.38 (m, 4 H, Ar-**H**), 5.77 (d, J = 47.3 Hz, 1 H, -C**H**F), 3.79 (s, 3 H, -C**H**$_3$); **^{19}F-NMR** (282 MHz, CDCl$_3$, 298 K) δ / ppm = -180.9 (d, J = 47.7 Hz, 1 F); **EI-MS**: *m/z* Calcd for $C_9H_8ClFO_2$ $[M]^{+\cdot}$ 202; Found 202, 143 ($[M – COOMe]^+$). **HPLC** (CHIRALCEL OJ-H, *n*-hexane/IPA = 7/3, flow rate = 1.0 mL min^{-1}, *I* = 220 nm): t_r = 8.5 min *(major)*, 9.9 min *(minor).*

Methyl 2-fluoro-2-(3-iodophenyl)acetate 5j

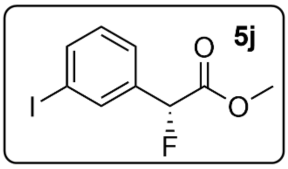

Fluorinated ester *(R)*-**5j** (*er* 95:5) was obtained as a colourless oil in an isolated yield of 18.4 mg (61%) and an initial NMR yield of 98% via the standard procedure. **TLC** (Toluene/EtOAc 20/1): R_f= 0.58 (UV), $[\alpha]_D^{20}$ = -56.1 (*c* 1.1, CHCl$_3$, 95:5 *e.r.*); **^{1}H-NMR** (500 MHz, CDCl$_3$, 298 K) δ / ppm = 7.81 (s, 1 H, Ar-**H**), 7.59 (dd, J_1 = 92.2 Hz, J_2 = 8.2, 2 H, Ar-**H**), 7.14 (t, J = 7.8 Hz, 1 H, Ar-**H**), 5.73 (d, J = 47.3 Hz, 1 H, -C**H**F), 3.79 (s, 3 H, -C**H**$_3$); **^{13}C-NMR** (125.7

MHz, $CDCl_3$, 298 K) δ / ppm = 168.4 (d, J = 27.4 Hz, 1 C, -**C**=O), 138.7 (d, J = 2.0 Hz, 1 C, **C_{Ar}**), 136.2 (d, J = 20.8 Hz, 1 C, **C_{Ar}**), 135.4 (d, J = 6.6 Hz, 1 C, **C_{Ar}**), 130.5 (s, 1 C, **C_{Ar}**), 125.7 (d, J = 6.3 Hz, 1 C, **C_{Ar}**), 94.3 (s, 1 C, **C_{Ar}**), 88.3 (d, J = 187.5 Hz, 1 C, -**C**F), 52.9 (s, 1 C, -O**C**H_3); **^{19}F-NMR** (470 MHz, $CDCl_3$, 298 K) δ / ppm = -182.0 (d, J = 47.2 Hz, 1 F); **EI-MS**: *m/z* Calcd for $C_9H_8FIO_2$ $[M]^{+\cdot}$ 293; Found 293, 236 ($[M - COOMe]^+$). **HPLC** (CHIRALCEL OJ-H, *n*-hexane/IPA = 7/3, flow rate = 1.0 mL min^{-1}, I = 220 nm): t_r = 10.9 min *(major)*, 12.2 min *(minor)*.

Methyl 2-fluoro-2-(naphthalen-2-yl)acetate 5k

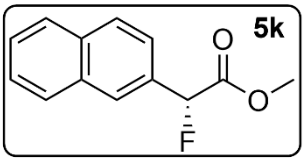

Fluorinated ester *(R)*-**5k** (*er* 98:2) was obtained as a colourless oil in an isolated yield of 15.5 mg (69%) and an initial NMR yield of 75% via the standard procedure. Analytical data was in accordance with literature.[15] **TLC** (Toluene/EtOAc 20/1): R_f = 0.50 (UV), $[\alpha]_D^{20}$ = -105.1 (*c* 0.69, $CHCl_3$, 98:2 *e.r.*); **^{1}H-NMR** (700 MHz, $CDCl_3$, 298 K) δ / ppm = 7.95 (s, 1 H, Ar-**H**), 7.89-7.53 (m, 6 H, Ar-**H**), 5.96 (d, J = 47.5 Hz, 1 H, -C**H**F), 3.79 (s, 3 H, -C**H_3**); **^{13}C-NMR** (175 MHz, $CDCl_3$, 298 K) δ / ppm = 169.0 (d, J = 27.9 Hz, 1 C, -**C**=O), 133.8 (s, 1 C, **C_{Ar}**), 132.9 (s, 1 C, **C_{Ar}**), 131.5 (d, J = 20.4 Hz, 1 C, **C_{Ar}**), 128.8 (s, 1 C, **C_{Ar}**), 128.3 (s, 1 C, **C_{Ar}**), 127.8 (s, 1 C, **C_{Ar}**), 126.7 (s, 1 C, **C_{Ar}**), 126.6 (d, J = 7.3 Hz, 1 C, **C_{Ar}**), 123.5 (d, J = 5.0, 1 C, **C_{Ar}**), 89.6 (d, J = 185.8 Hz, 1 C, -**C**F), 52.7 (s, 1 C, -O**C**H_3); **^{19}F-NMR** (282 MHz, $CDCl_3$, 298 K) δ / ppm = -179.5 (d, J = 47.5 Hz, 1 F); **EI-MS**: *m/z* Calcd for $C_{13}H_{11}FO_2$ $[M]^{+\cdot}$ 218; Found 218, 159 ($[M - COOMe]^+$). **HPLC** (CHIRALCEL OJ-H, *n*-hexane/IPA = 7/3, flow rate = 1.0 mL min^{-1}, I = 272 nm): t_r = 18.0 min *(major)*, 21.7 min *(minor)*.

Methyl 2-fluoro-2-(thiophen-3-yl)acetate 5l

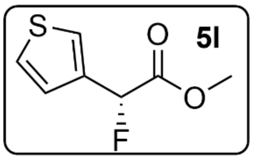

Fluorinated ester *(R)*-**5l** (*er* 97:3) was obtained as a colourless oil in an isolated yield of 5.4 mg (32%) and an initial NMR yield of 76% via the standard procedure. **TLC** (Toluene/EtOAc 20/1): R_f = 0.40 (UV), $[\alpha]_D^{20}$ = -81.6 (*c* 1.1, $CHCl_3$, 97:3 *e.r.*); **^{1}H-NMR** (700 MHz, $CDCl_3$, 298 K) δ / ppm = 7.47-7.46 (m, 1 H, Ar-**H**), 7.37-7.35 (m, 1 H, Ar-**H**), 7.17 (d, J = 5.1 Hz, 1 H, Ar-**H**), 5.90 (d, J = 48.0 Hz, 1 H, -C**H**F), 3.81 (s, 3 H, -C**H_3**); **^{13}C-NMR** (175 MHz, $CDCl_3$, 298 K) δ / ppm = 168.8 (d, J = 27.2 Hz, 1 C, -**C**=O), 134.8 (d, J = 22.0 Hz, 1 C, **C_{Ar}**), 127.1 (s, 1 C, **C_{Ar}**), 125.8 (d, J = 3.5 Hz, 1 C, **C_{Ar}**), 125.1 (d, J = 7.7 Hz, 1 C, **C_{Ar}**), 85.7 (d, J = 184.6 Hz, 1 C, -**C**F), 52.9 (s, 1 C, -OCH_3); **^{19}F-NMR** (282 MHz, $CDCl_3$, 298 K) δ / ppm = -176.3 (d, J = 48.0 Hz, 1 F); **EI-MS**: *m/z* Calcd for $C_7H_7FO_2S$ $[M]^{+\cdot}$ 174; Found 174, 115 ($[M - COOMe]^+$). **HPLC** (CHIRALCEL OJ-H, *n*-hexane/IPA = 7/3, flow rate = 1.0 mL min^{-1}, I = 220 nm): t_r = 12.7 min *(major)*, 15.0 min *(minor)*.

Methyl 2-fluoro-4-phenylbut-3-enoate 5m

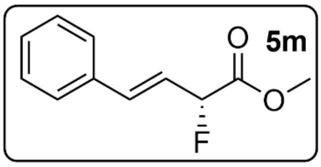

Fluorinated ester *(R)*-**5m** (*er* 70:30) was obtained as a colourless oil in an isolated yield of 7.6 mg (39%) and an initial NMR yield of 56% via the standard procedure. Analytical data was in accordance with literature.[16] **TLC** (Toluene/EtOAc 20/1): R_f= 0.44 (UV), $[\alpha]_D^{20}$ = -30.0 (*c* 0.55, $CHCl_3$, 70:30 *e.r.*); **^{1}H-NMR** (700 MHz, $CDCl_3$, 298 K) δ / ppm = 7.43-7.41 (m, 2 H, Ar-**H**), 7.37-7.29 (m, 3 H, Ar-**H**), 6.85 (dd, J_1 = 16.4 Hz, J_2 = 3.3 Hz, 1 H, =C**H**), 6.33-6.25 (m, 1 H, =C**H**), 5.47 (ddd, J_1 = 47.8 Hz, J_2 = 6.7 Hz, J_3 = 1.4 Hz, 1 H, -C**H**F), 3.84 (s, 3 H, -C**H$_3$**); **^{13}C-NMR** (175 MHz, $CDCl_3$, 298 K) δ / ppm = 168.8 (d, J = 25.7 Hz, 1 C, -**C**=O), 135.6 (d, J = 11.2 Hz, 1 C, **C$_{Ar}$**), 135.2 (d, J = 1.4 Hz, 1 C, =**C**H), 128.7 (s, 2 C, **C$_{Ar}$**), 128.4 (s, 1 C, **C$_{Ar}$**), 127.0 (d, J = 1.8 Hz, 2 C, **C$_{Ar}$**), 120.9 (d, J = 19.2 Hz, 1 C, =**C**H), 88.6 (d, J = 183.9 Hz, 1 C, -**C**F), 52.7 (s, 1 C, -O**C**H$_3$); **^{19}F-NMR** (282 MHz, $CDCl_3$, 298 K) δ / ppm = -176.3 (ddd, J_1 = 48.3 Hz, J_2 = 13.7 Hz, J_3 = 3.8 Hz, 1 F); **EI-MS**: *m/z* Calcd for $C_{11}H_{11}FO_2$ $[M]^{+\cdot}$ 194; Found 194, 135 ($[M-COOMe]^+$). **HPLC** (CHIRALCEL OJ-H, *n*-hexane/IPA = 7/3, flow rate = 1.0 mL min^{-1}, *I* = 220 nm): t_r = 14.4 min *(major)*, 17.4 min *(minor)*.

Benzyl 2-fluoro-2-phenylacetate 5n

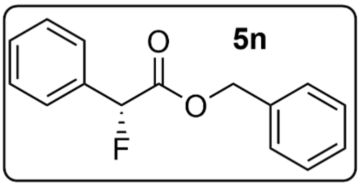

Fluorinated ester *(R)*-**5n** (*er* 98:2) was obtained as a colourless oil in an isolated yield of 8.1 mg (33%) and an initial NMR yield of 70% via the standard procedure. Analytical data was in accordance with literature.[17] **TLC** (Toluene/EtOAc 20/1): R_f= 0.56 (UV), $[\alpha]_D^{20}$ = -54.9 (*c* 0.50, $CHCl_3$, 98:2 *e.r.*); **^{1}H-NMR** (300 MHz, $CDCl_3$, 298 K) δ / ppm = 7.49-7.28 (m, 10 H, Ar-**H**), 5.84 (d, J = 47.7 Hz, 1 H, -C**H**F), 5.31 (s, 2 H, -C**H$_2$**); **^{19}F-NMR** (282 MHz, $CDCl_3$, 298 K) δ / ppm = -180.4 (d, J = 47.8 Hz, 1 F); **HRMS** (ESI-TOF): *m/z* Calcd for $C_{15}H_{17}FNO_2$ $[M+NH_4^+]$ 262.1238; Found 262.1233, **HPLC** (CHIRALCEL OJ-H, *n*-hexane/IPA = 7/3, flow rate = 1.0 mL min^{-1}, *I* = 220 nm): t_r = 19.6 min *(minor)*, 20.1 min *(major)*.

Benzhydryl 2-fluoro-2-phenylacetate 5o

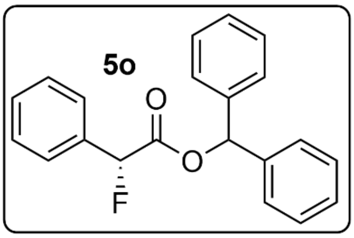

Fluorinated ester *(R)*-**5o** (*er* 98:2) was obtained as a colourless oil in an isolated yield of 13.3 mg (42%) and an initial NMR yield of 69% via the standard procedure. Analytical data was in accordance with literature.[18] **TLC** (Toluene/EtOAc 20/1): R_f= 0.54 (UV), $[\alpha]_D^{20}$ = -38.9 (*c* 0.40, $CHCl_3$, 98:2 *e.r.*); **^{1}H-NMR** (300 MHz, $CDCl_3$, 298 K) δ / ppm = 7.43-7.32 (m, 5 H, Ar-**H**), 7.30-7.25 (m, 5 H, Ar-**H**), 7.19-7.03 (m, 5 H, Ar-**H**), 5.81 (d, J = 47.7 Hz, 1 H, -C**H**F); **^{19}F-NMR** (282 MHz, $CDCl_3$, 298 K) δ / ppm = -180.3 (d, J = 47.5 Hz, 1 F); **HRMS** (ESI-TOF): *m/z* Calcd for $C_{21}H_{17}FKO_2$ $[M+K^+]$ 359.0844; Found 359.0847, **HPLC** (CHIRALCEL OJ-H, *n*-hexane/IPA = 7/3, flow rate = 1.0 mL min^{-1}, *I* = 220 nm): t_r = 33.2 min *(major)*, 36.5 min *(minor)*.

5. Follow-Up Transformations

General Procedure for the Reduction of methyl ester 5a

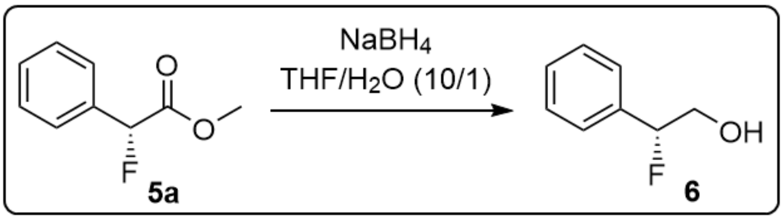

Methyl ester **5a** was prepared according to the general procedure above (0.1 mmol) and was used without further purification. Ester **5a** and 1 mL THF/H_2O (10/1) were added to a round bottom flask and stirred at 0 °C. $NaBH_4$ (37.8 mg, 1.0 mmol) was added in portions. The reaction mixture was stirred at r.t. for 24 h. 1 M HCl was added until bubble formation ceased. The organic phase was separated, and the aqueous phase was extracted thrice with DCM. The combined organic phases were washed with brine, dried with Na_2SO_4 and the solvent was evaporated *in vacuo*. The crude product was purified by column chromatography on silica (heptanes/EtOAc 5/1 → 1/1).

Characterization of 2-fluoro-2-phenylethan-1-ol 6

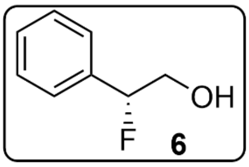

Alcohol *(R)*-**6** (*er* 98:2) was obtained as a colourless oil in a yield of 2.8 mg (20%) over both steps. Analytical data was in accordance with literature.[18] TLC (hexanes/EtOAc 5/1): R_f= 0.11 (UV); ^{1}H-NMR (300 MHz, $CDCl_3$, 298 K) δ / ppm = 7.42-7.32 (m, 5 H, Ar-H), 5.57 (ddd, J_1 = 48.7 Hz, J_2 = 7.4 Hz, J_3 = 3.3 Hz, 1 H, -CHF), 3.98-3.75 (m, 2 H, -CH_2); ^{19}F-NMR (282 MHz, $CDCl_3$, 298 K) δ / ppm = -187.2 (m, 1 F); EI-MS: *m/z* Calcd for C_7H_6F [M – CH_2OH]$^+$ 109; Found 109, 123 ([M – OH]$^+$). HPLC (CHIRALCEL OD-H, *n*-hexane/IPA = 50/1, flow rate = 1.0 mL min^{-1}, *I* = 220 nm): t_r = 53.6 min *(major)*, 56.3 min *(minor)*.

General procedure for the Amidation of methyl ester 5a

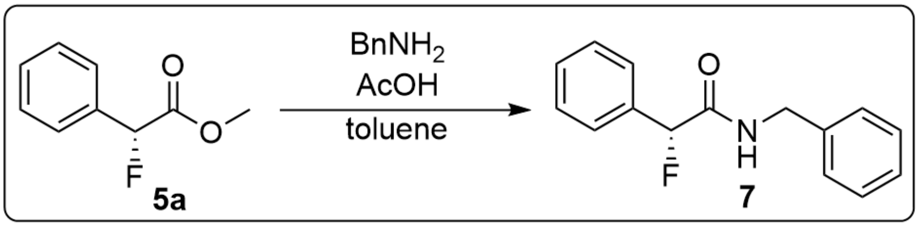

Methyl ester **5a** (23.7 mg, 0.14 mmol), benzyl amine (28 µL, 0.26 mmol), acetic acid (0.8 µL, 10mol%) and 0.4 mL dry toluene were added to a dried pressure Schlenk flask under argon counterflow. The pressure Schlenk flask was sealed, and the reaction mixture was stirred at

110 °C for 20 h. After cooling to r.t., the solvent was removed *in vacuo*. The crude product was purified by column chromatography on deactivated silica (heptanes/EtOAc 3/1→1/1).

Characterization of N-benzyl-2-fluoro-2-phenylacetamide 7

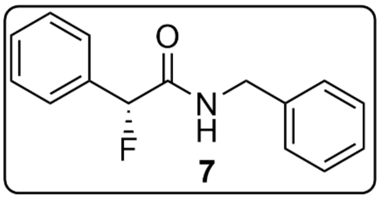

Fluorinated amide *(R)*-**7** (*er* 88:12) was obtained as a colourless oil in a yield of 7.6 mg (39%) via the standard procedure. Analytical data was in accordance with literature.[19] $[\alpha]_D^{20}$ = -32.4 (*c* 0.3, $CHCl_3$, 88:12 *e.r.*); **^{1}H-NMR** (300 MHz, $CDCl_3$, 298 K) *δ* / ppm = 7.48-7.31 (m, 10 H, Ar-**H**), 6.80 (brs, 1 H, -N**H**) 5.83 (d, *J* = 48.4 Hz, 1 H, -C**H**F); **^{13}C-NMR** (75 MHz, $CDCl_3$, 298 K) *δ* / ppm = 168.4 (d, *J* = 21.9 Hz, 1 C, -**C**=O), 137.5 (s, 1 C, $\mathbf{C_{Ar}}$), 134.8 (d, *J* = 19.2 Hz, 1 C, =**C**H), 129.5 (d, *J* = 2.4 Hz, 1 C, $\mathbf{C_{Ar}}$), 128.9 (s, 2 C, $\mathbf{C_{Ar}}$), 128.7 (s, 2 C, $\mathbf{C_{Ar}}$), 127.9 (s, 1 C, $\mathbf{C_{Ar}}$), 126.6 (d, *J* = 6.5 Hz, 2 C, $\mathbf{C_{Ar}}$), 91.9 (d, *J* = 187.8 Hz, 1 C, -**C**F), 43.2 (s, 1 C, -NH**C**H_2); **^{19}F-NMR** (282 MHz, $CDCl_3$, 298 K) *δ* / ppm = -177.8 (dd, J_1 = 48.0 Hz, J_2 = 4.1 Hz, 1 F); **HRMS**: *m/z* Calcd for $C_{15}H_{14}FNO$ $[M+Na]^+$ 266.0952; Found 266.0950 **HPLC** (CHIRALCEL OD-H, *n*-hexane/IPA = 4/1, flow rate = 1.0 mL min^{-1}, *I* = 220 nm): t_r = 11.6 min *(minor)*, 13.7 min *(major)*.

6. References

[1] M. Brindisi, S. Maramai, S. Gemma, S. Brogi, A. Grillo, L. Di Cesare Mannelli, E. Gabellieri, S. Lamponi, S. Saponara, B. Gorelli et al., J. Med. Chem., 59 (2016), 2612–2632.

[2] T. H. West, D. M. Walden, J. E. Taylor, A. C. Brueckner, R. C. Johnston, P. H.-Y. Cheong, G. C. Lloyd-Jones, A. D. Smith, J. Am. Chem. Soc., 139 (2017), 4366–4375.

[3] C. McLaughlin, A. M. Z. Slawin, A. D. Smith, Angew. Chem. Int. Ed., 58 (2019), 15111–15119.

[4] K. J. Schwarz, J. L. Amos, J. C. Klein, D. T. Do, T. N. Snaddon, J. Am. Chem. Soc., 138 (2016), 5214–5217.

[5] L. Stockhammer, D. Weinzierl, T. Bögl, M. Waser, Org. Lett., 23 (2021), 6143–6147.

[6] F. Zhao, C. Shu, C. M. Young, C. Carpenter-Warren, A. M. Z. Slawin, A. D. Smith, Angew. Chem. Int. Ed., 60 (2021), 11892–11900.

[7] K. J. Schwarz, C. M. Pearson, G. A. Cintron-Rosado, P. Liu, T. N. Snaddon, Angew. Chem. Int. Ed., 57 (2018), 7800–7803.

[8] X. Jiang, J. J. Beiger, J. F. Hartwig, J. Am. Chem. Soc., 139 (2017), 87–90.

[9] L. Gui Ning, S. Wang, X. Feng Hu, C. Ming Li, L. Qun Xu, J. Mater. Chem. B, 5 (2017), 8814–8820.

[10] M.-C. Fu, R. Shang, W.-M. Cheng, Y. Fu, Chem. Eur. J., 23 (2017), 8818–8822.

[11] J. Song, Z.-J. Zhang, S.-S. Chen, T. Fan, L.-Z. Gong, Am. Chem. Soc., 140 (2018), 3177-3180.

[12] S. Bresciani, D. O'Hagan, Tetrahedron Lett., 51 (2010), 5795-5797.

[13] E. E. Gray, M. K. Nielsen, K. A. Choquette, J. A. Kalow, T. J. Graham, A. G. Doyle, J. Am. Chem. Soc., 138 (2016), 10802-10805.

[14] L. S. Munaretto, R. D. Gallo, L. P. Leão, I. D. Jurberg, Org. Biomol. Chem., 20 (2022), 6178-6182.

[15] S. Mizuta, T. Yamaguchi, T. Ishikawa, RSC Adv., 14 (2024), 19062-19066.

[16] Y.-M. Zhao, M. S. Cheung, Z. Lin, J. Sun, Angew. Chem. Int. Ed., 51 (2012), 10197-10416.

[17] W. Li, Z. Lu, G. B. Hammond, B. Xu, Org. Lett., 23 (2021), 9640-9644.

[18] S. Yuan, C. Liao, W.-H. Zheng, Org. Lett., 23 (2021), 4142-4146.

[19] K. Ishihara, K. Nishimura, K. Yamakawa, Angew. Chem. Int. Ed., 59 (2020), 17641-17647.

7. Annex 1: NMR Spectra of Novel Compounds

(*R*)-2-((6-fluorobenzo[d]thiazol-2-yl)amino)-2-phenylethan-1-ol

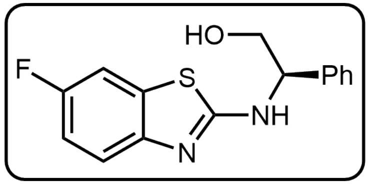

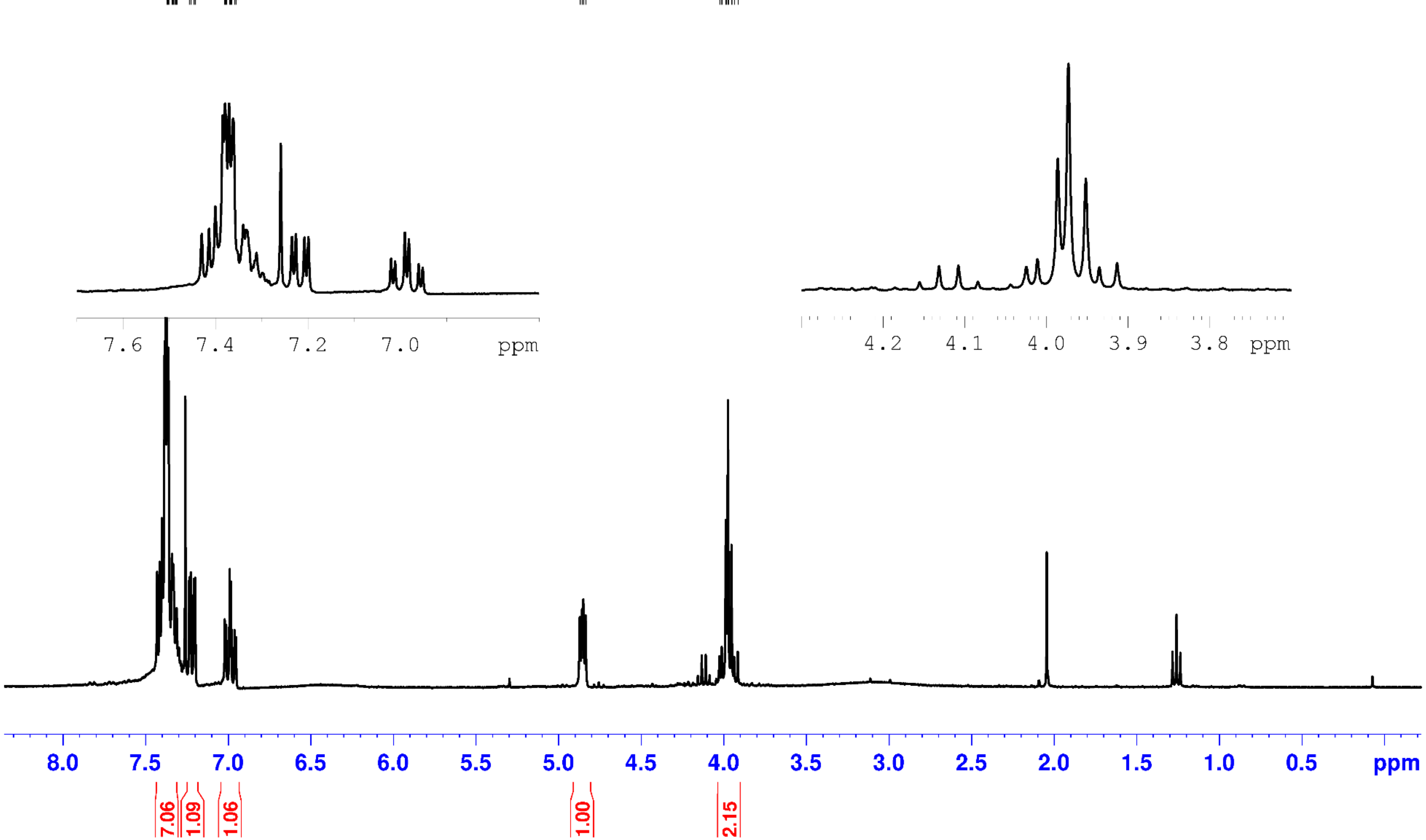

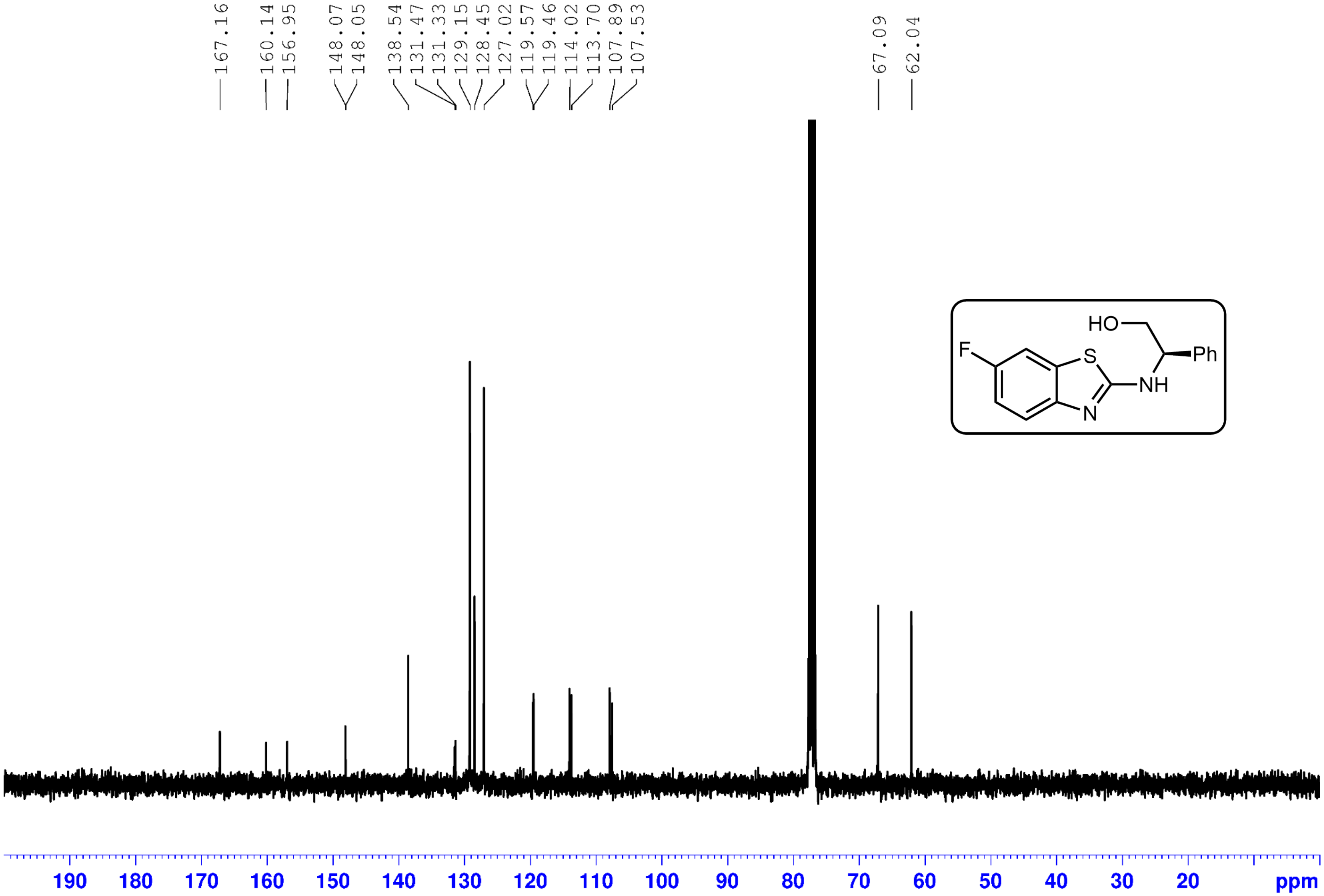

167.16
160.14
156.95
148.07
148.05
138.54
131.47
131.33
129.15
128.45
127.02
119.57
119.46
114.02
113.70
107.89
107.53
67.09
62.04
190
180
170
160
150
140
130
120
110
100
90
80
70
60
50
40
30
20
ppm
HO
F
S
Ph
NH
N

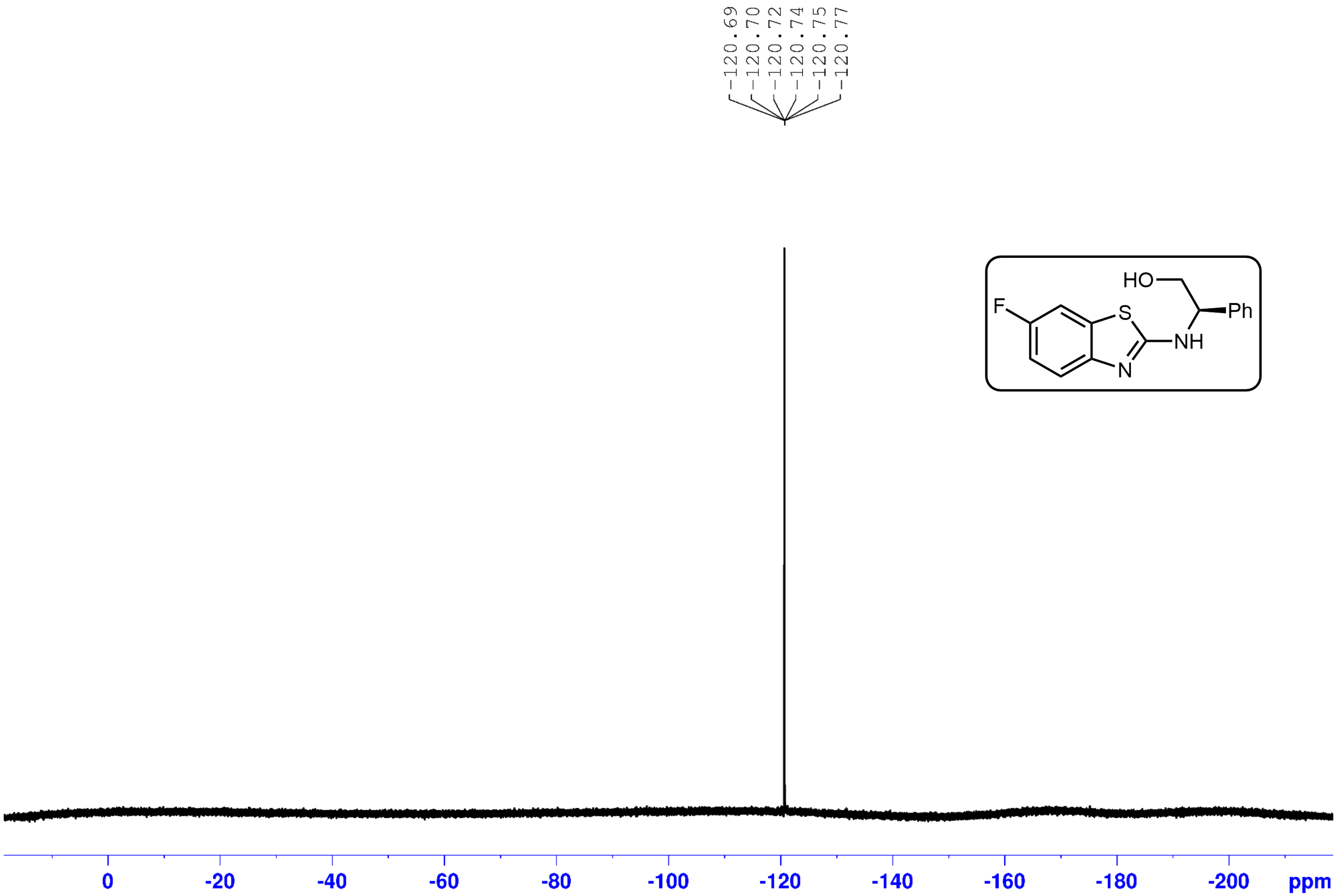

-120.69
-120.70
-120.72
-120.74
-120.75
-120.77
HO
F
S
Ph
NH
N
0
-20
-40
-60
-80
-100
-120
-140
-160
-180
-200
ppm

FBTM

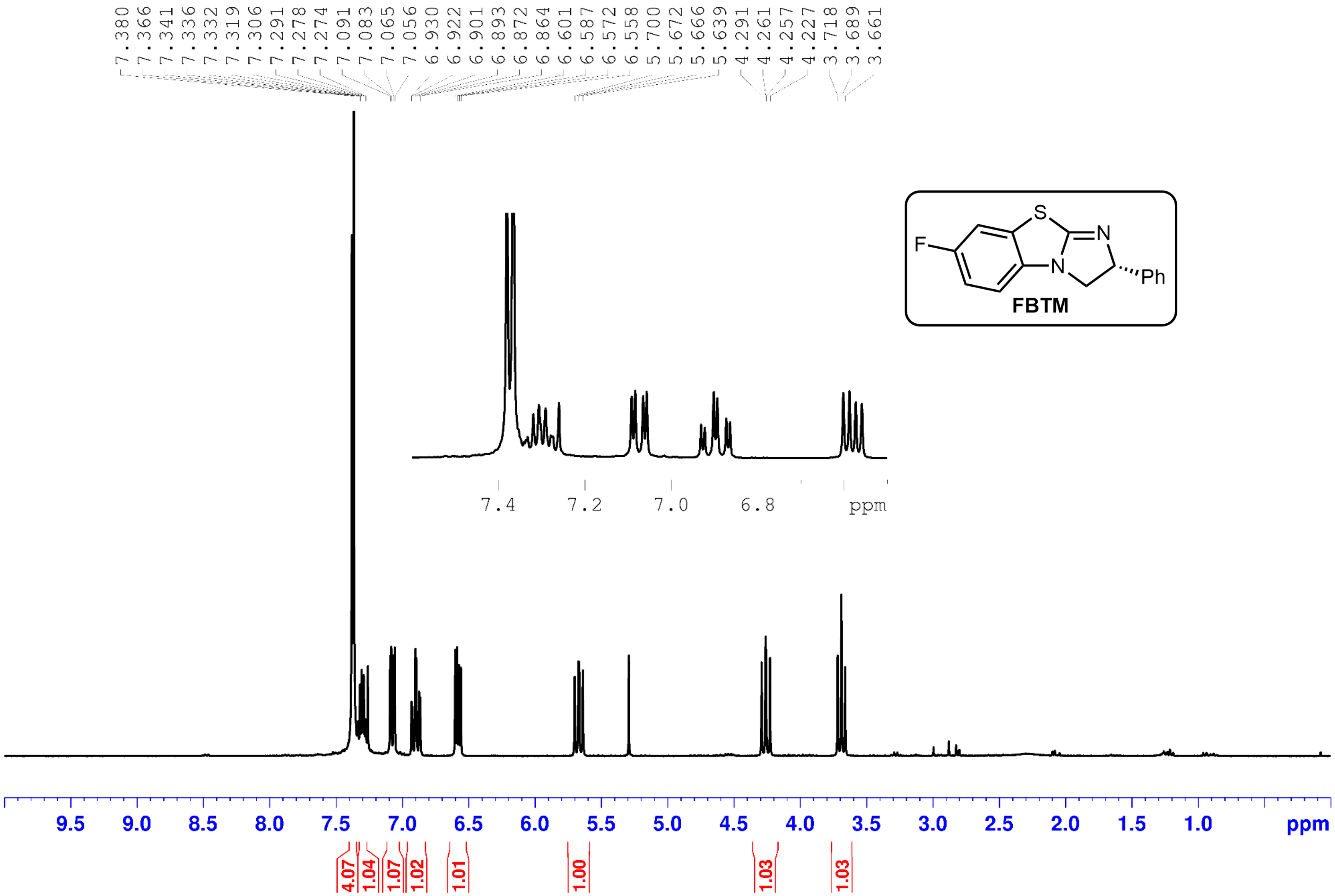
7.380
7.366
7.341
7.336
7.332
7.319
7.306
7.291
7.278
7.274
7.091
7.083
7.065
7.056
6.930
6.922
6.901
6.893
6.872
6.864
6.601
6.587
6.572
6.558
5.700
5.672
5.666
5.639
4.291
4.261
4.257
4.227
3.718
3.689
3.661
7.4
7.2
7.0
6.8
ppm
F
S
N
N
Ph
FBTM
9.5
9.0
8.5
8.0
7.5
7.0
6.5
6.0
5.5
5.0
4.5
4.0
3.5
3.0
2.5
2.0
1.5
1.0
ppm
4.07
1.04
1.07
1.02
1.01
1.00
1.03
1.03

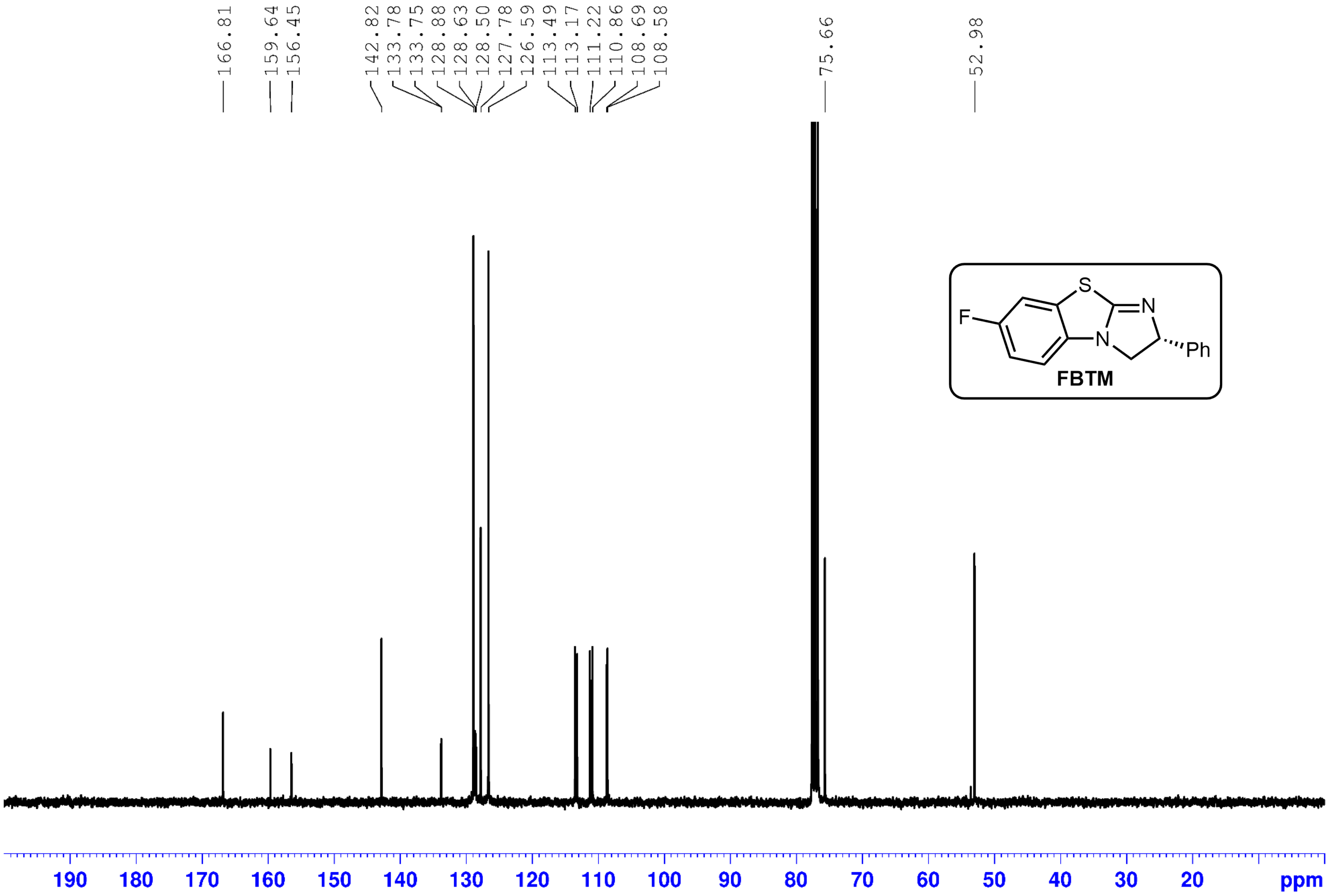

166.81
159.64
156.45
142.82
133.78
133.75
128.88
128.63
128.50
127.78
126.59
113.49
113.17
111.22
110.86
108.69
108.58
75.66
52.98
S
N
N
F
Ph
FBTM
190
180
170
160
150
140
130
120
110
100
90
80
70
60
50
40
30
20
ppm

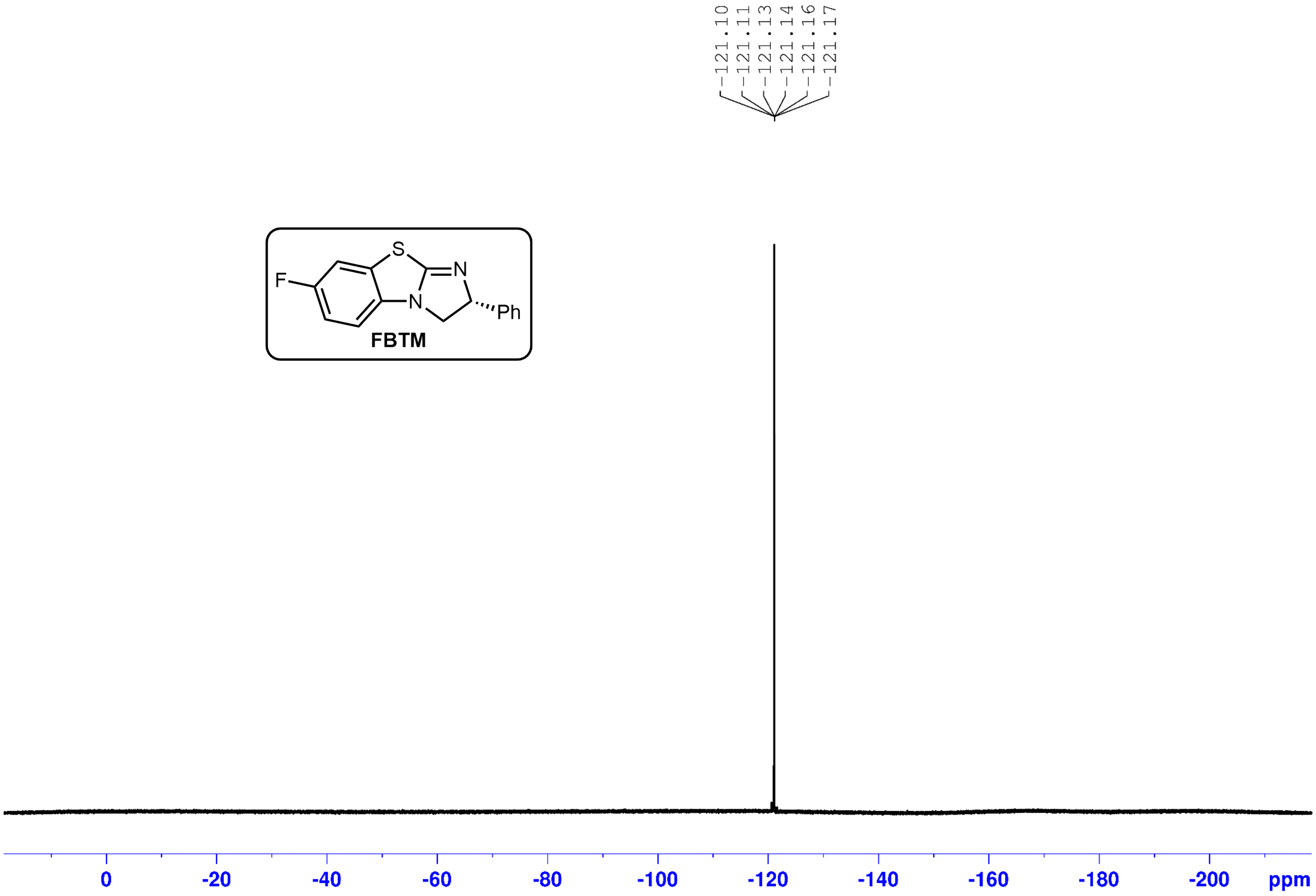
-121.10
-121.11
-121.13
-121.14
-121.16
-121.17
F
S
N
N
Ph
FBTM
0
-20
-40
-60
-80
-100
-120
-140
-160
-180
-200
ppm

Perfluorophenyl 2-(3-iodophenyl)acetate (1j)

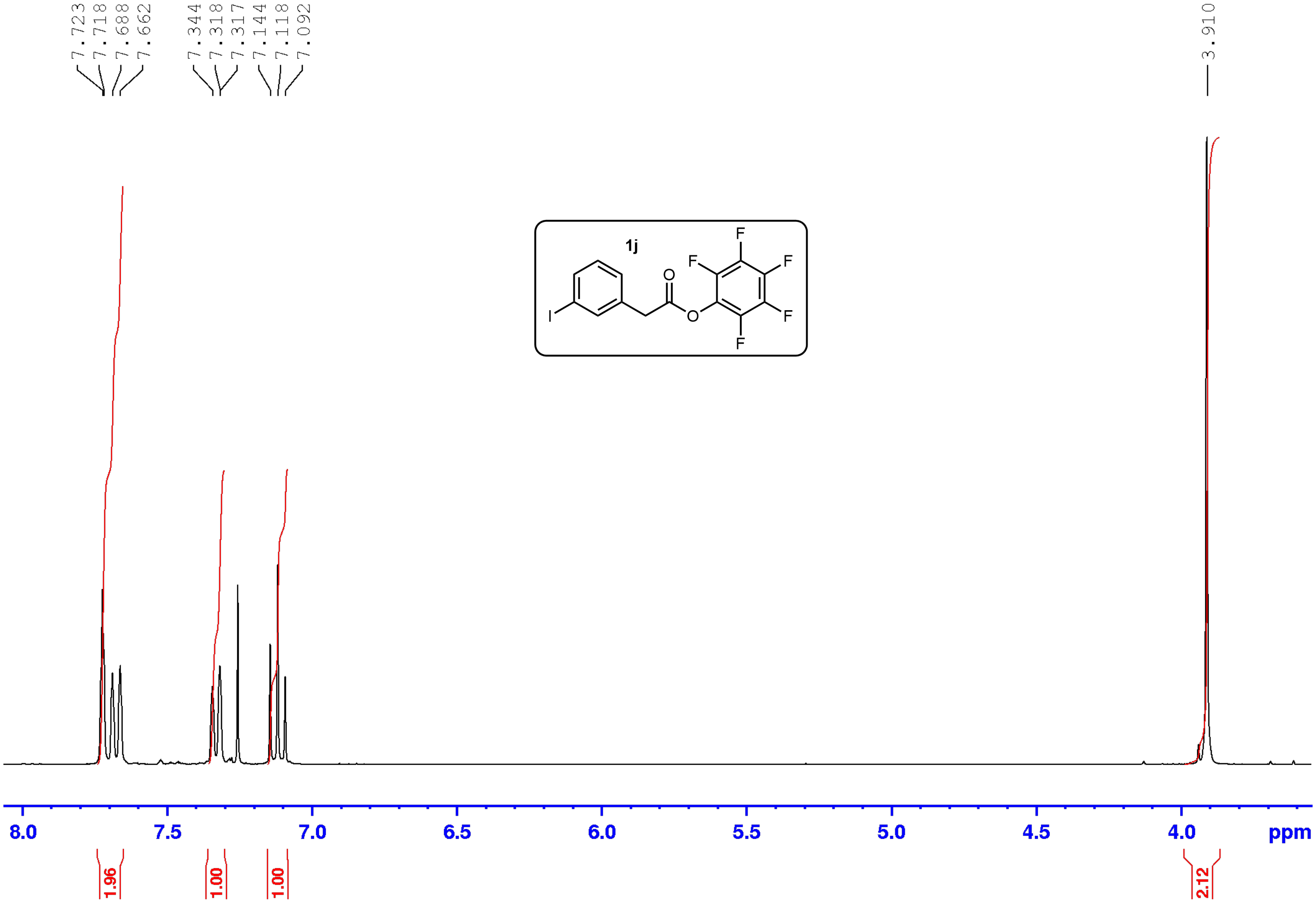

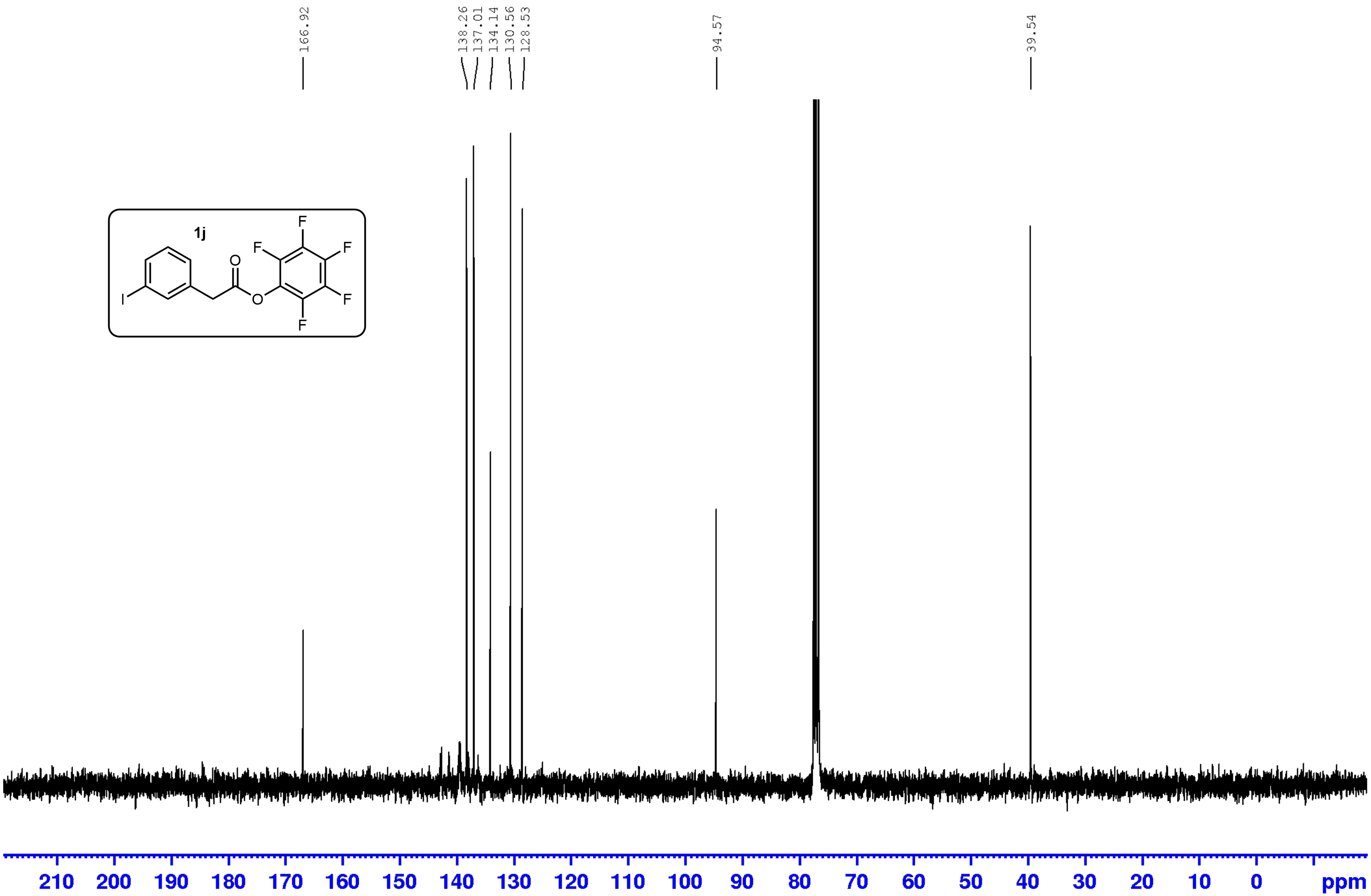
166.92
138.26
137.01
134.14
130.56
128.53
94.57
39.54
1j
F
O
I
O
ppm
210 200 190 180 170 160 150 140 130 120 110 100 90 80 70 60 50 40 30 20 10 0

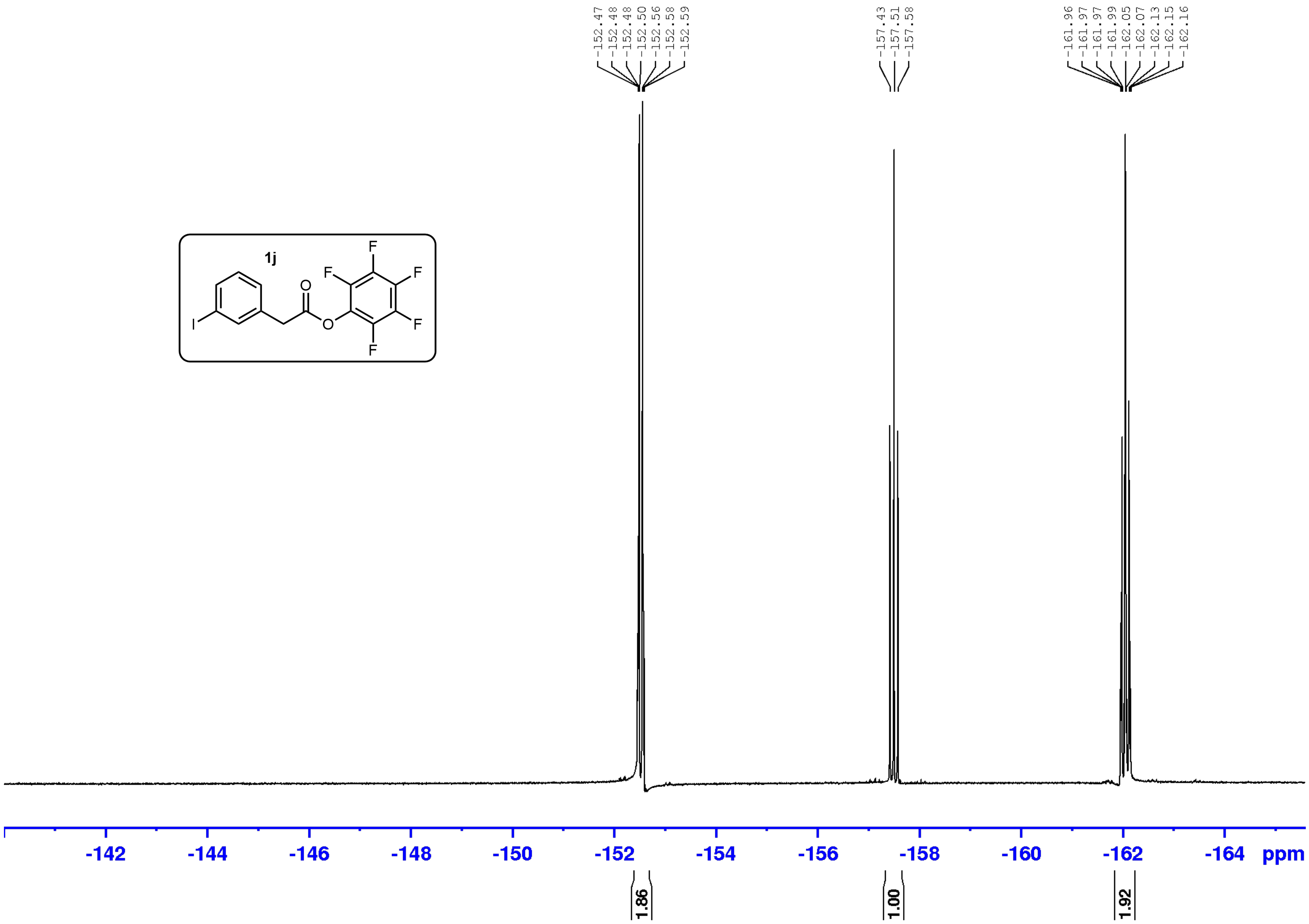

-152.47
-152.48
-152.48
-152.50
-152.56
-152.58
-152.59
-157.43
-157.51
-157.58
-161.96
-161.97
-161.97
-161.99
-162.05
-162.07
-162.13
-162.15
-162.16
1j
F
F
F
O
O
I
F
F
-142
-144
-146
-148
-150
-152
-154
-156
-158
-160
-162
-164
ppm
1.86
1.00
1.92

Methyl 2-fluoro-2-(3-iodophenyl)acetate 5j

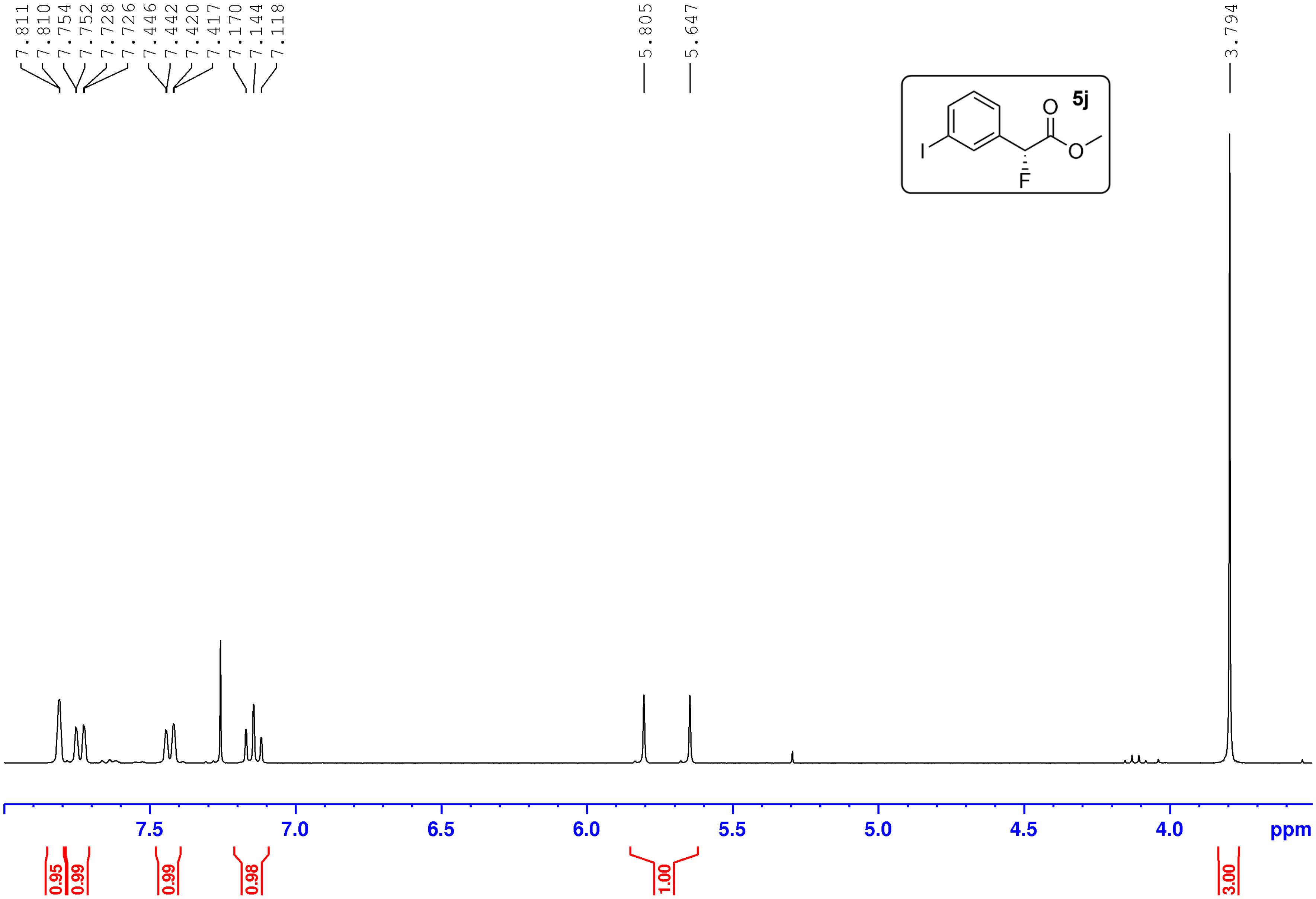

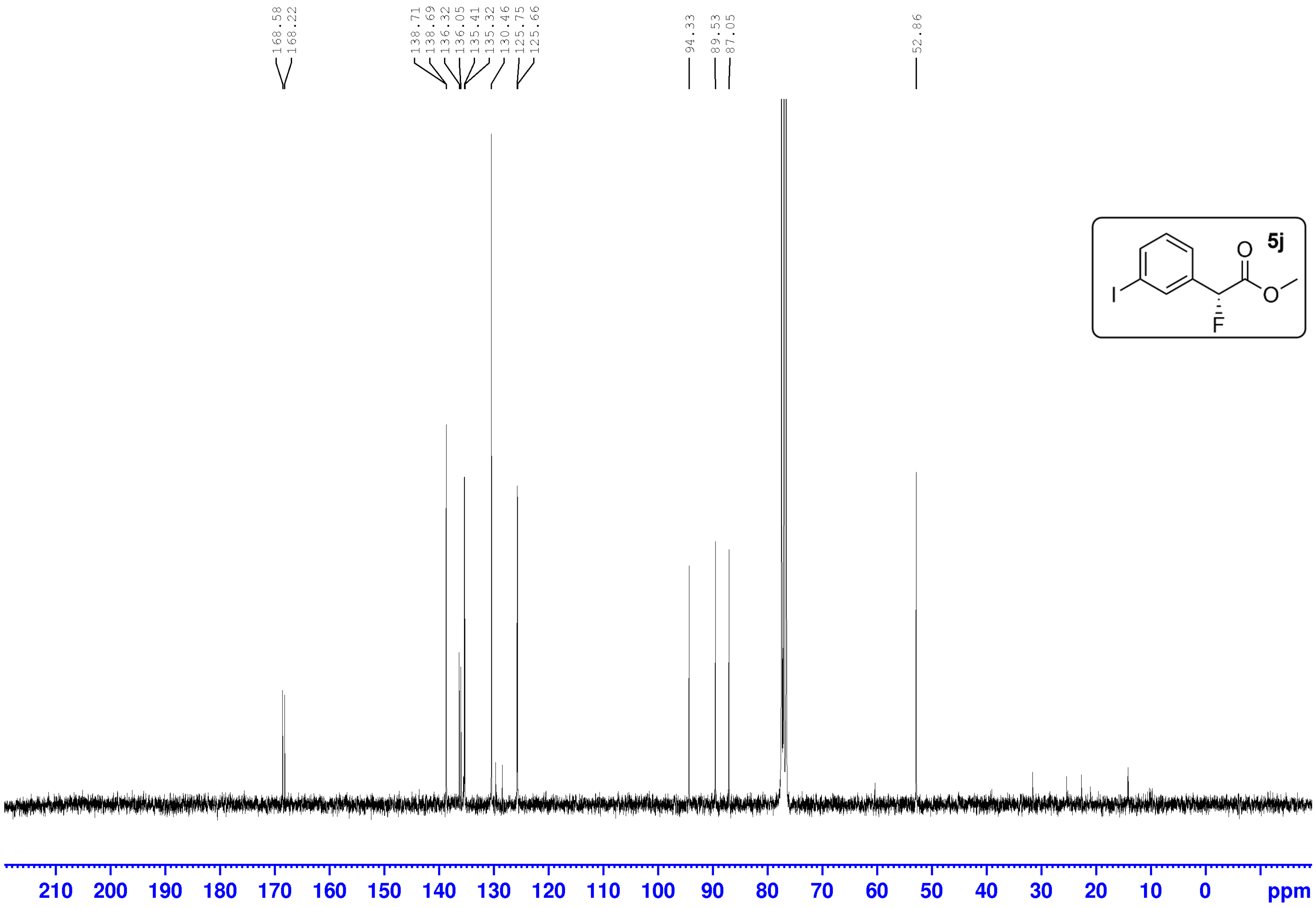

168.58
168.22
138.71
138.69
136.32
136.05
135.41
135.32
130.46
125.75
125.66
94.33
89.53
87.05
52.86
210 200 190 180 170 160 150 140 130 120 110 100 90 80 70 60 50 40 30 20 10 0 ppm
I
O
O
F

5j

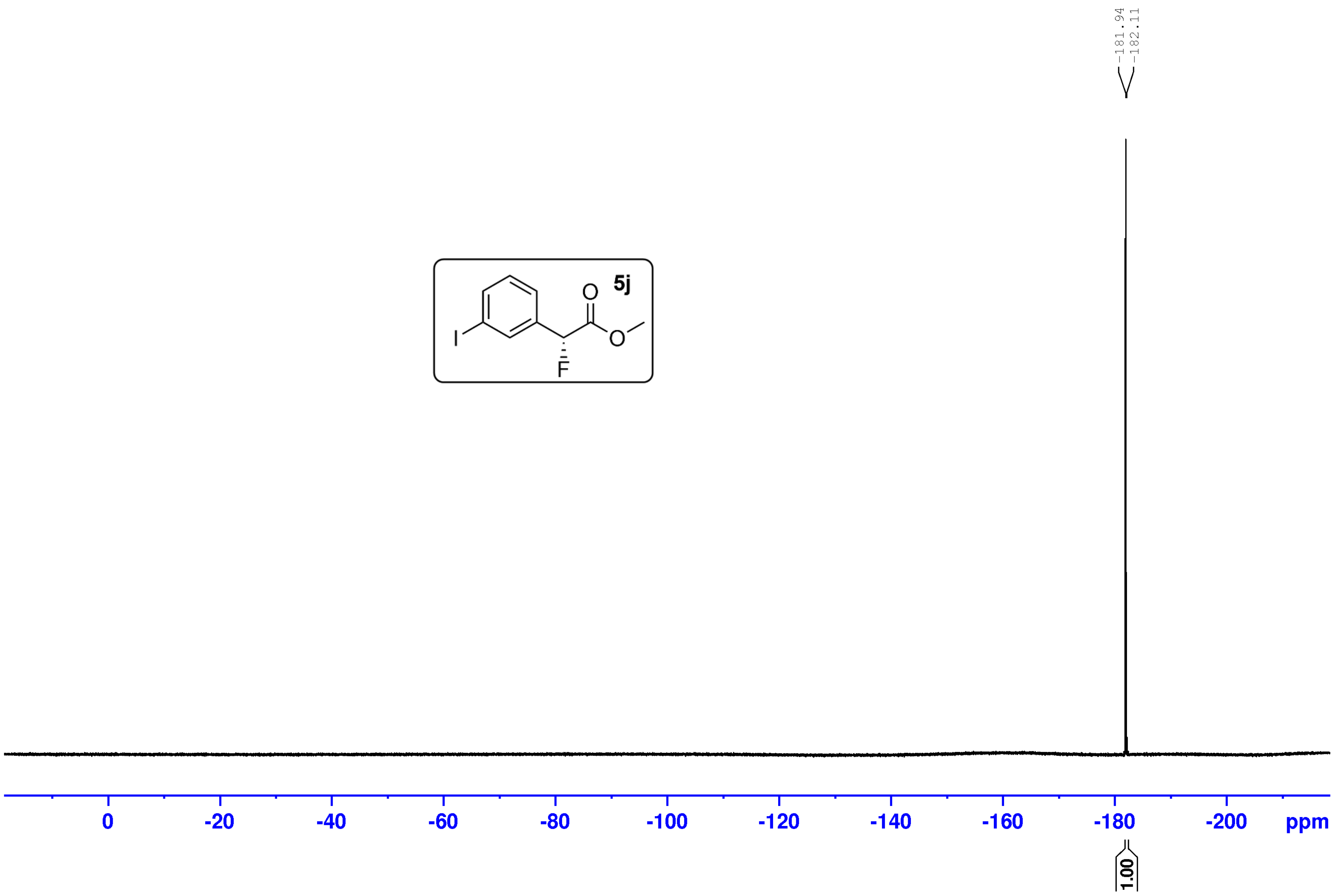

-181.94
-182.11
5j
O
O
I
F
0
-20
-40
-60
-80
-100
-120
-140
-160
-180
-200
ppm
1.00

Methyl 2-fluoro-2-(thiophen-3-yl)acetate 5l

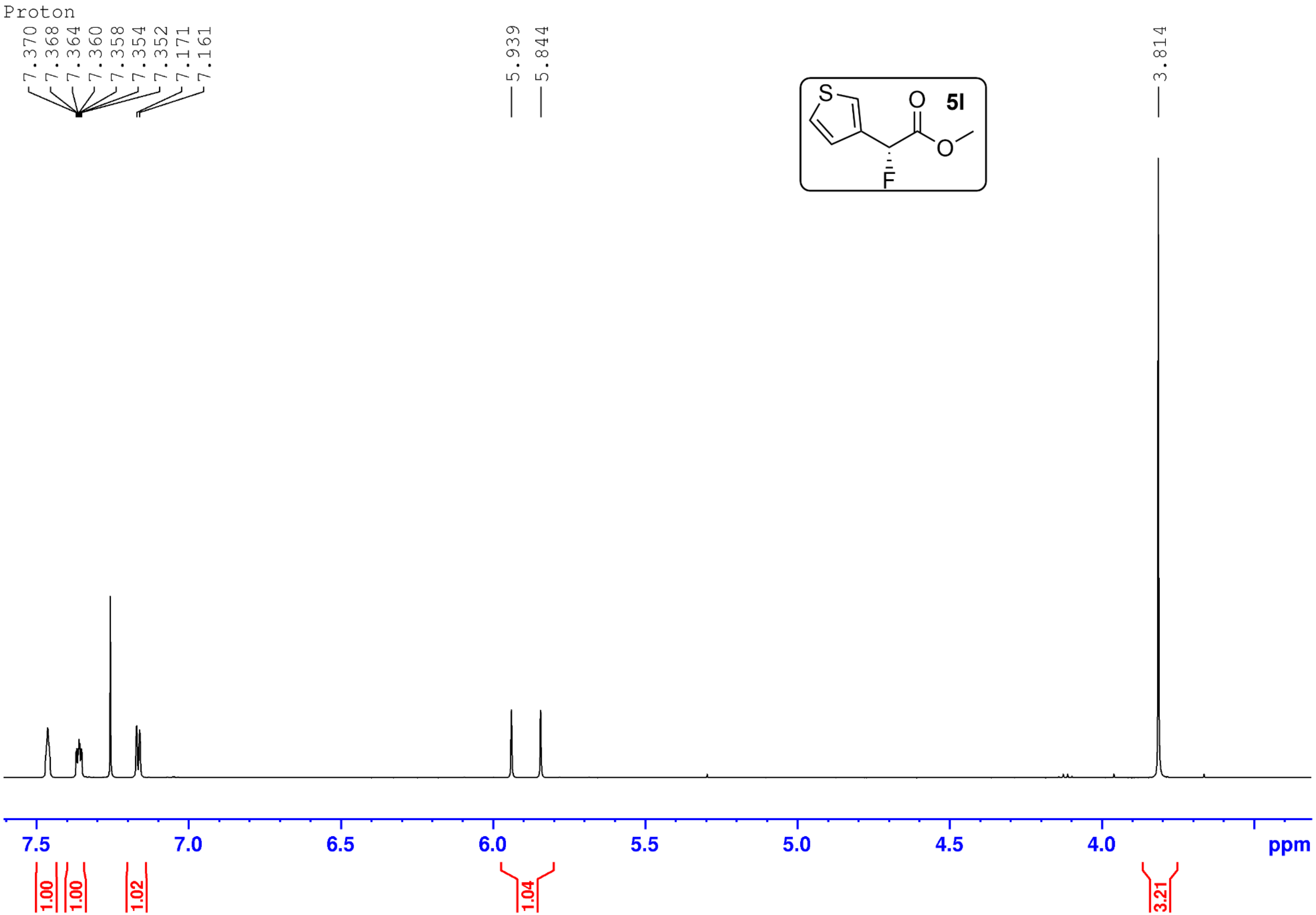

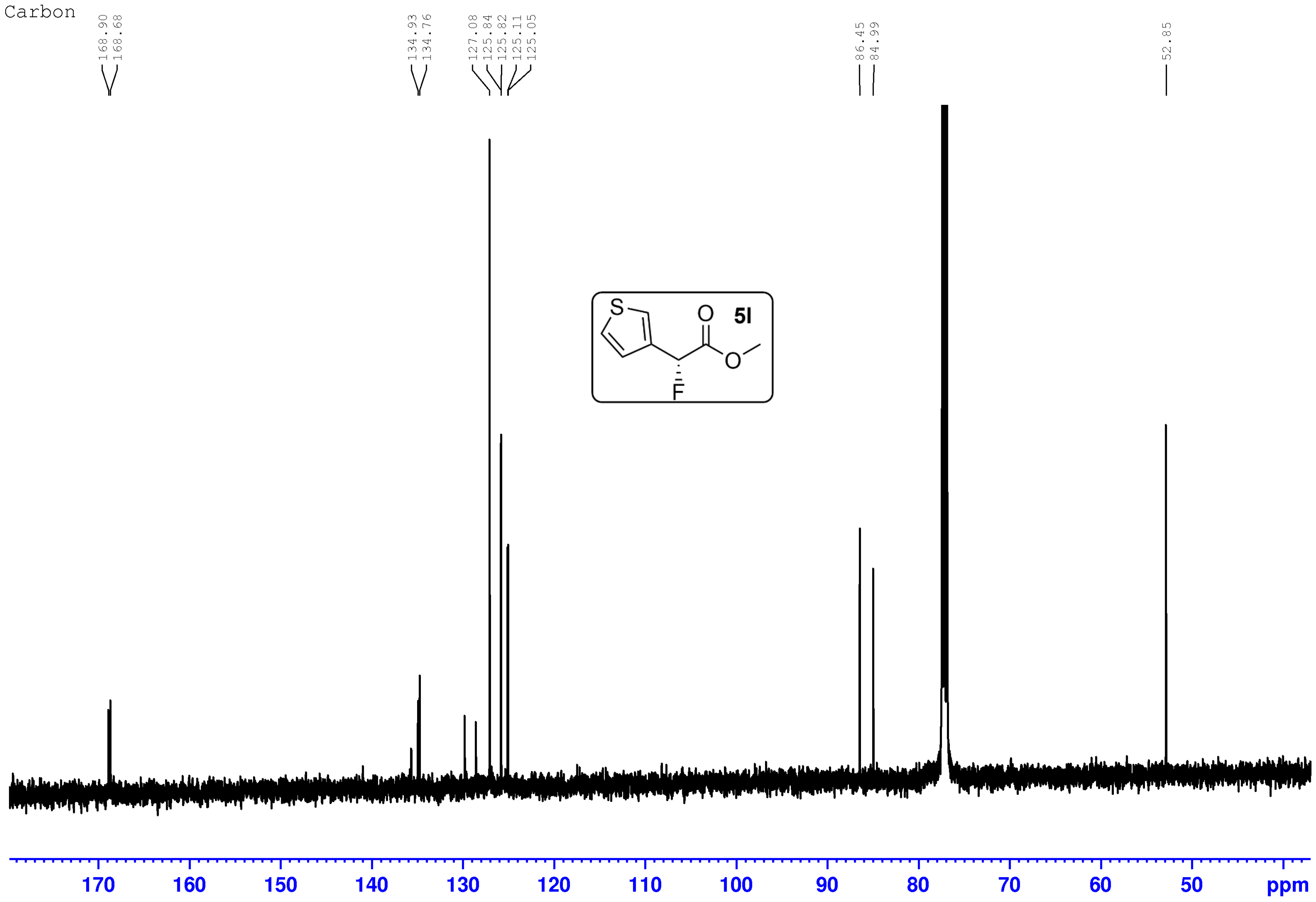
Carbon
168.90
168.68
134.93
134.76
127.08
125.84
125.82
125.11
125.05
86.45
84.99
52.85
5l
S
O
O
F
170
160
150
140
130
120
110
100
90
80
70
60
50
ppm

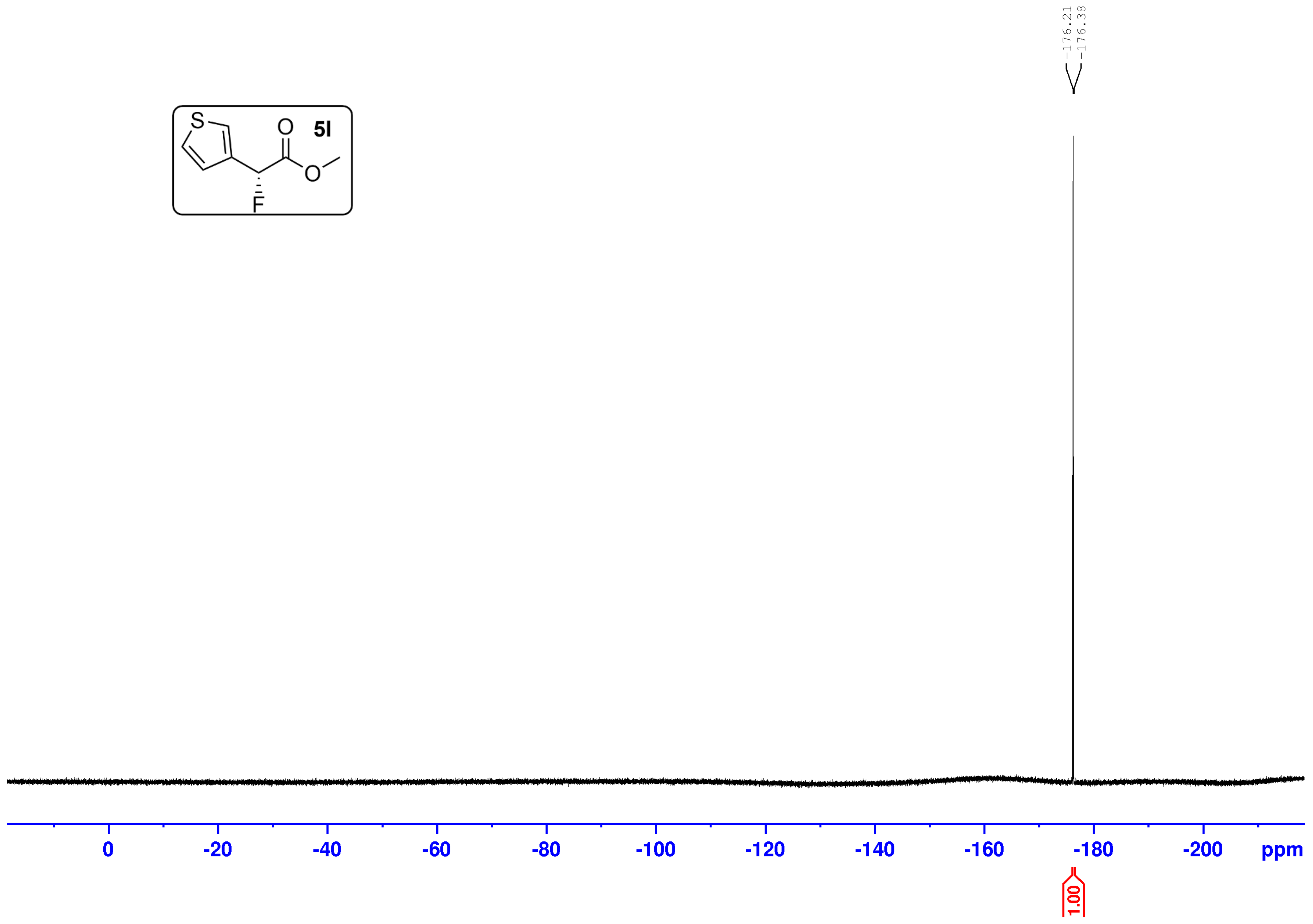
-176.21
-176.38
S
O
O
F
5l
0
-20
-40
-60
-80
-100
-120
-140
-160
-180
-200
ppm
1.00

8. Annex 2: HPLC Chromatograms

HPLC Chromatogram of (rac.)-**5a**

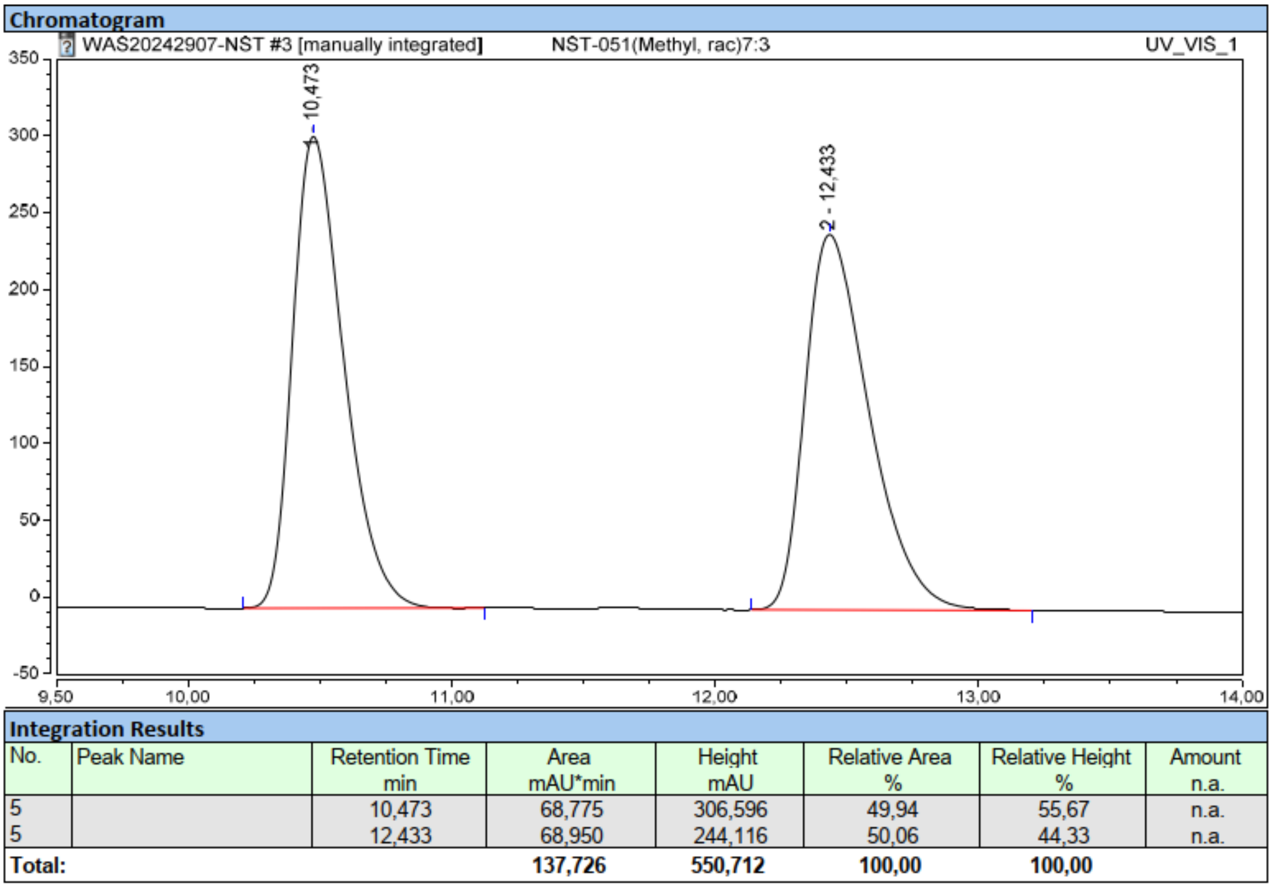

Integration Results							
No.	Peak Name	Retention Time min	Area mAU*min	Height mAU	Relative Area %	Relative Height %	Amount n.a.
5		10,473	68,775	306,596	49,94	55,67	n.a.
5		12,433	68,950	244,116	50,06	44,33	n.a.
**Total:**			**137,726**	**550,712**	**100,00**	**100,00**	

HPLC Chromatogram of enantioenriched-**5a**

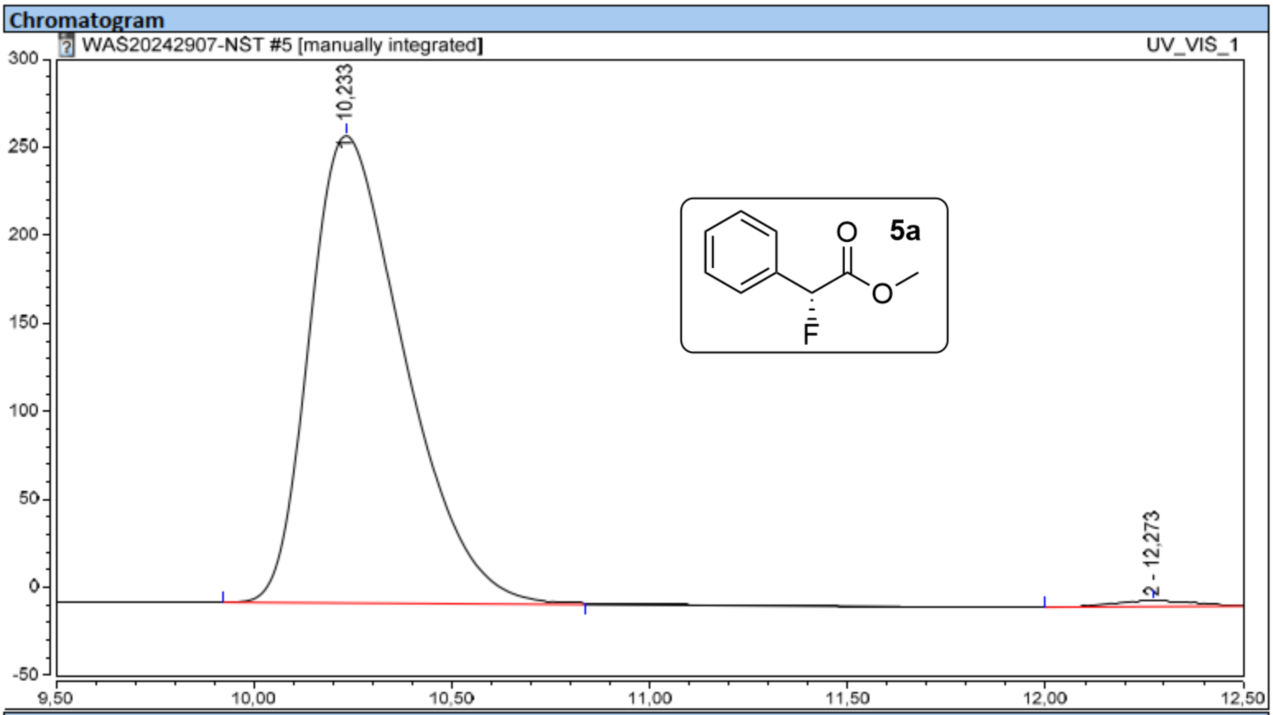

Integration Results							
No.	Peak Name	Retention Time min	Area mAU*min	Height mAU	Relative Area %	Relative Height %	Amount n.a.
5		10,233	73,873	265,359	98,88	98,78	n.a.
5		12,273	0,834	3,289	1,12	1,22	n.a.
**Total:**			**74,707**	**268,649**	**100,00**	**100,00**	

HPLC Chromatogram of (rac.)-**5b**

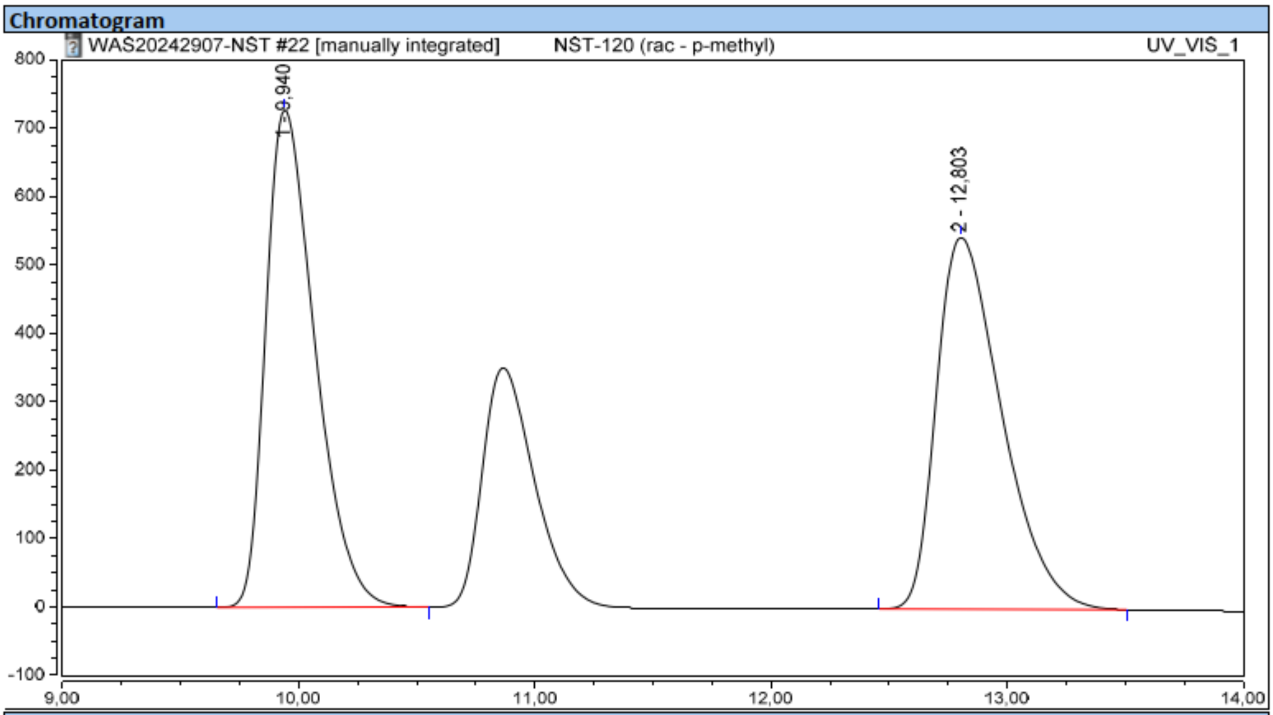

Integration Results							
No.	Peak Name	Retention Time min	Area mAU*min	Height mAU	Relative Area %	Relative Height %	Amount n.a.
5		9,940	175,585	726,851	50,21	57,19	n.a.
5		12,803	174,140	544,044	49,79	42,81	n.a.
**Total:**			**349,725**	**1270,895**	**100,00**	**100,00**	

HPLC Chromatogram of enantioenriched-**5b**

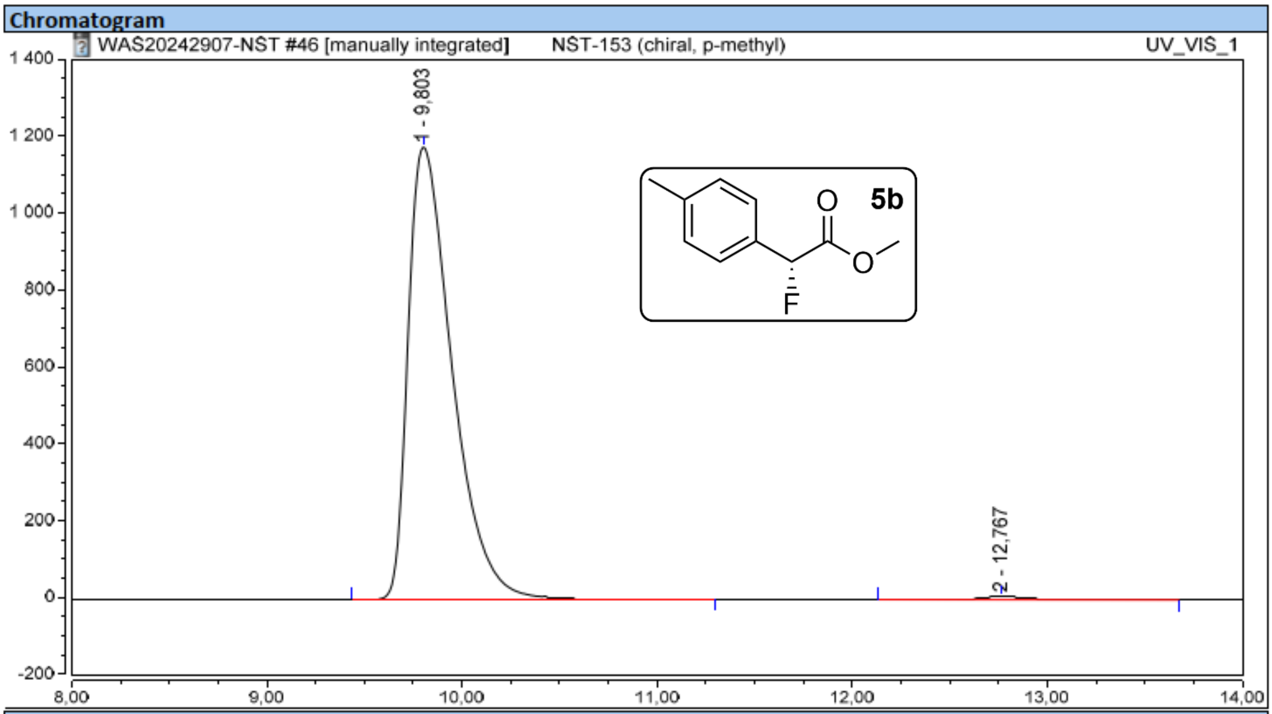

Integration Results							
No.	Peak Name	Retention Time min	Area mAU*min	Height mAU	Relative Area %	Relative Height %	Amount n.a.
5		9,803	307,005	1175,934	99,16	99,29	n.a.
5		12,767	2,605	8,383	0,84	0,71	n.a.
**Total:**			**309,609**	**1184,318**	**100,00**	**100,00**	

HPLC Chromatogram of (rac.)-**5c**

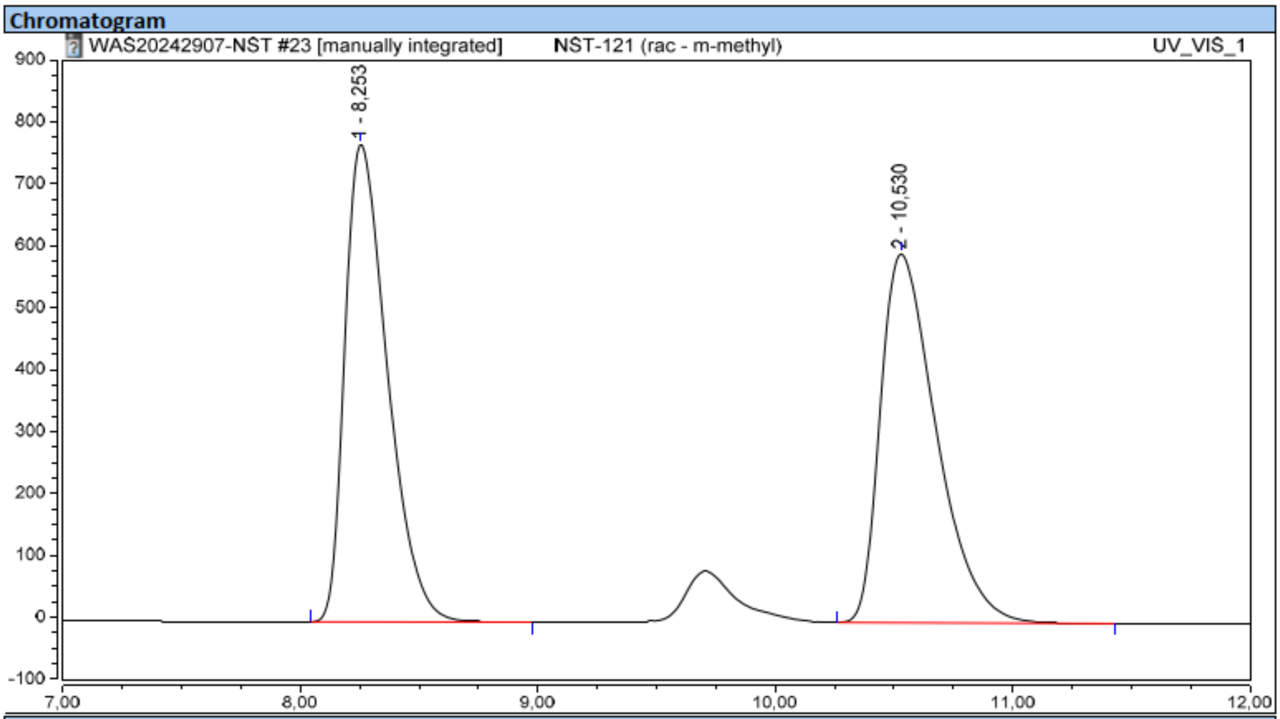

Integration Results							
No.	Peak Name	Retention Time min	Area mAU*min	Height mAU	Relative Area %	Relative Height %	Amount n.a.
5		8,253	160,125	771,528	49,67	56,44	n.a.
5		10,530	162,267	595,374	50,33	43,56	n.a.
**Total:**			**322,392**	**1366,903**	**100,00**	**100,00**	

HPLC Chromatogram of enantioenriched-**5c**

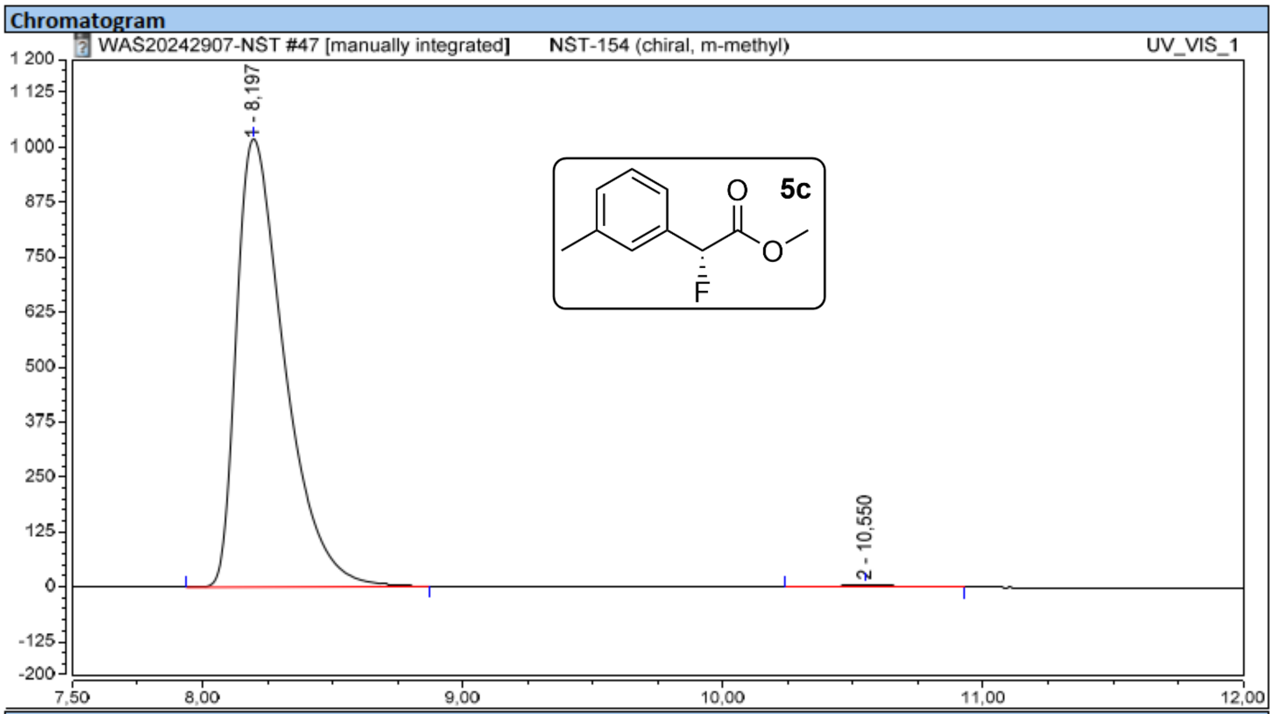

Integration Results							
No.	Peak Name	Retention Time min	Area mAU*min	Height mAU	Relative Area %	Relative Height %	Amount n.a.
5		8,197	217,824	1020,156	99,27	99,36	n.a.
5		10,550	1,596	6,616	0,73	0,64	n.a.
**Total:**			**219,420**	**1026,772**	**100,00**	**100,00**	

HPLC Chromatogram of (rac.)-**5d**

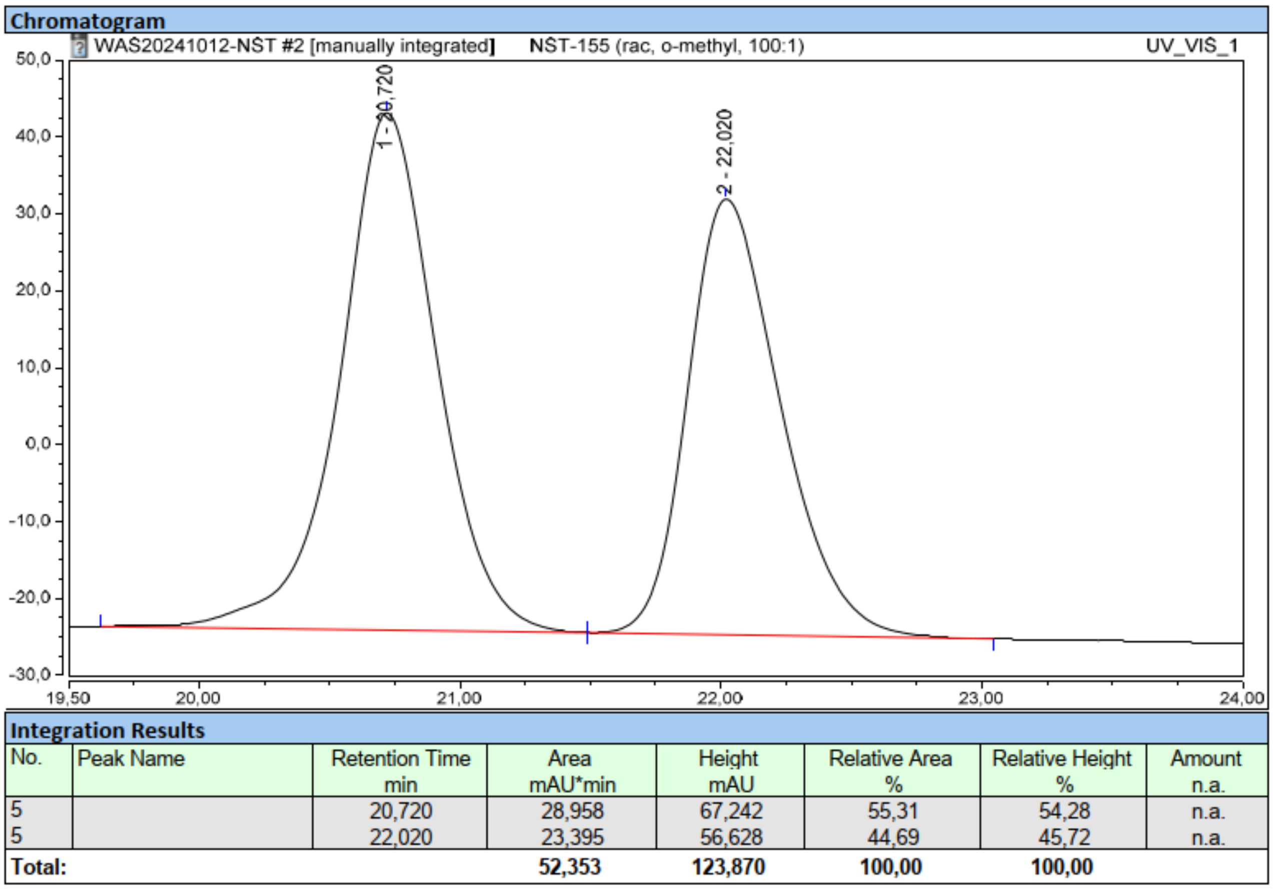

Integration Results

No.	Peak Name	Retention Time min	Area mAU*min	Height mAU	Relative Area %	Relative Height %	Amount n.a.
5		20,720	28,958	67,242	55,31	54,28	n.a.
5		22,020	23,395	56,628	44,69	45,72	n.a.
**Total:**			**52,353**	**123,870**	**100,00**	**100,00**	

HPLC Chromatogram of enantioenriched-**5d**

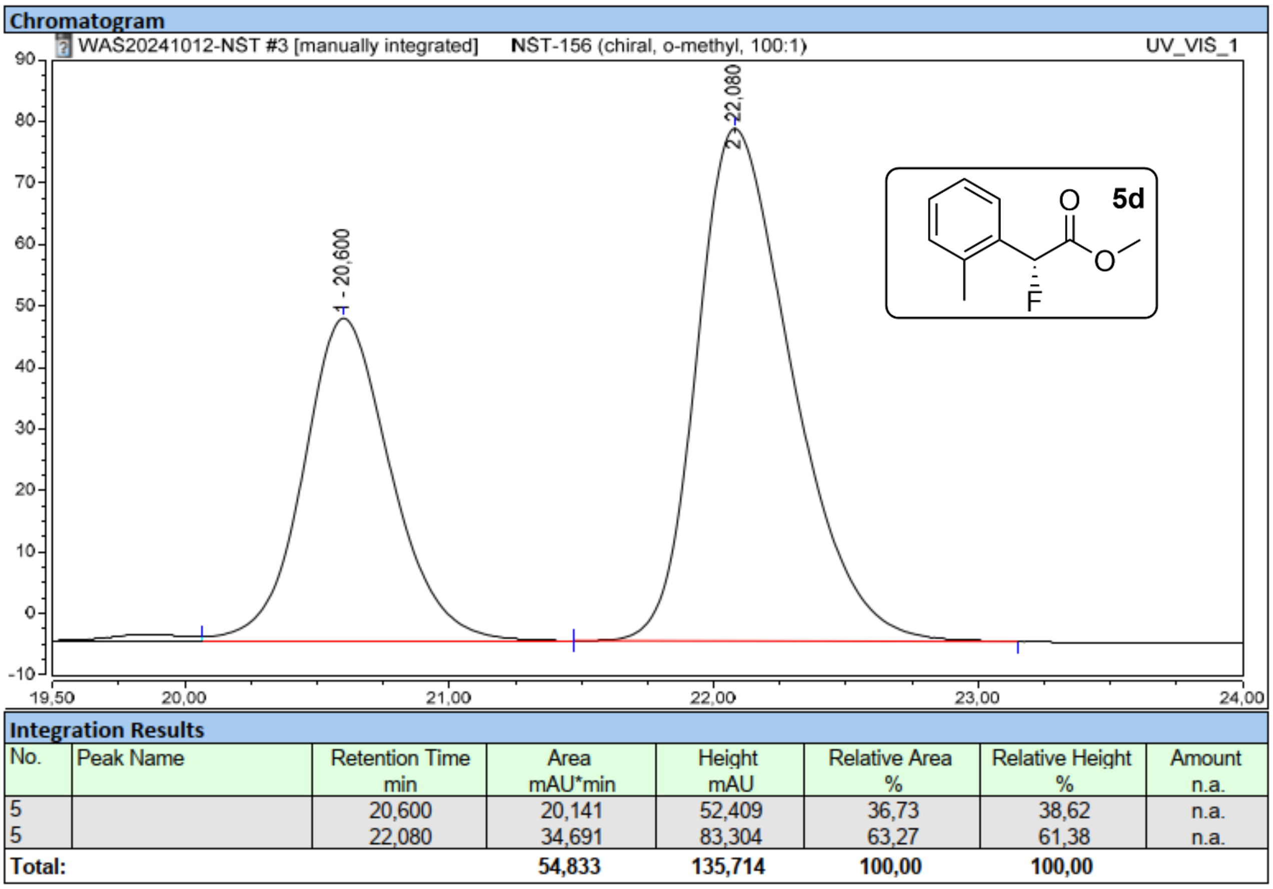

Integration Results

No.	Peak Name	Retention Time min	Area mAU*min	Height mAU	Relative Area %	Relative Height %	Amount n.a.
5		20,600	20,141	52,409	36,73	38,62	n.a.
5		22,080	34,691	83,304	63,27	61,38	n.a.
**Total:**			**54,833**	**135,714**	**100,00**	**100,00**	

HPLC Chromatogram of (rac.)-**5e**

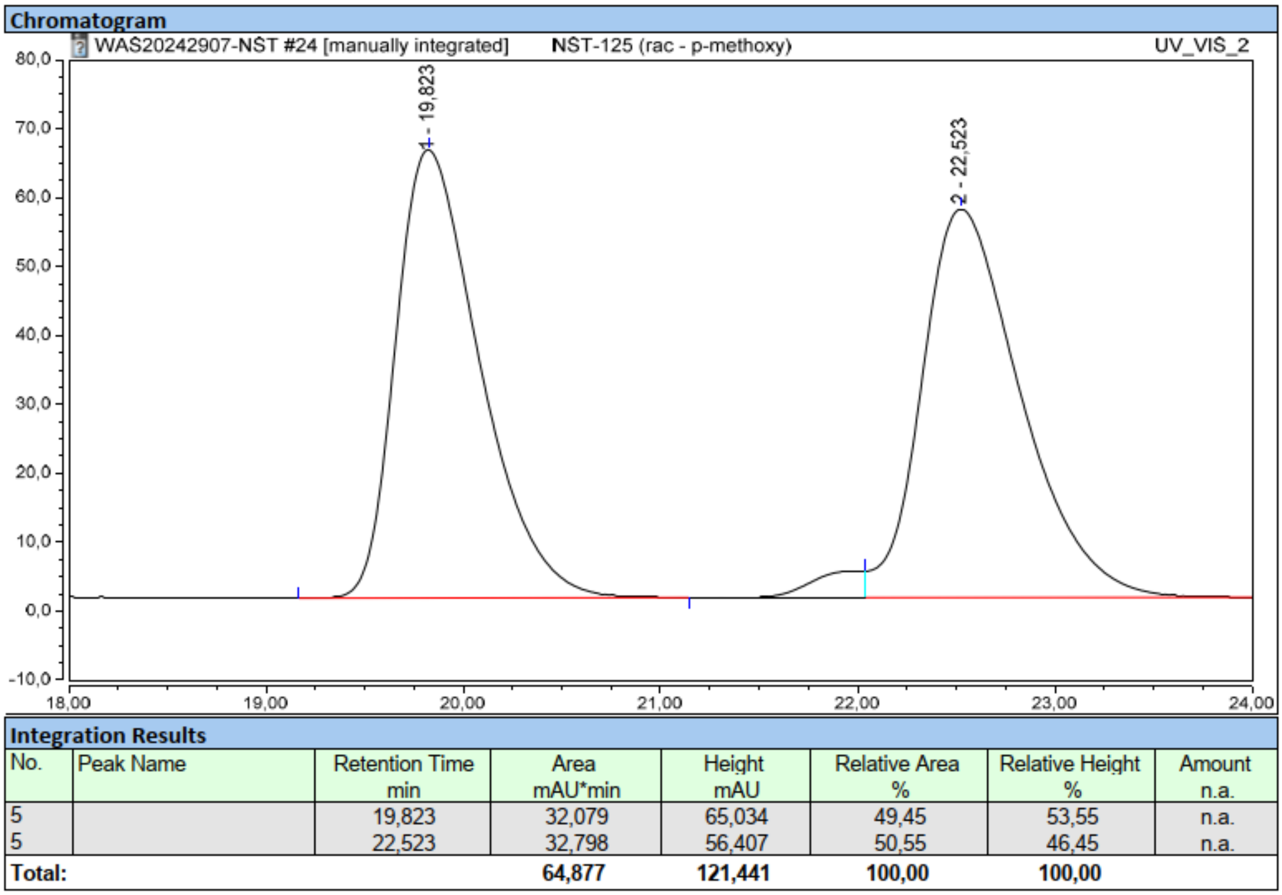

Integration Results

No.	Peak Name	Retention Time min	Area mAU*min	Height mAU	Relative Area %	Relative Height %	Amount n.a.
5		19,823	32,079	65,034	49,45	53,55	n.a.
5		22,523	32,798	56,407	50,55	46,45	n.a.
**Total:**			**64,877**	**121,441**	**100,00**	**100,00**	

HPLC Chromatogram of enantioenriched-**5e**

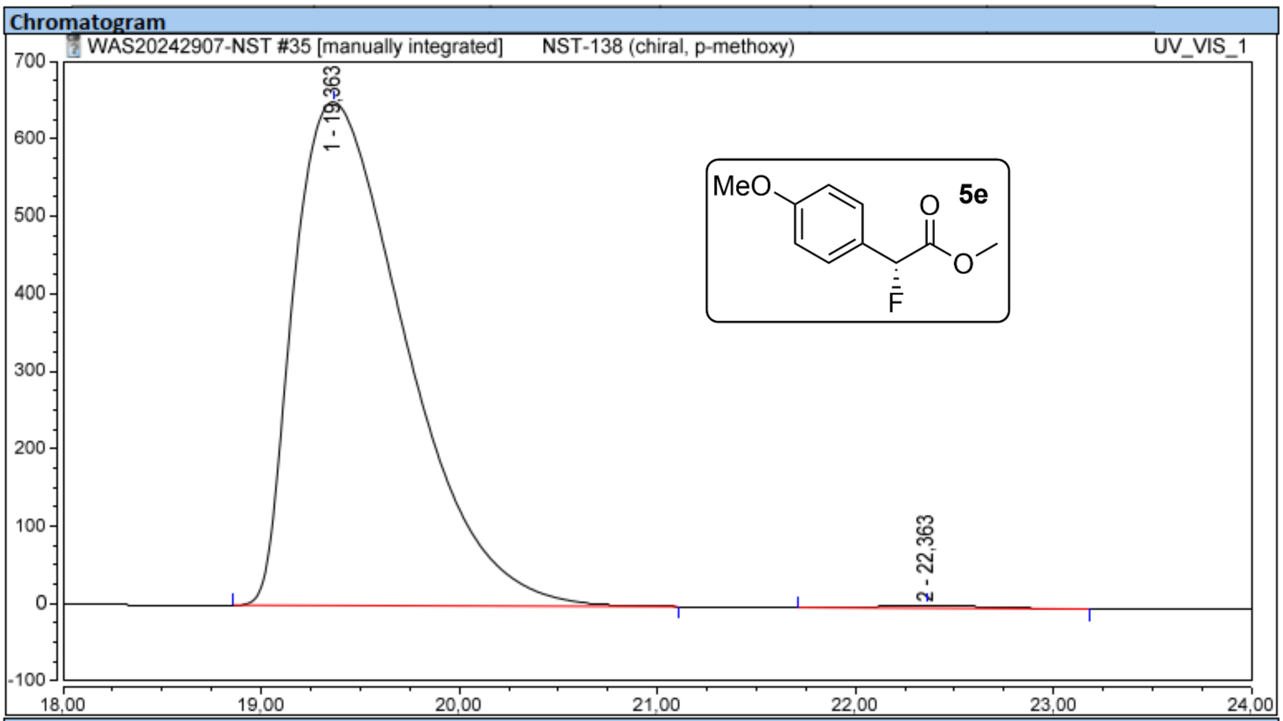

Integration Results

No.	Peak Name	Retention Time min	Area mAU*min	Height mAU	Relative Area %	Relative Height %	Amount n.a.
5		19,363	424,561	649,884	99,49	99,38	n.a.
5		22,363	2,194	4,073	0,51	0,62	n.a.
**Total:**			**426,756**	**653,958**	**100,00**	**100,00**	

HPLC Chromatogram of (rac.)-**5f**

Chromatogram

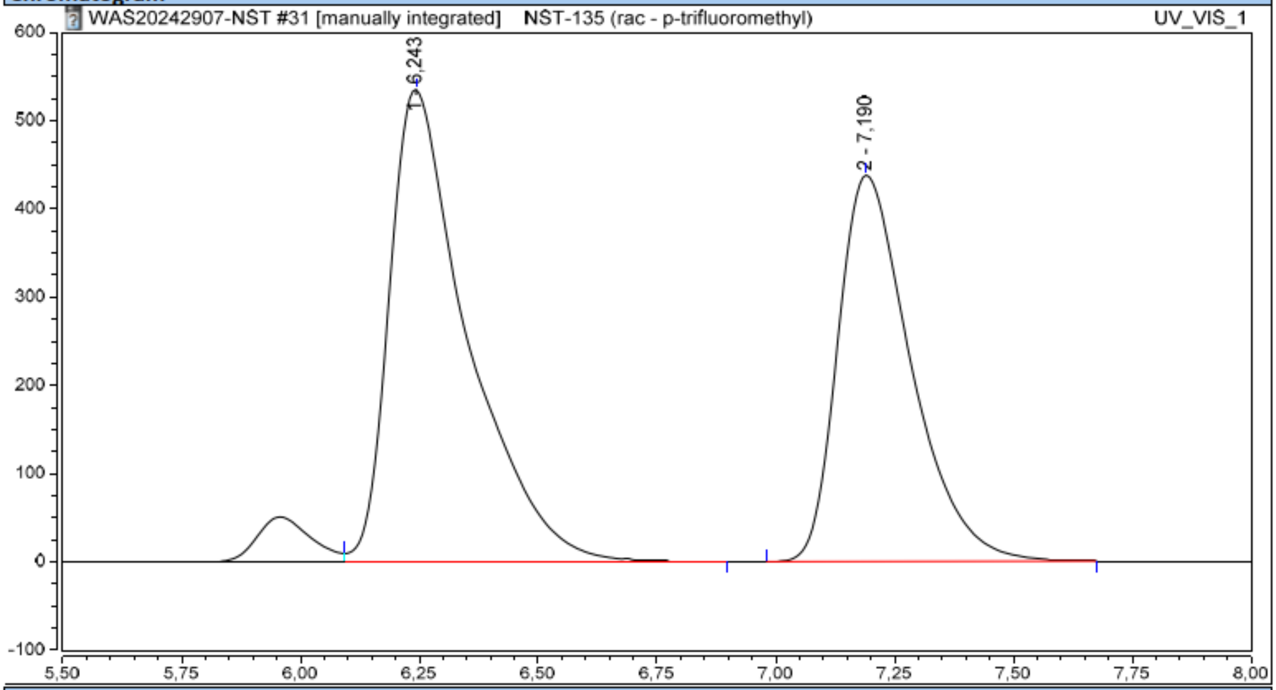

Integration Results

No.	Peak Name	Retention Time min	Area mAU*min	Height mAU	Relative Area %	Relative Height %	Amount n.a.
5		6,243	101,832	534,605	56,63	54,99	n.a.
5		7,190	77,982	437,578	43,37	45,01	n.a.
**Total:**			**179,814**	**972,183**	**100,00**	**100,00**	

HPLC Chromatogram of enantioenriched-**5f**

Chromatogram

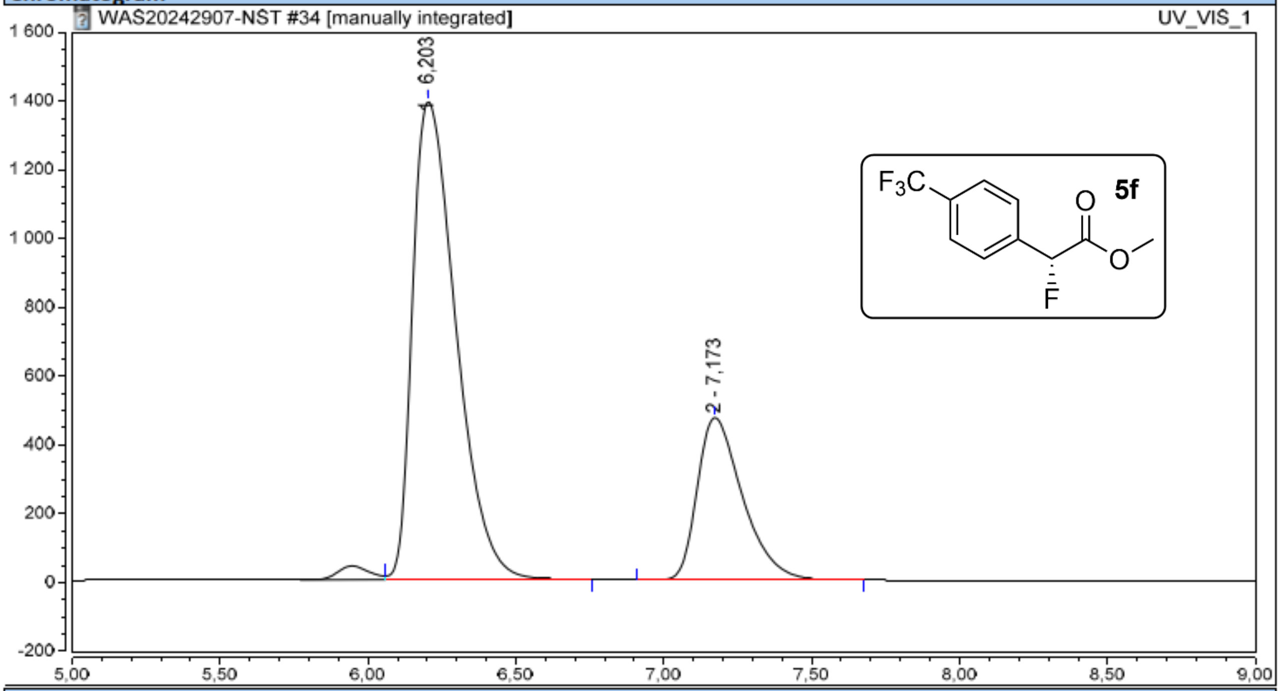

Integration Results

No.	Peak Name	Retention Time min	Area mAU*min	Height mAU	Relative Area %	Relative Height %	Amount n.a.
5		6,203	236,376	1388,846	73,88	74,66	n.a.
5		7,173	83,580	471,302	26,12	25,34	n.a.
**Total:**			**319,955**	**1860,148**	**100,00**	**100,00**	

HPLC Chromatogram of (rac.)-**5g**

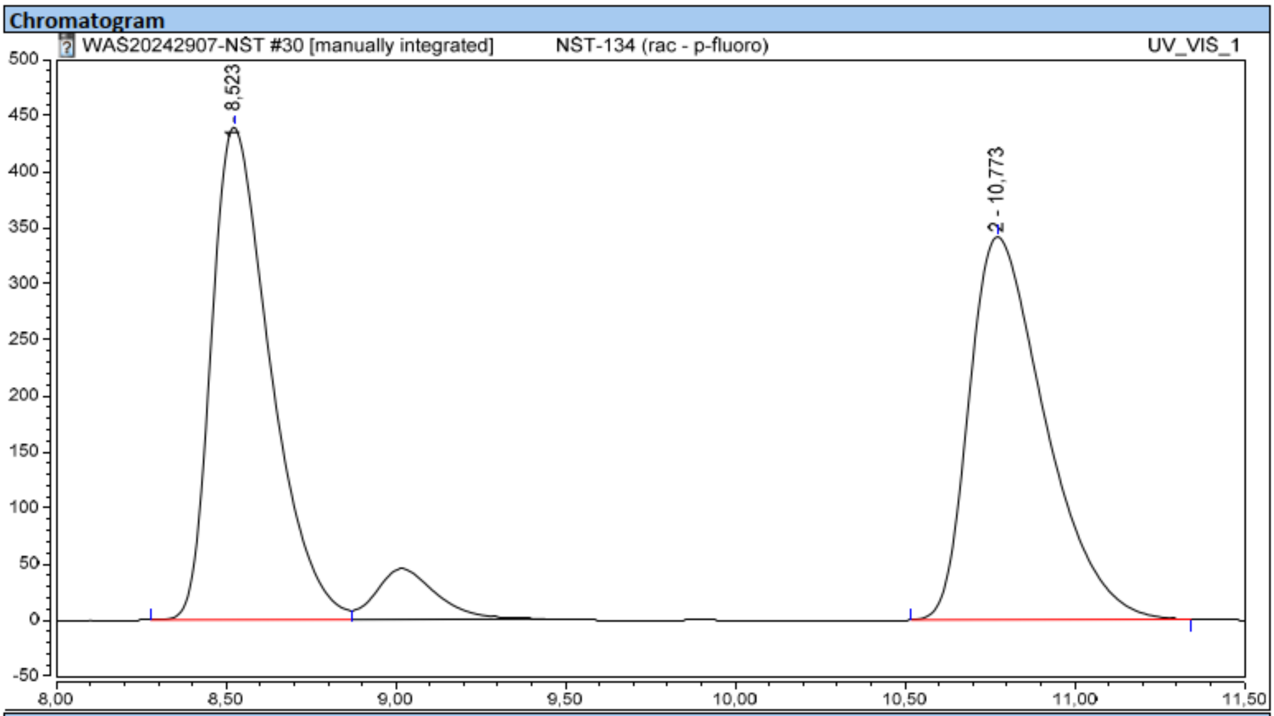

Integration Results

No.	Peak Name	Retention Time min	Area mAU*min	Height mAU	Relative Area %	Relative Height %	Amount n.a.
5		8,523	87,914	439,246	49,79	56,26	n.a.
5		10,773	88,662	341,550	50,21	43,74	n.a.
**Total:**			**176,576**	**780,795**	**100,00**	**100,00**	

HPLC Chromatogram of enantioenriched-**5g**

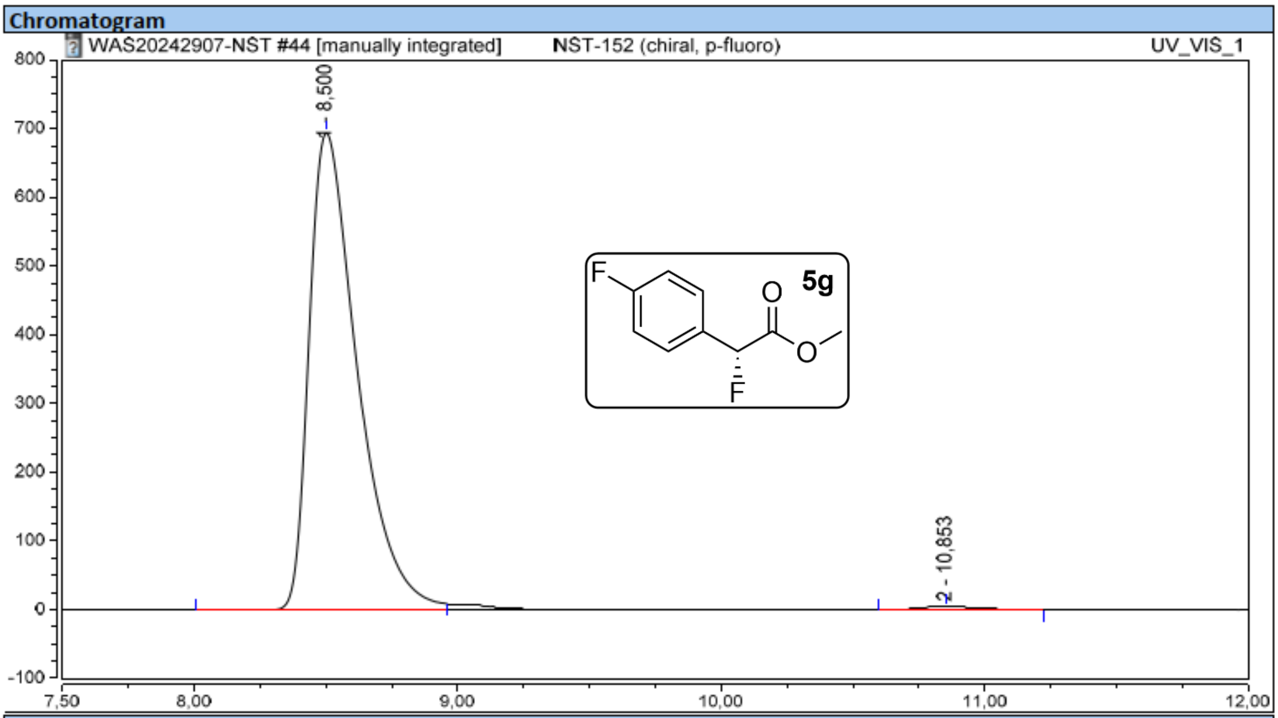

Integration Results

No.	Peak Name	Retention Time min	Area mAU*min	Height mAU	Relative Area %	Relative Height %	Amount n.a.
5		8,500	146,797	693,890	99,11	99,25	n.a.
5		10,853	1,324	5,230	0,89	0,75	n.a.
**Total:**			**148,121**	**699,120**	**100,00**	**100,00**	

HPLC Chromatogram of (rac.)-**5h**

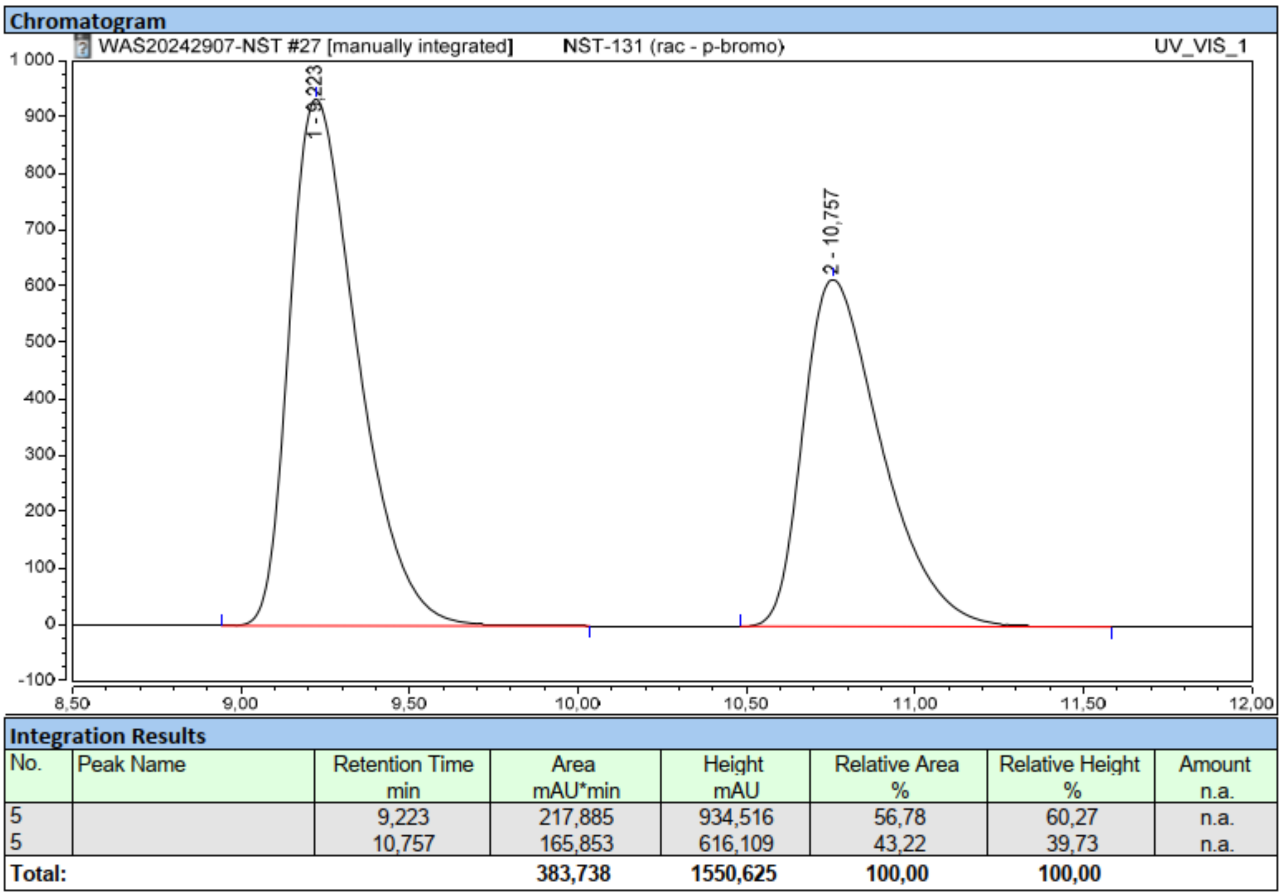

Integration Results

No.	Peak Name	Retention Time min	Area mAU*min	Height mAU	Relative Area %	Relative Height %	Amount n.a.
5		9,223	217,885	934,516	56,78	60,27	n.a.
5		10,757	165,853	616,109	43,22	39,73	n.a.
**Total:**			**383,738**	**1550,625**	**100,00**	**100,00**	

HPLC Chromatogram of enantioenriched-**5h**

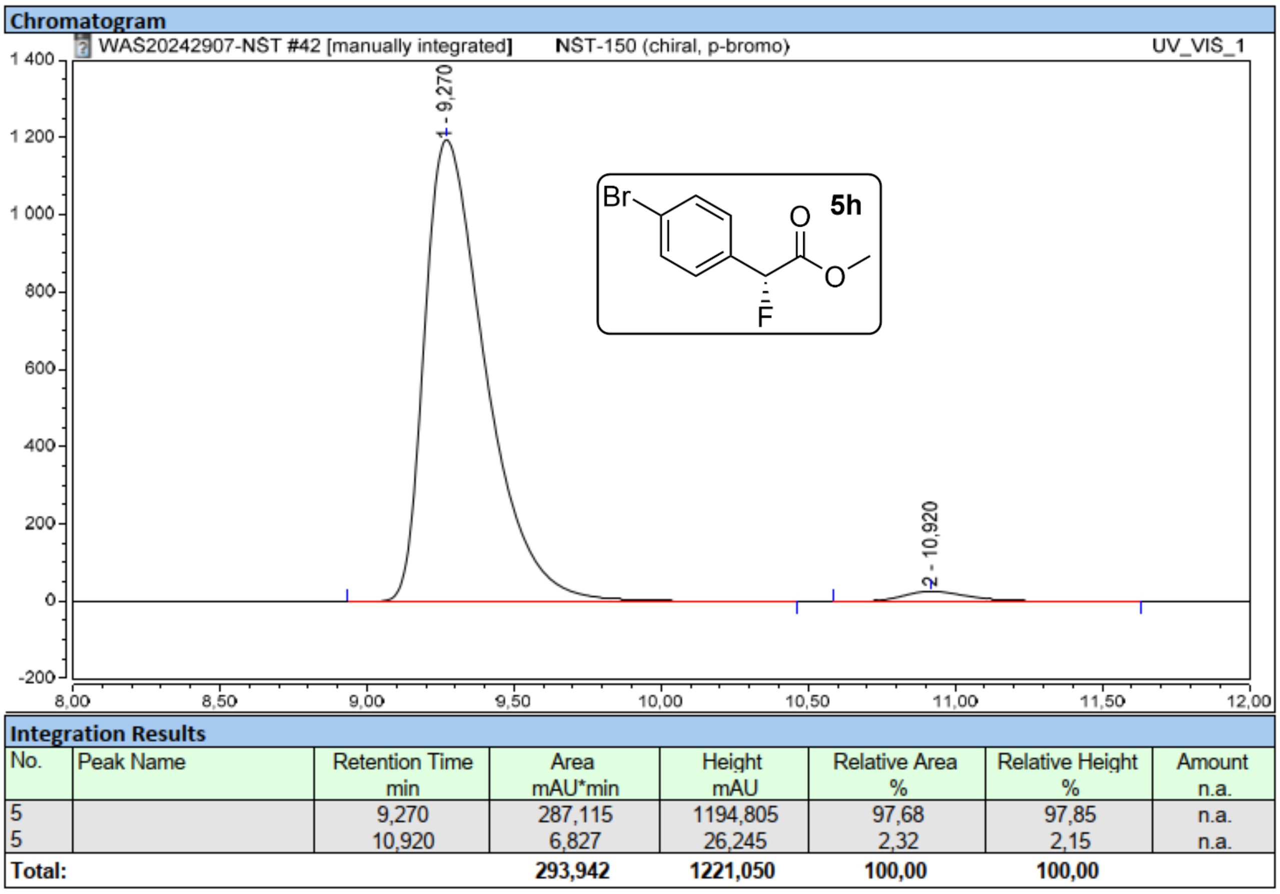

Integration Results

No.	Peak Name	Retention Time min	Area mAU*min	Height mAU	Relative Area %	Relative Height %	Amount n.a.
5		9,270	287,115	1194,805	97,68	97,85	n.a.
5		10,920	6,827	26,245	2,32	2,15	n.a.
**Total:**			**293,942**	**1221,050**	**100,00**	**100,00**	

HPLC Chromatogram of (rac.)-**5i**

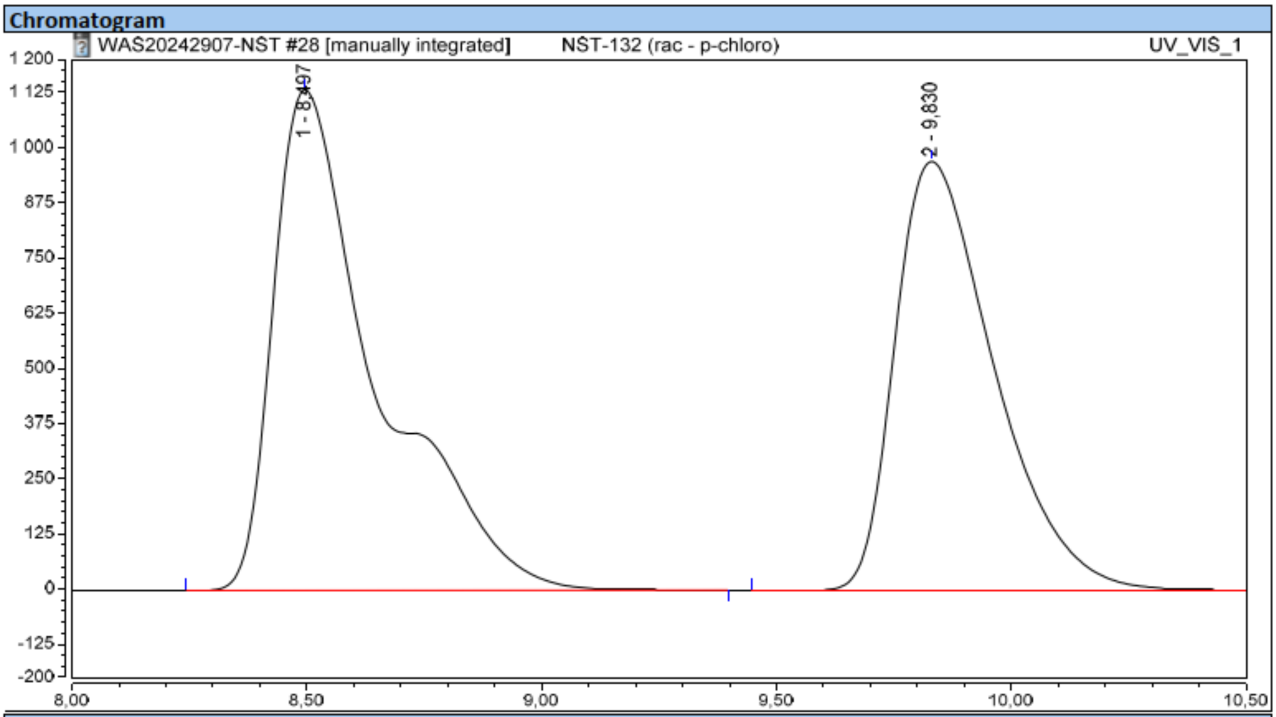

Integration Results							
No.	Peak Name	Retention Time min	Area mAU*min	Height mAU	Relative Area %	Relative Height %	Amount n.a.
5		8,497	289,361	1133,423	54,94	53,85	n.a.
5		9,830	237,283	971,275	45,06	46,15	n.a.
**Total:**			**526,644**	**2104,698**	**100,00**	**100,00**	

HPLC Chromatogram of enantioenriched-**5i**

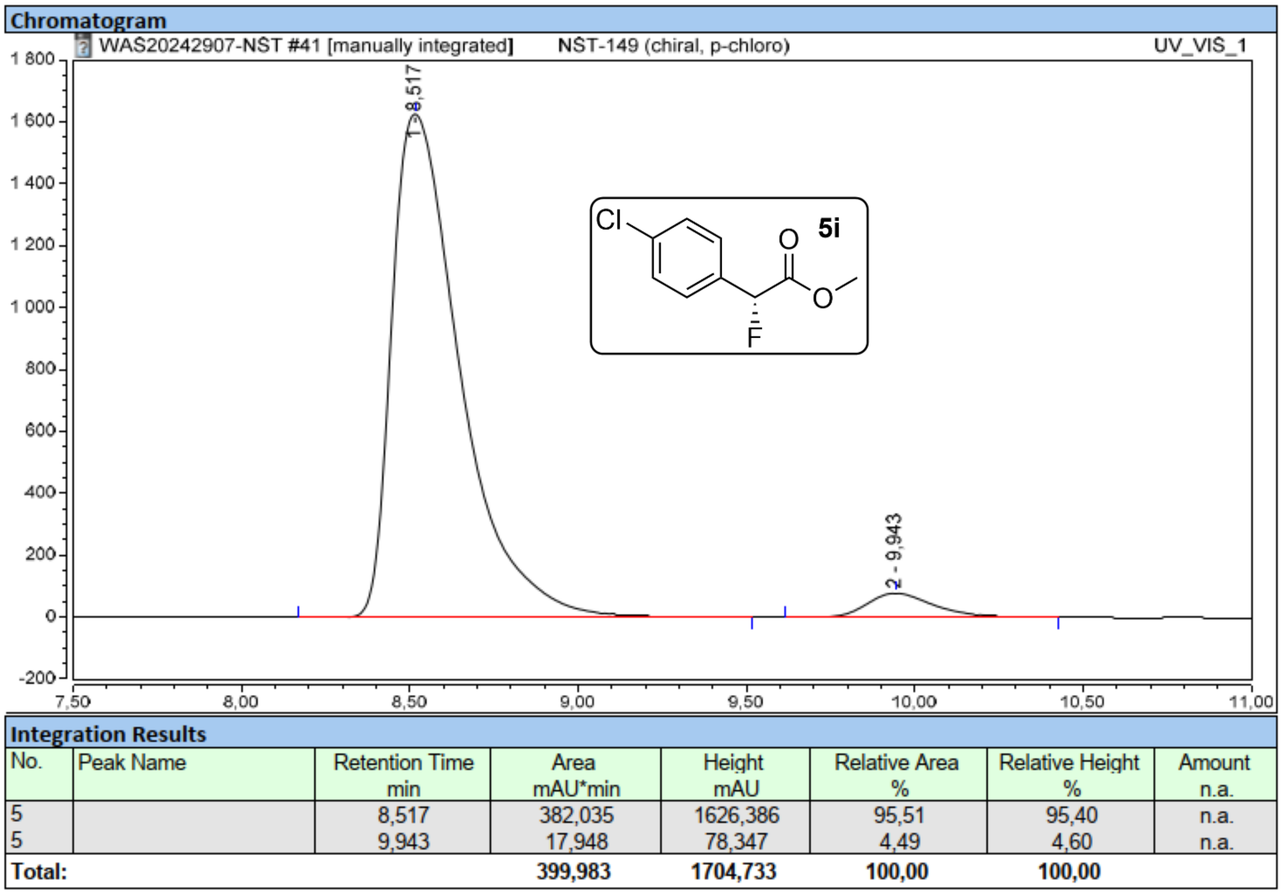

Integration Results							
No.	Peak Name	Retention Time min	Area mAU*min	Height mAU	Relative Area %	Relative Height %	Amount n.a.
5		8,517	382,035	1626,386	95,51	95,40	n.a.
5		9,943	17,948	78,347	4,49	4,60	n.a.
**Total:**			**399,983**	**1704,733**	**100,00**	**100,00**	

HPLC Chromatogram of (rac.)-**5j**

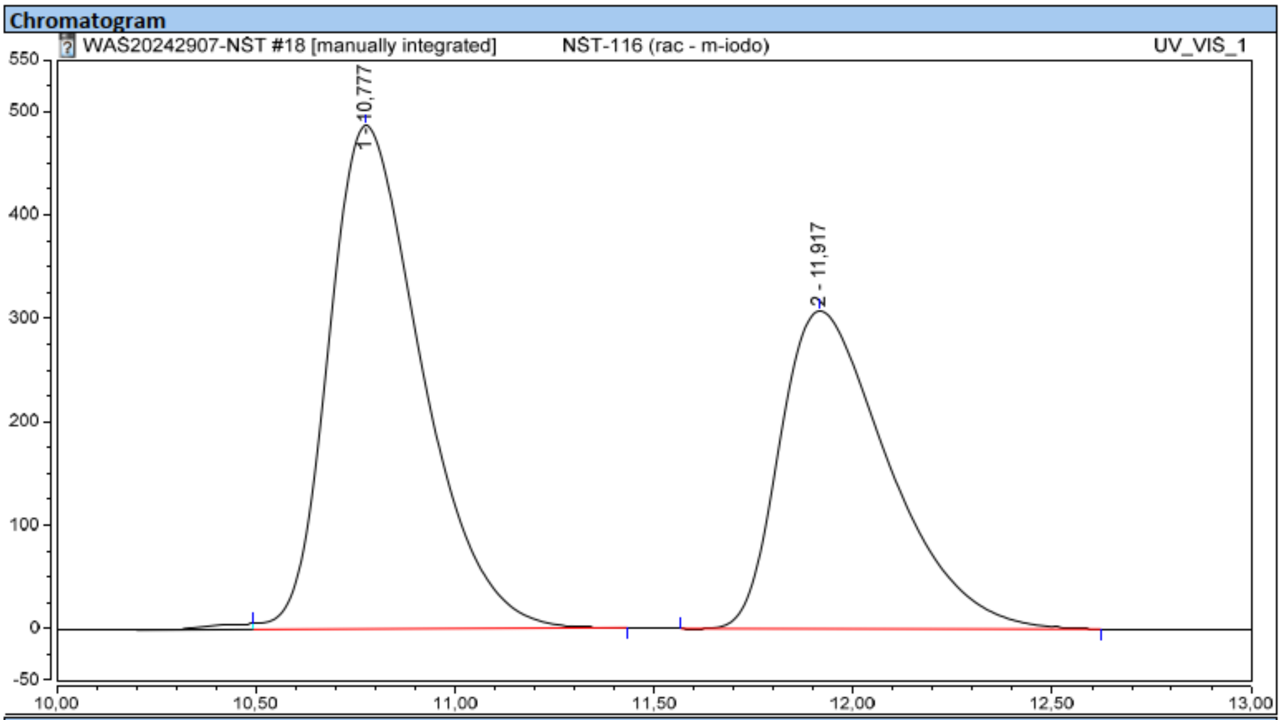

Integration Results

No.	Peak Name	Retention Time min	Area mAU*min	Height mAU	Relative Area %	Relative Height %	Amount n.a.
5		10,777	131,778	486,684	57,56	61,29	n.a.
5		11,917	97,155	307,385	42,44	38,71	n.a.
**Total:**			**228,933**	**794,069**	**100,00**	**100,00**	

HPLC Chromatogram of enantioenriched-**5j**

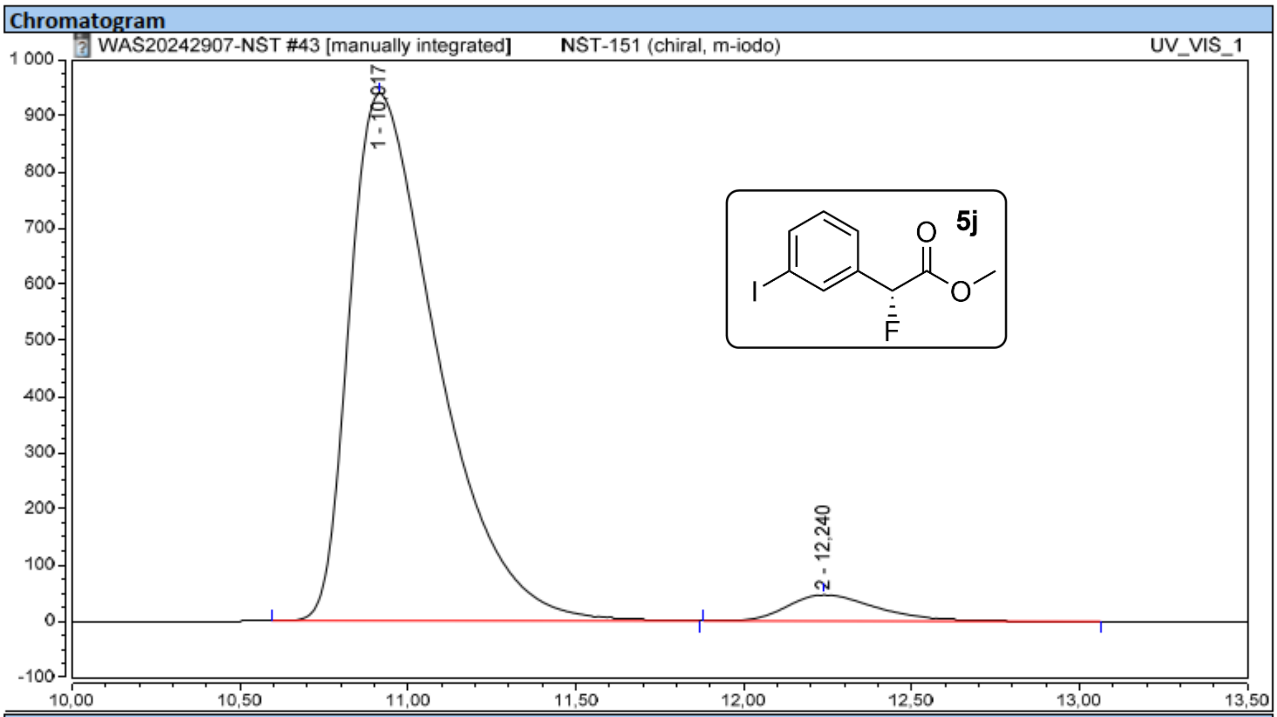

Integration Results

No.	Peak Name	Retention Time min	Area mAU*min	Height mAU	Relative Area %	Relative Height %	Amount n.a.
5		10,917	284,132	938,098	95,15	95,24	n.a.
5		12,240	14,484	46,884	4,85	4,76	n.a.
**Total:**			**298,617**	**984,982**	**100,00**	**100,00**	

HPLC Chromatogram of (rac.)-**5k**

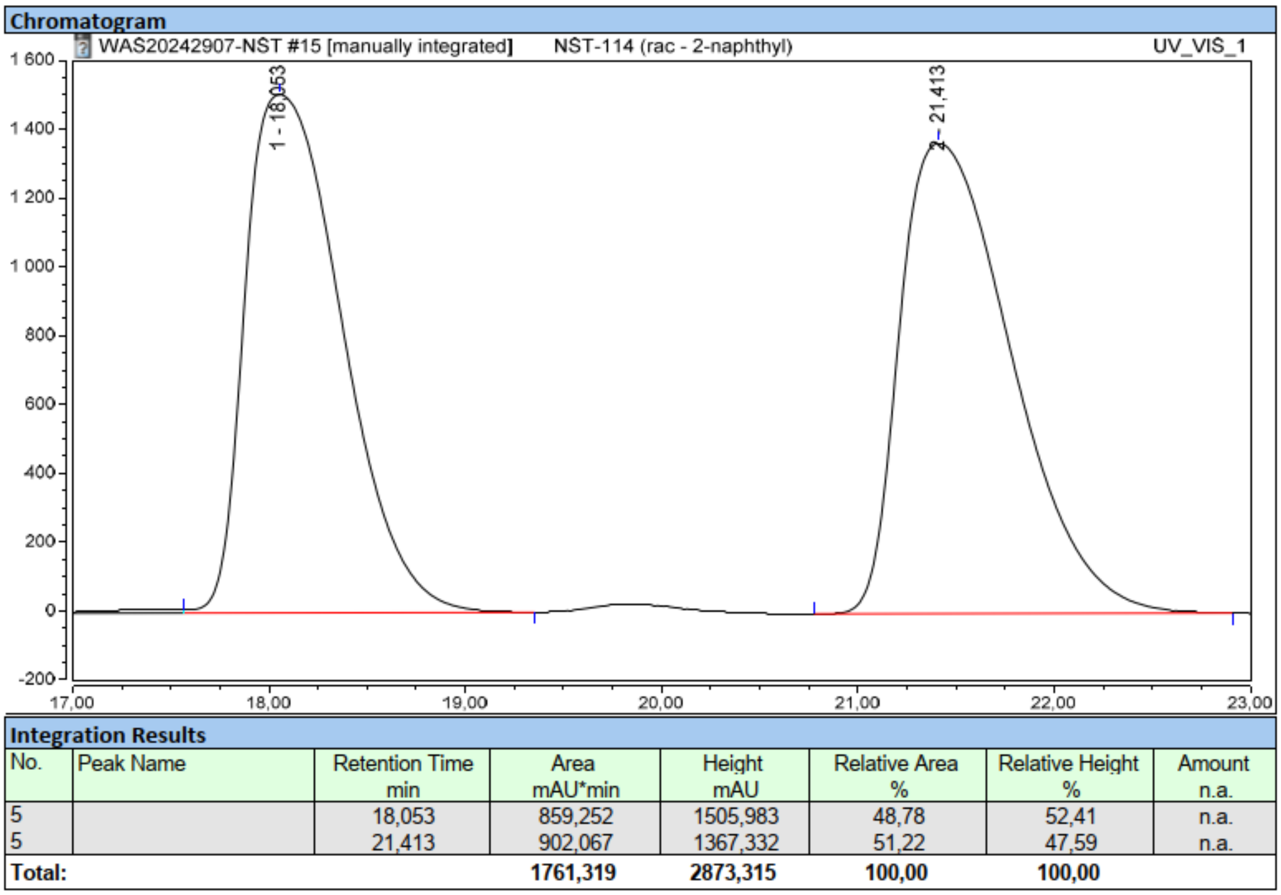

Integration Results

No.	Peak Name	Retention Time min	Area mAU*min	Height mAU	Relative Area %	Relative Height %	Amount n.a.
5		18,053	859,252	1505,983	48,78	52,41	n.a.
5		21,413	902,067	1367,332	51,22	47,59	n.a.
**Total:**			**1761,319**	**2873,315**	**100,00**	**100,00**	

HPLC Chromatogram of enantioenriched-**5k**

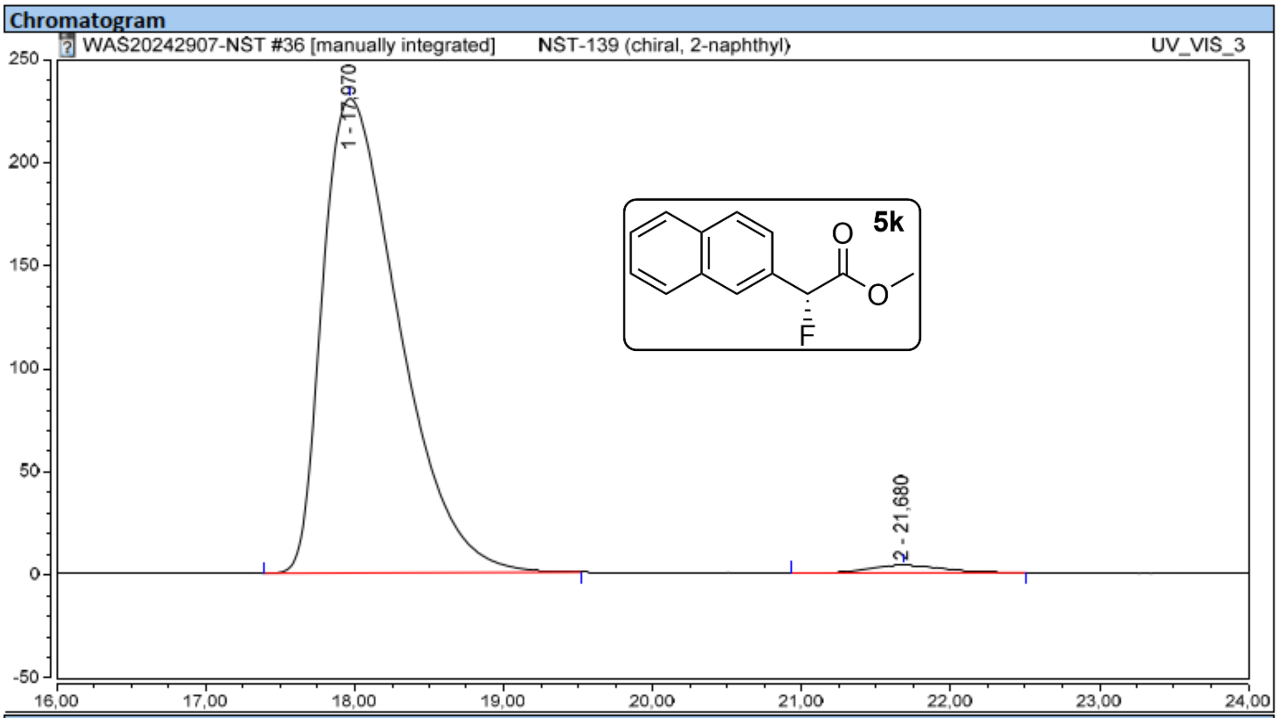

Integration Results

No.	Peak Name	Retention Time min	Area mAU*min	Height mAU	Relative Area %	Relative Height %	Amount n.a.
5		17,970	136,600	230,045	98,45	98,42	n.a.
5		21,680	2,156	3,693	1,55	1,58	n.a.
**Total:**			**138,756**	**233,738**	**100,00**	**100,00**	

HPLC Chromatogram of (rac.)-**5l**

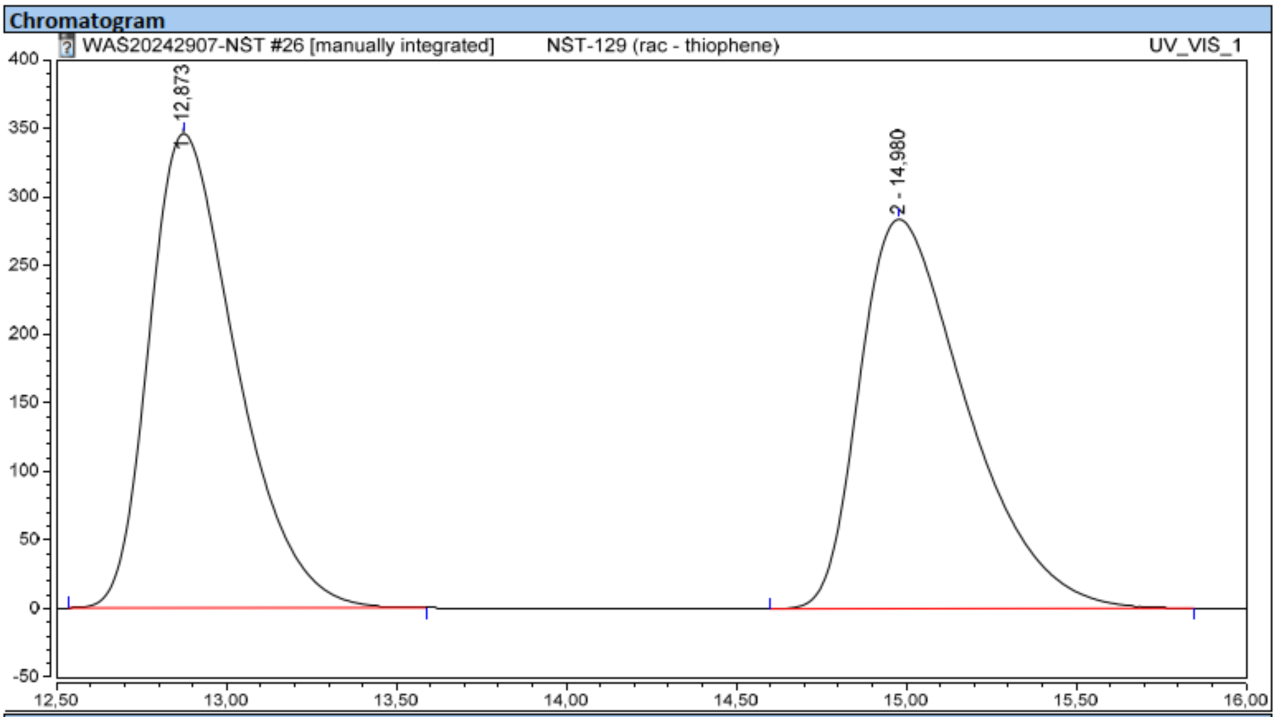

Integration Results							
No.	Peak Name	Retention Time min	Area mAU*min	Height mAU	Relative Area %	Relative Height %	Amount n.a.
5		12,873	102,601	345,531	49,72	54,91	n.a.
5		14,980	103,753	283,750	50,28	45,09	n.a.
**Total:**			**206,354**	**629,281**	**100,00**	**100,00**	

HPLC Chromatogram of enantioenriched-**5l**

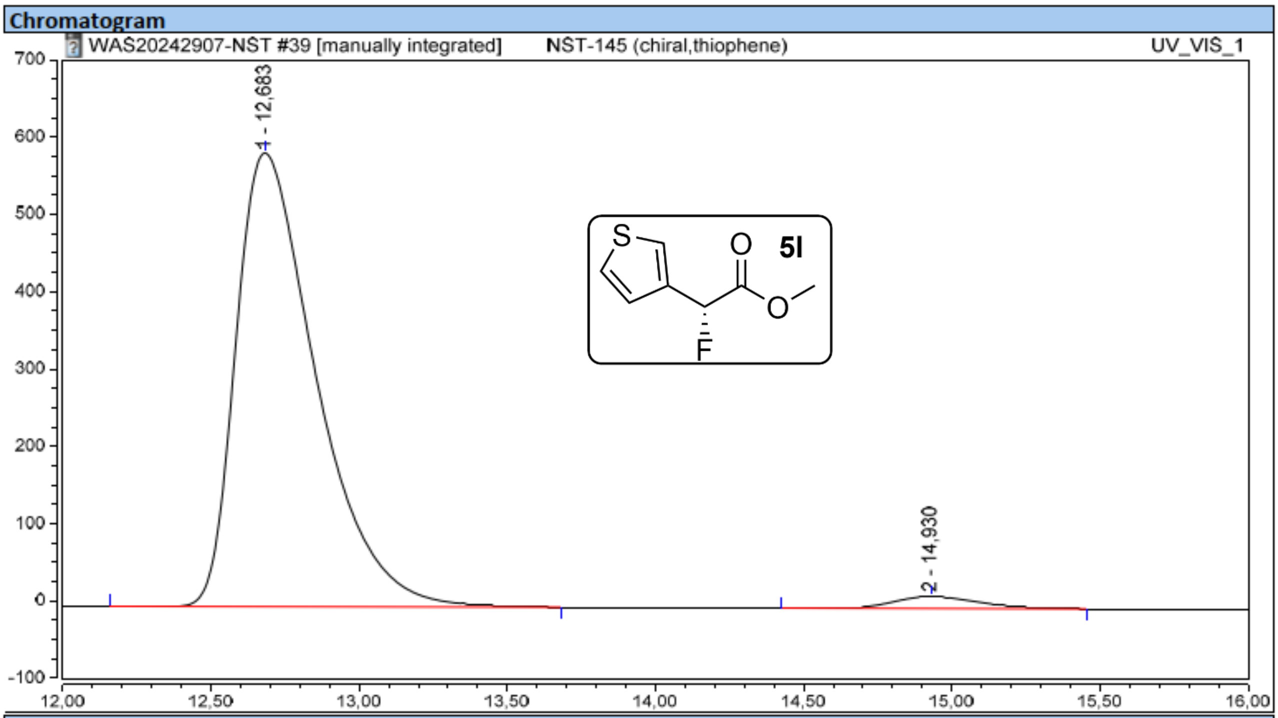

Integration Results							
No.	Peak Name	Retention Time min	Area mAU*min	Height mAU	Relative Area %	Relative Height %	Amount n.a.
5		12,683	183,926	586,857	97,24	97,41	n.a.
5		14,930	5,228	15,575	2,76	2,59	n.a.
**Total:**			**189,154**	**602,432**	**100,00**	**100,00**	

HPLC Chromatogram of (rac.)-**5m**

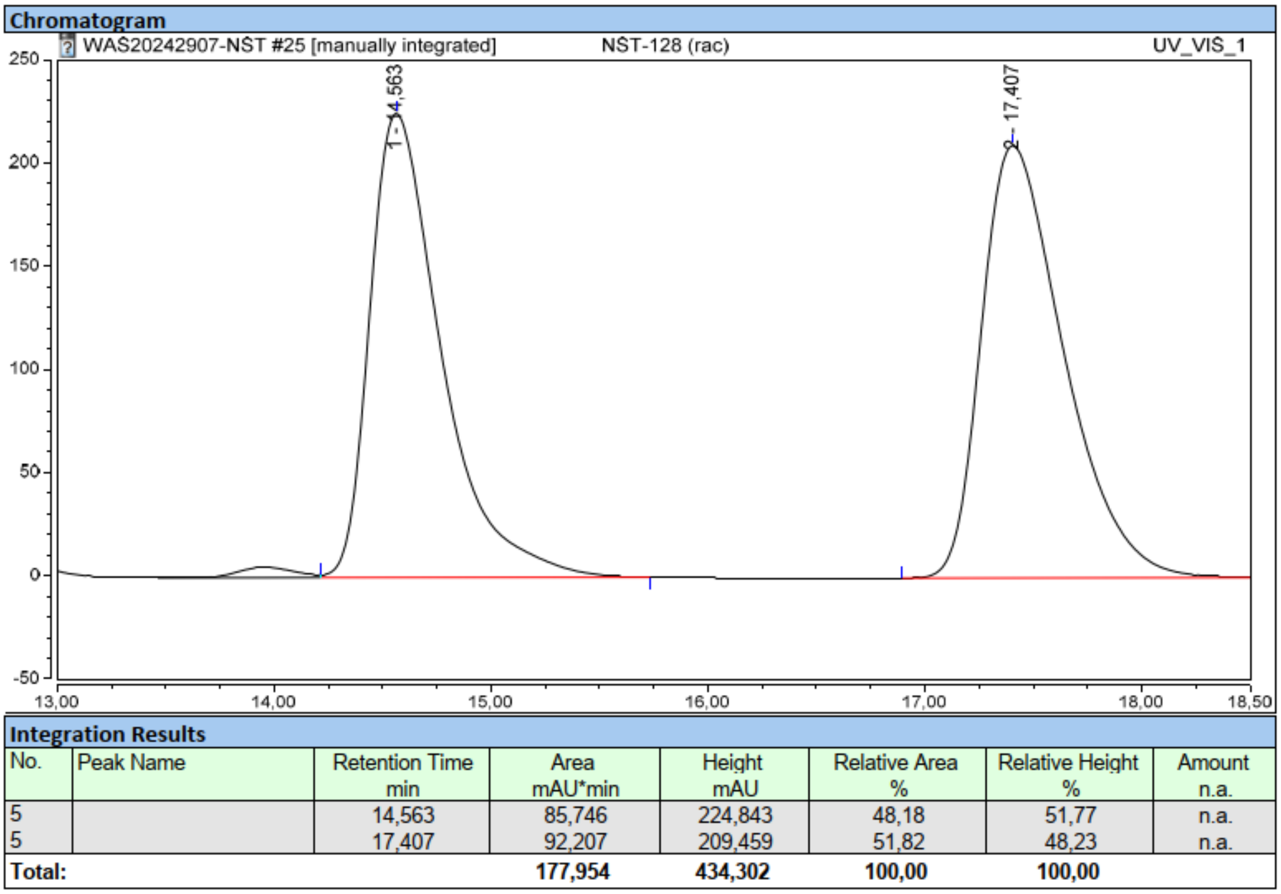

Integration Results

No.	Peak Name	Retention Time min	Area mAU*min	Height mAU	Relative Area %	Relative Height %	Amount n.a.
5		14,563	85,746	224,843	48,18	51,77	n.a.
5		17,407	92,207	209,459	51,82	48,23	n.a.
**Total:**			**177,954**	**434,302**	**100,00**	**100,00**	

HPLC Chromatogram of enantioenriched-**5m**

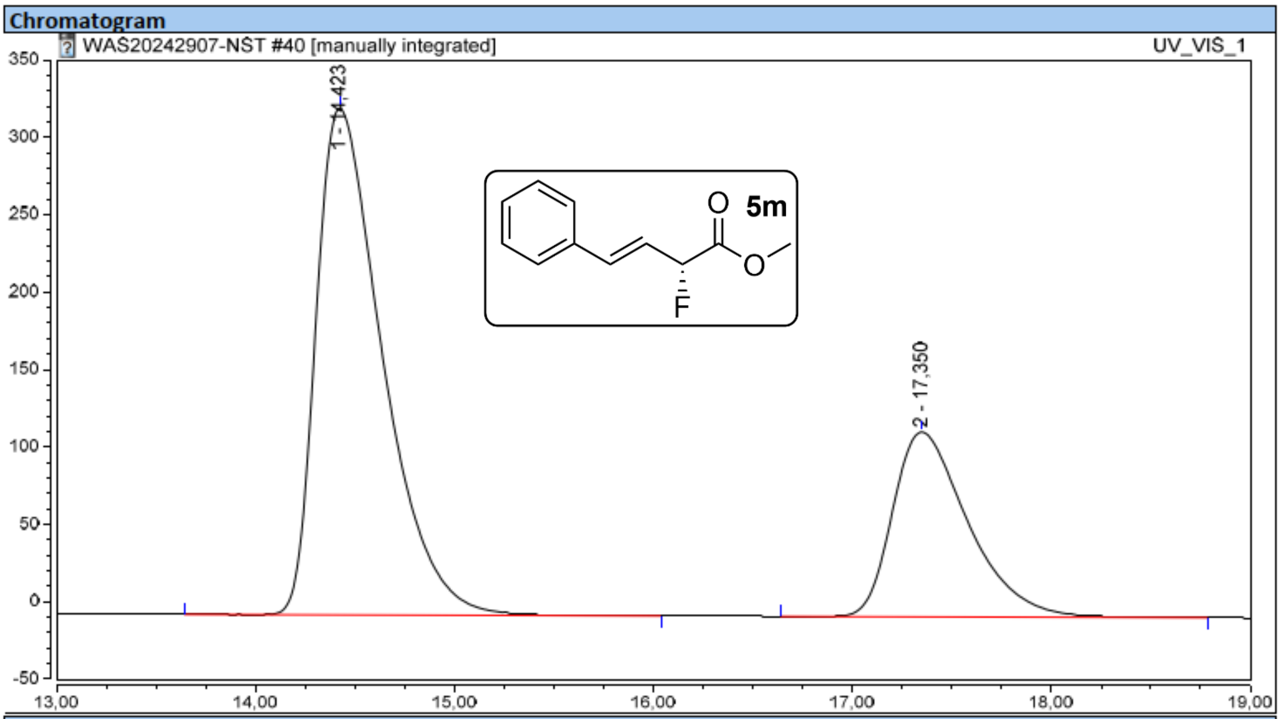

Integration Results

No.	Peak Name	Retention Time min	Area mAU*min	Height mAU	Relative Area %	Relative Height %	Amount n.a.
5		14,423	126,161	327,378	70,66	73,29	n.a.
5		17,350	52,388	119,282	29,34	26,71	n.a.
**Total:**			**178,549**	**446,660**	**100,00**	**100,00**	

HPLC Chromatogram of (rac.)-**5n**

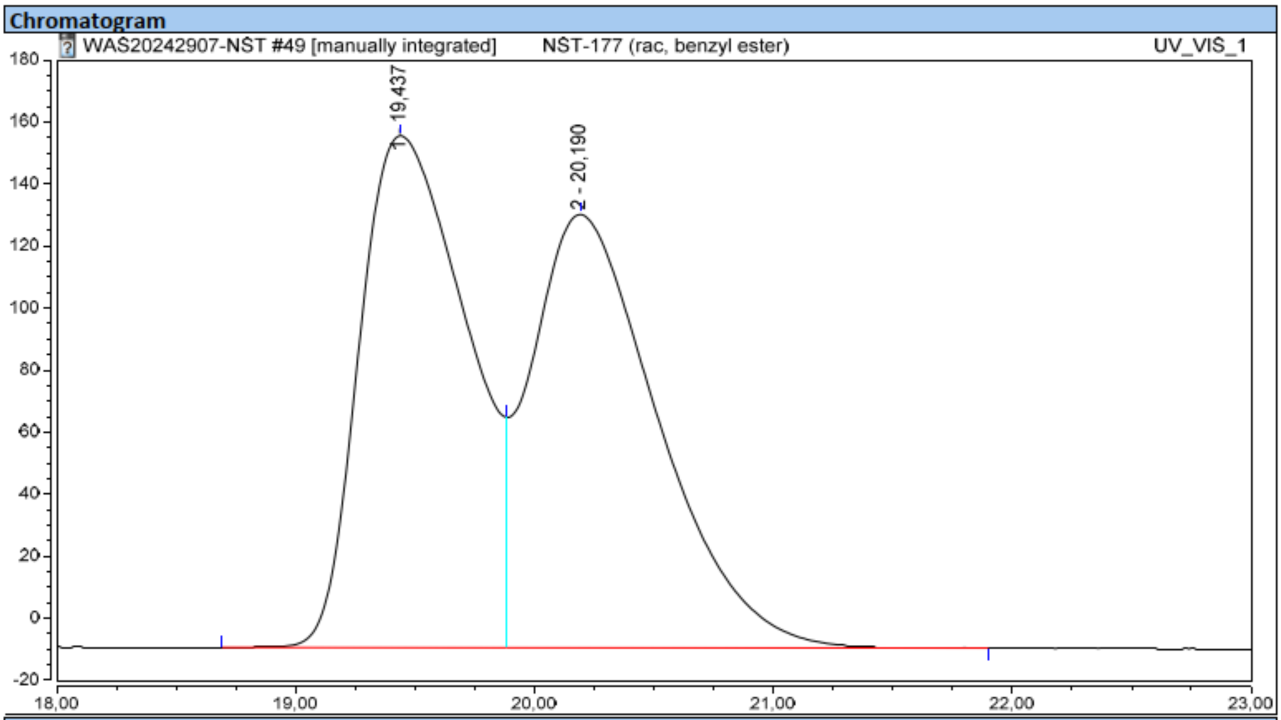

Integration Results							
No.	Peak Name	Retention Time min	Area mAU*min	Height mAU	Relative Area %	Relative Height %	Amount n.a.
5		19,437	85,626	165,020	49,70	54,17	n.a.
5		20,190	86,653	139,633	50,30	45,83	n.a.
**Total:**			**172,280**	**304,653**	**100,00**	**100,00**	

HPLC Chromatogram of enantioenriched-**5n**

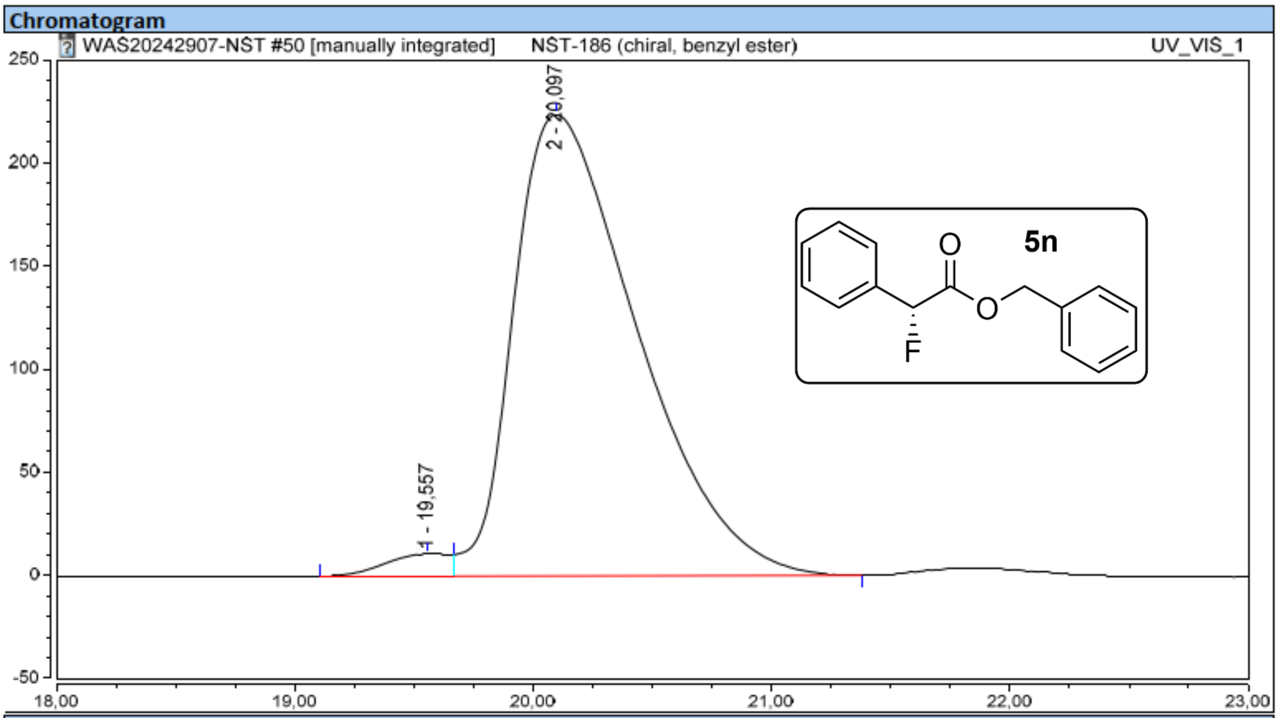

Integration Results							
No.	Peak Name	Retention Time min	Area mAU*min	Height mAU	Relative Area %	Relative Height %	Amount n.a.
5		19,557	3,347	10,809	2,43	4,61	n.a.
5		20,097	134,552	223,790	97,57	95,39	n.a.
**Total:**			**137,899**	**234,599**	**100,00**	**100,00**	

HPLC Chromatogram of (rac.)-**5o**

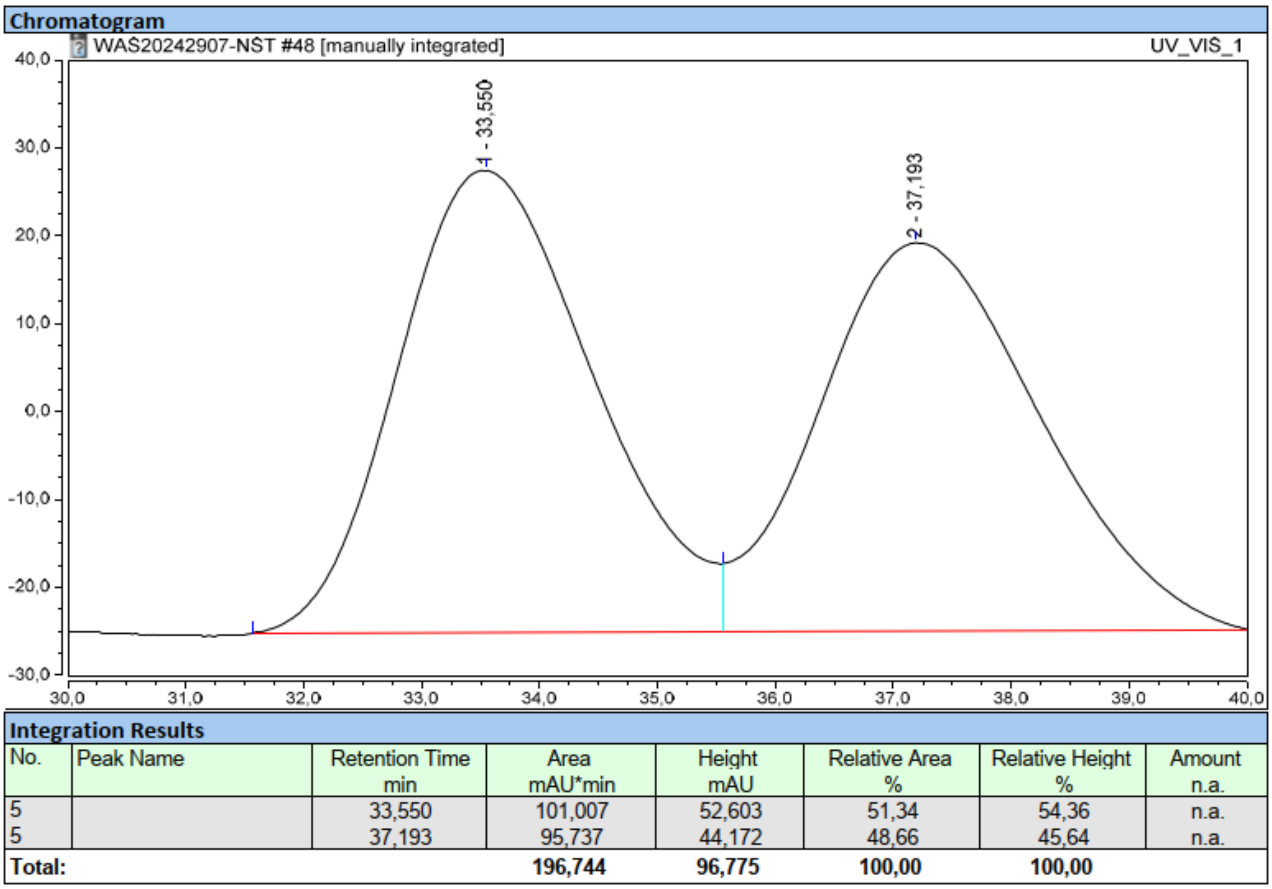

Integration Results

No.	Peak Name	Retention Time min	Area mAU*min	Height mAU	Relative Area %	Relative Height %	Amount n.a.
5		33,550	101,007	52,603	51,34	54,36	n.a.
5		37,193	95,737	44,172	48,66	45,64	n.a.
**Total:**			**196,744**	**96,775**	**100,00**	**100,00**	

HPLC Chromatogram of enantioenriched-**5o**

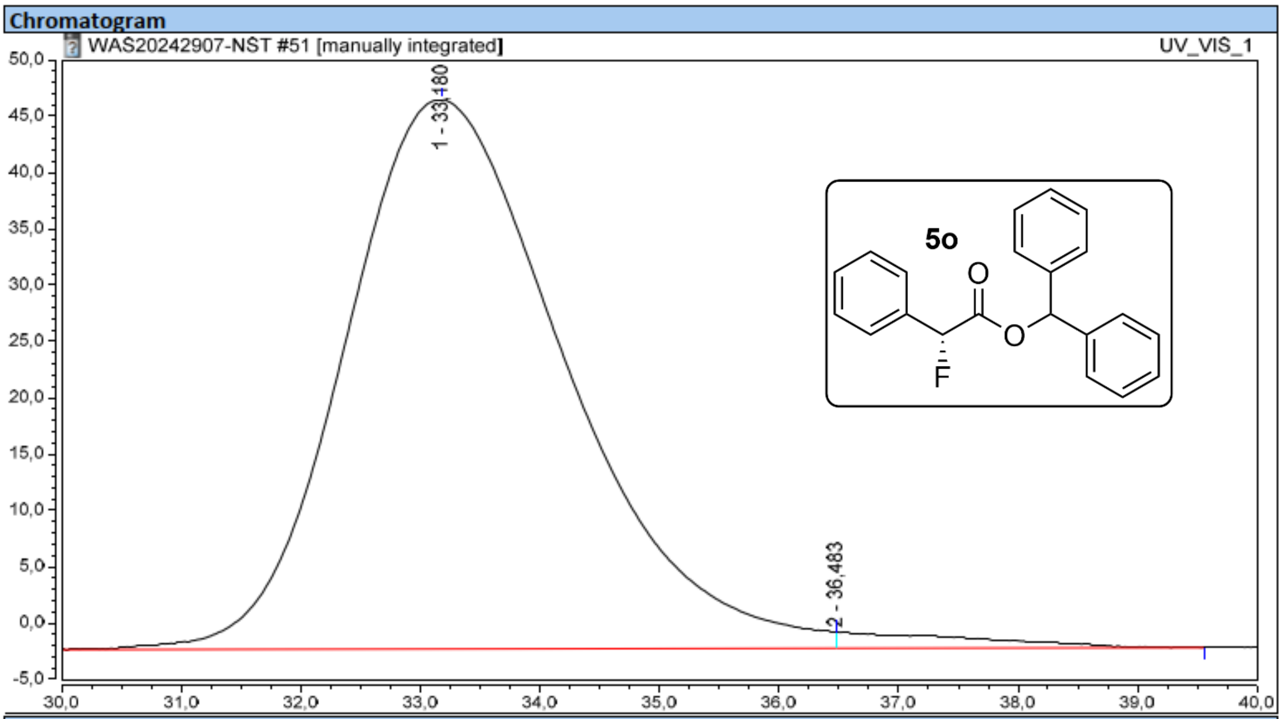

Integration Results

No.	Peak Name	Retention Time min	Area mAU*min	Height mAU	Relative Area %	Relative Height %	Amount n.a.
5		33,180	103,919	48,779	98,20	97,14	n.a.
5		36,483	1,902	1,438	1,80	2,86	n.a.
**Total:**			**105,821**	**50,218**	**100,00**	**100,00**	

HPLC Chromatogram of (rac.)-**6**

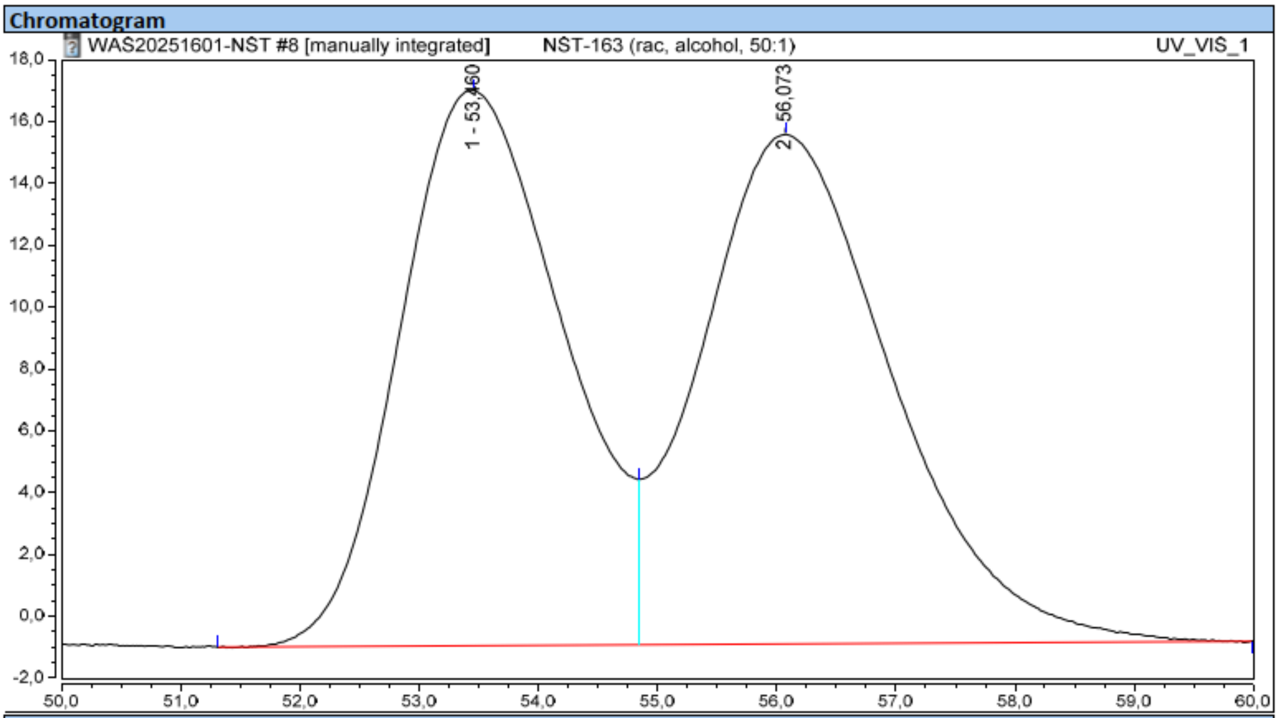

Integration Results

No.	Peak Name	Retention Time min	Area mAU*min	Height mAU	Relative Area %	Relative Height %	Amount n.a.
5		53,460	28,444	17,965	48,19	52,16	n.a.
5		56,073	30,584	16,475	51,81	47,84	n.a.
**Total:**			**59,028**	**34,440**	**100,00**	**100,00**	

HPLC Chromatogram of enantioenriched-**6**

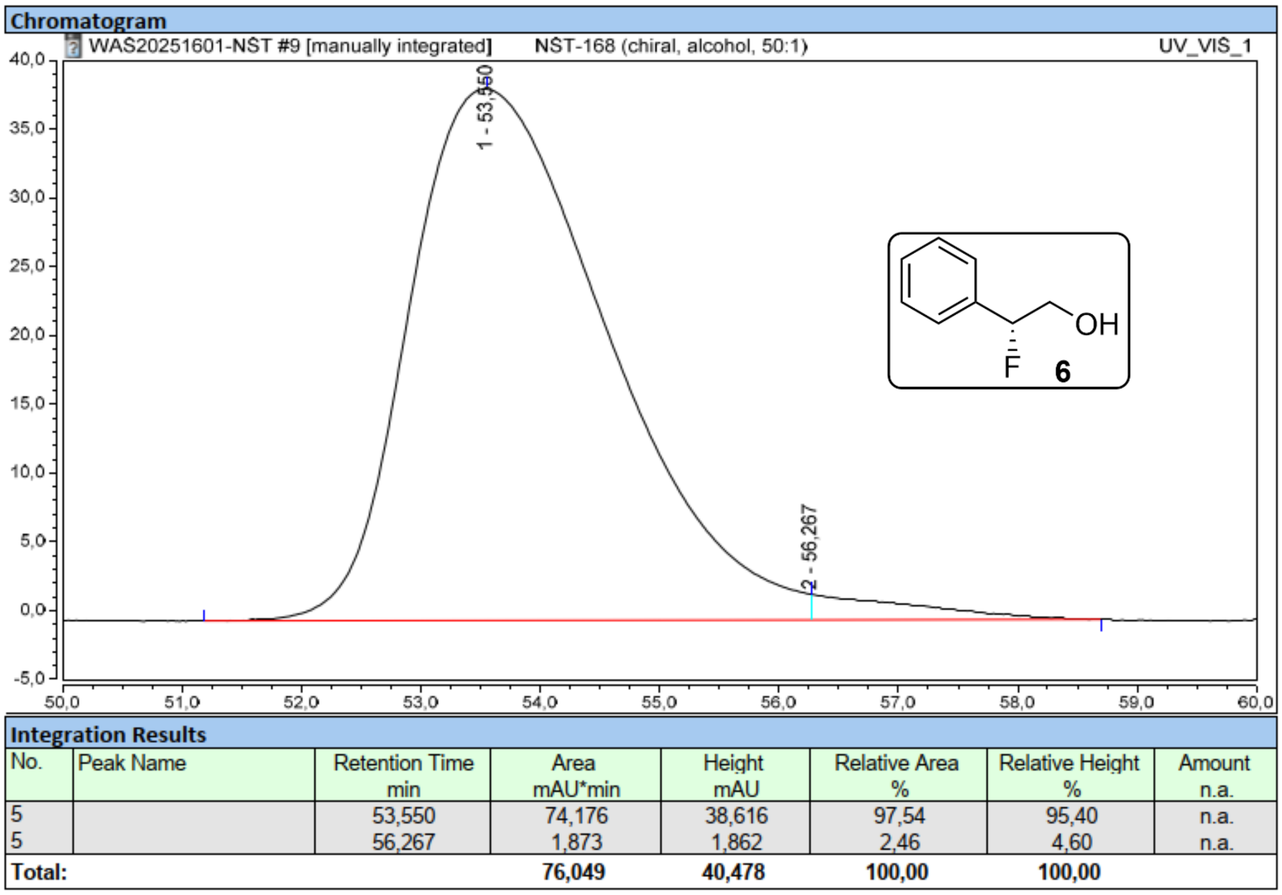

Integration Results

No.	Peak Name	Retention Time min	Area mAU*min	Height mAU	Relative Area %	Relative Height %	Amount n.a.
5		53,550	74,176	38,616	97,54	95,40	n.a.
5		56,267	1,873	1,862	2,46	4,60	n.a.
**Total:**			**76,049**	**40,478**	**100,00**	**100,00**	

HPLC Chromatogram of (rac.)-**7**

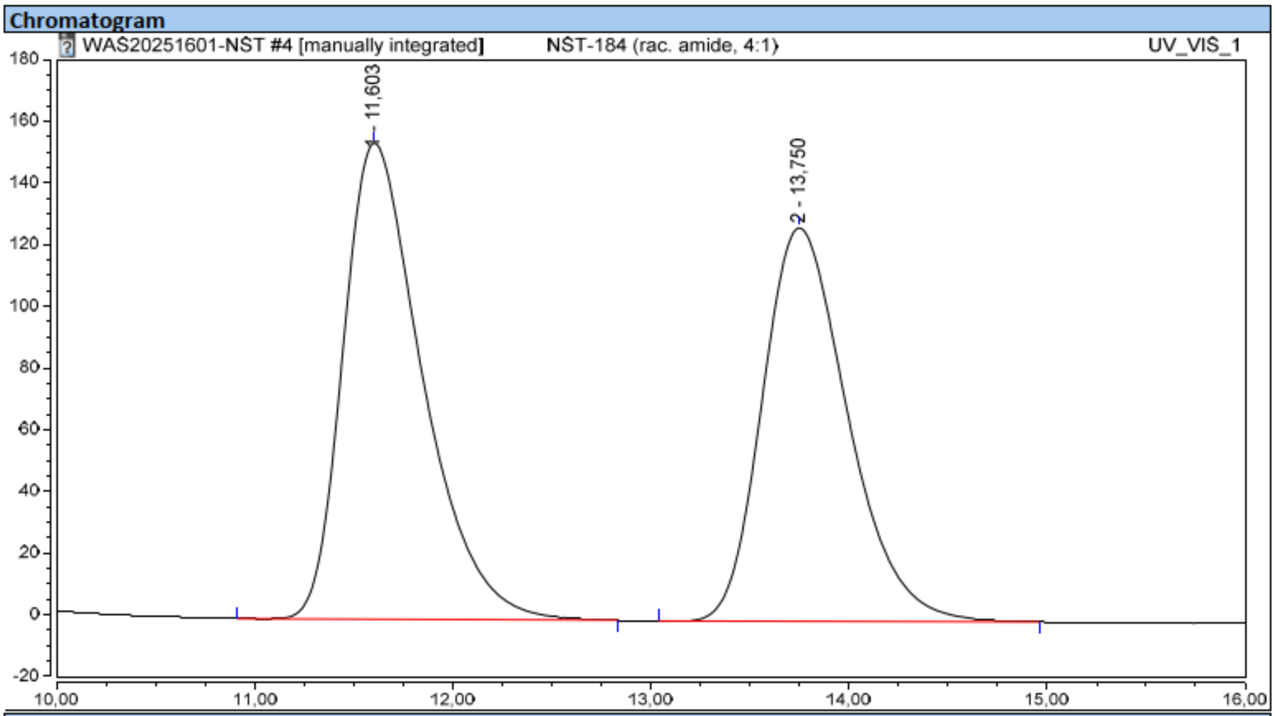

Integration Results

No.	Peak Name	Retention Time min	Area mAU*min	Height mAU	Relative Area %	Relative Height %	Amount n.a.
5		11,603	71,506	154,342	52,89	54,75	n.a.
5		13,750	63,682	127,536	47,11	45,25	n.a.
**Total:**			**135,189**	**281,878**	**100,00**	**100,00**	

HPLC Chromatogram of enantioenriched-**7**

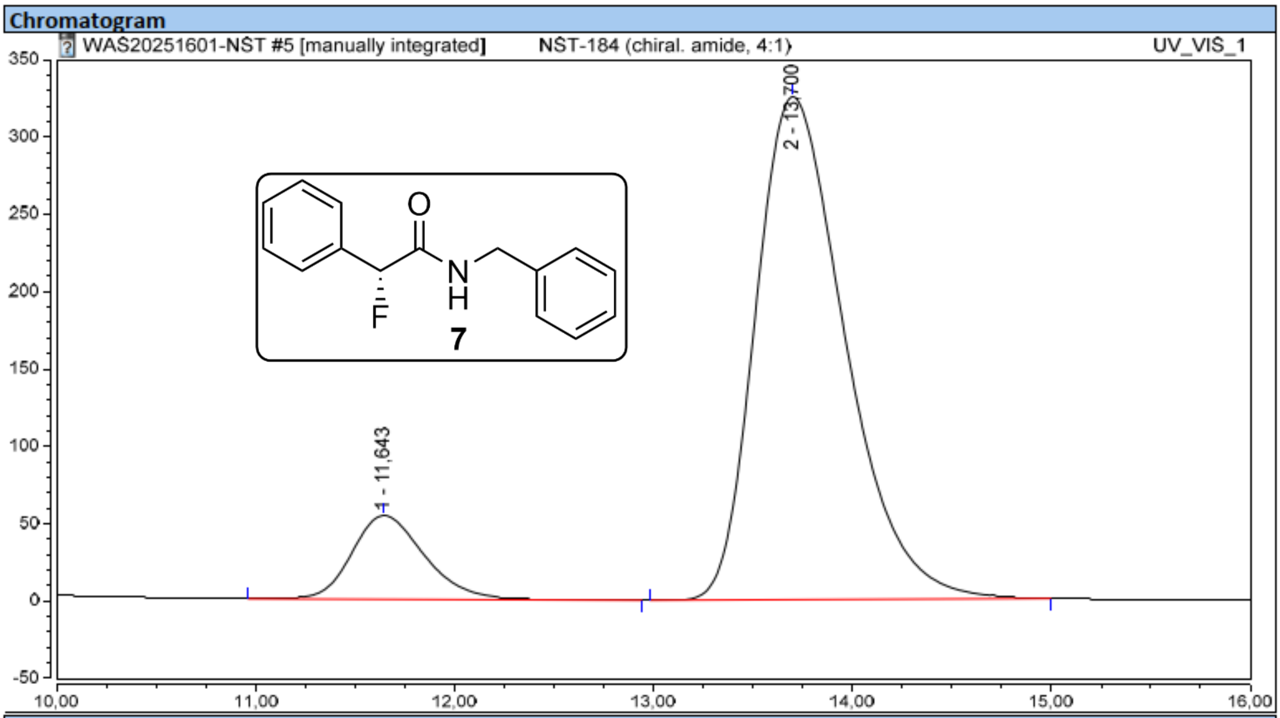

Integration Results

No.	Peak Name	Retention Time min	Area mAU*min	Height mAU	Relative Area %	Relative Height %	Amount n.a.
5		11,643	23,040	54,143	12,00	14,28	n.a.
5		13,700	168,927	325,116	88,00	85,72	n.a.
**Total:**			**191,966**	**379,259**	**100,00**	**100,00**	
